# A Comprehensive Review of Aminochalcones

**DOI:** 10.3390/molecules25225381

**Published:** 2020-11-17

**Authors:** Rimsha Irfan, Shikufa Mousavi, Meshari Alazmi, Rahman Shah Zaib Saleem

**Affiliations:** 1 Department of Chemistry and Chemical Engineering, SBA School of Sciences and Engineering, Lahore University of Management Sciences, DHA, Lahore 54792, Pakistan; 20100130@lums.edu.pk (R.I.); 21100279@lums.edu.pk (S.M.); 2 Department of Information and Computer Science, College of Computer Science and Engineering, University of Ha’il, P.O. Box 2440, Ha’il 81481, Saudi Arabia; Ms.alazmi@uoh.edu.sa

**Keywords:** chalcone, aminochalcone, biological, cytotoxic, anticancer, antimicrobial, antiviral

## Abstract

Chalcones, members of the flavonoid family, display a plethora of interesting biological activities including but not limited to antioxidant, anticancer, antimicrobial, anti-inflammatory, and antiprotozoal activities. The literature cites the synthesis and activity of a range of natural, semisynthetic, and synthetic chalcones. The current review comprehensively covers the literature on amino-substituted chalcones and includes chalcones with amino-groups at various positions on the aromatic rings as well as those with amino-groups containing mono alkylation, dialkylation, alkenylation, acylation, and sulfonylation. The aminochalcones are categorized according to their structure, and the corresponding biological activities are discussed as well. Some compounds showed high potency against cancer cells, microbes, and malaria, whereas others did not. The purpose of this review is to serve as a one-stop location for information on the aminochalcones reported in the literature in recent years.

## 1. Introduction

**Figure 1:**
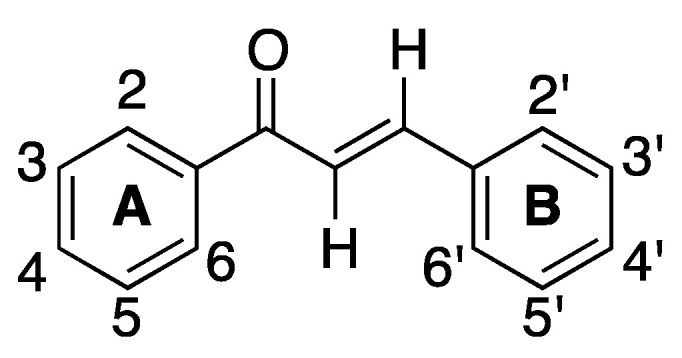
The general structure of chalcone.

The (*E*)-1,3-diphenyl-2-propene-1-one scaffold, also known as “chalcone” is not only a key structure for synthetic manipulations but also a constituent of many natural products. Chalcones are considered precursors of flavonoids and isoflavones and can act as synthons for a range of heterocycles with pharmaceutical importance [1,2,3]. The chemical structure of a chalcone consists of two aromatic rings on either side of an α, ß-unsaturated carbonyl system (Figure 1).

**Scheme 1:**
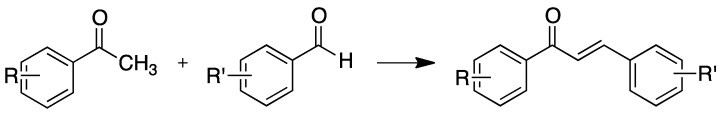
General scheme for the synthesis of chalcone.

The most common method to synthesize chalcones is the Claisen-Schmidt condensation method, which uses either base or acid-catalyzed condensation of the aldehyde and ketone, that is typically followed by in situ dehydration (Scheme 1). 

Chalcones are naturally abundant in fruits, vegetables, teas, spices, and other natural foods. Both naturally occurring and synthetic chalcones display bioactivities across the spectrum [4], including but not limited to anticancer [5,6,7,8,9,10,11], anti-inflammatory [12,13,14], antimalarial [15,16,17,18], antibacterial [19,20,21], antioxidant [22,23,24], antifungal [25,26,27], antimicrobial [28,29], antiprotozoal [30,31], anticonvulsant [32], and antiulcer [33,34] activities. Several chalcones isolated from plants have been approved for clinical trials as anticancer and antiulcer drugs, for example, metochalcone and sofalcone [35].

**Figure 2:**
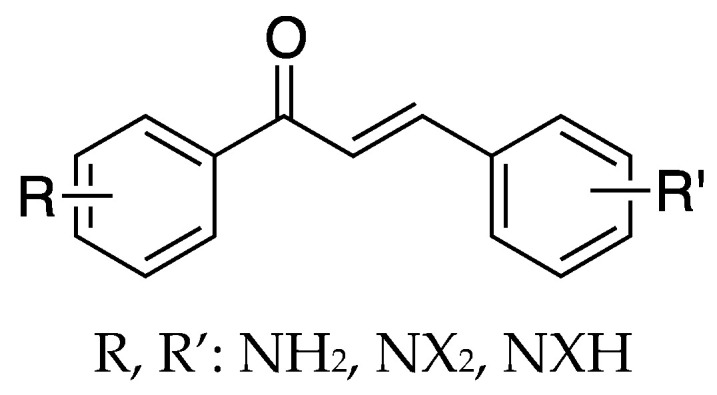
Aminochalcones.

Aminochalcones constitute an important class of chalcones that contain an -NR_2_ group on either ring A or ring B of the chalcone (Figure 2). These compounds have been synthesized and evaluated for their biological significance by various groups recently. The cytotoxic activity of aminochalcones has been an area of interest due to the possibility of an enhanced electrophilic attack on cellular proteins through hetero-Michael addition leading to a covalent bond. The covalent inhibitors are an emerging area of drug discovery, and the aminochalcones hold the potential to serve as leads for the development of inhibitors targeting specific cellular proteins. 

The purpose of this review article is to summarize and list the aminochalcones reported in the literature. We aim to list the developments and pharmacological significance of aminochalcones by presenting a comprehensive list of the compounds with their significant biological activities. To understand the structure–activity relationship of aminochalcones, we will be categorizing these by the substitution position of the amino-group on both rings. Other categories will include *N*-alkyl, *N*-alkenyl, *N*-acyl, and *N*-sulfonyl aminochalcones. Thus the article will provide the versatile biological activities of aminochalcones and their relationship with the structure.

## 2. -NH_2_-Substituted Aminochalcones

### 2.1. Substitution on Ring A 

#### 2.1.1. 4-Aminochalcone and Derivatives 

**Figure 3:**
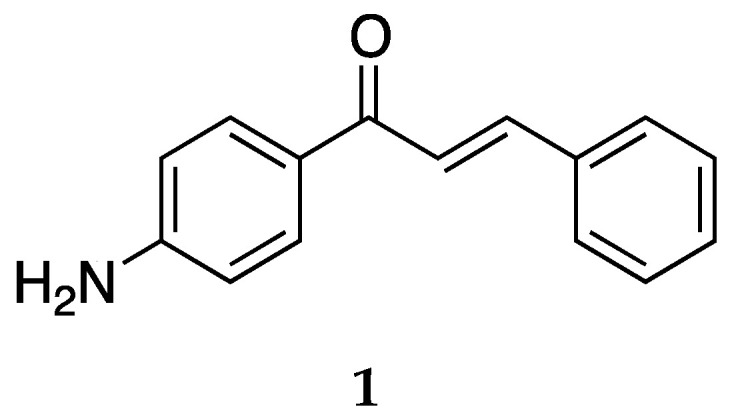
Chemical structure of 4-aminochalcone.

The most studied aminochalcone **1** contains a -NH_2_ substitution at position 4 on ring A (Figure 3). The amino group on ring A increases the magnitude of the positive charge on the olefinic carbon atom adjacent to ring B. This has been proposed to enhance the electrophilic attack of these compounds on cancerous cells [36].

Compound **1** has been extensively studied for its anticancer activity on multiple cell lines along with antibacterial and antifungal activities. Dimmock et al. [36] synthesized and evaluated the cytotoxic activities of several 4-aminochalcones, including **1** against the human Molt 4/C8, CEM T-lymphocytes, murine P388, and L1210 cancer cell lines. Compound **1** has also been prepared and tested by Romagnoli et al. [37] on five different cancer cell lines. Moreover, other groups have also tested **1** on several other cancer cell lines. The IC_50_ values are summarized in Table 1. 

The IC_50_ values of a simple chalcone against the Molt 4/C8, CEM, P388, and L1210 cell lines are 12.7, 12.5, 9.63, and 41.4 µM, respectively [36]. The IC_50_ values against the same cell lines for **1** are lower than this, which shows that the presence of an amino group at position 4 leads to a more potent compound. Of all the compounds tested by Novilla et al. [41], compound **1** showed the highest level of cytotoxicity against the K562 cell line with an IC_50_ value of 5.87 ± 0.15 µg/mL. Compound **1** showed better activity against this cell lines when compared with Accutane (IC_50_ = 39.47 ± 3.14 µg/mL) and comparable activity to imatinib (IC_50_ = 2.67 ± 0.53 µg/mL). Compound **1** was also more potent than imatinib (IC_50_ = 16.59 ± 0.77 µg/mL) for the HL-60 cell line. Overall, the IC_50_ values against multiple cancer cell lines show the potency of compound **1** against cancer cells. Novilla et al. [41] have also tested **1** on lymphocytes with an IC_50_ value of 4.33 ± 0.97 µg/mL. Compound **1** was tested against the canine malignant histiocytic cell line DH82 [43] and showed a mean percentage cell death of DH82 at 59.2 ± 4.2 in 15.0 µg/mL of the compound. This activity was higher than the simple chalcone (mean percent = 27.7 ± 7.3), which was attributed to the presence of the amino group in **1**. The IC_50_ values determined by Mai et al. [38] against **1** showed that the compound had cytotoxic properties as it was potent against a few cell lines. The IC_50_ value for **1** against the normal embryonic kidney HEK-293 cells was 95.34 ± 2.48 µM, and the selectivity ratio with HT-29 was <1. Thus, compound **1** could also be toxic to normal cells if used as a cancer therapy. 

Along with its cytotoxic activity, **1** has also been tested for its activity against microbes. The antimicrobial activity of **1** against *Escherichia coli* ATCC 25923, *Staphylococcus aureus* ATCC 25922, and *Candida albicans* ATCC 10231 was also reported by Suwito et al. [40] The diameter of inhibition values for these microbes were reported to be 10.25 ± 0.13 mm, 9.59 ± 0.16 mm, and 9.69 ± 0.02 mm, respectively, tested at a concentration of 500 µg/mL. The inhibition activity of **1** against *Escherichia coli* ATCC 25923 and *Candida albicans* ATCC 10231 was as strong as the antibiotic sulfamerazine and as strong as sulfadiazine against *Staphylococcus aureus* ATCC 25922. The antibacterial and antifungal activities against different strains were also tested by Kozłowska et al. [42], as shown in Table 2. 

Compound **1** causes complete growth inhibition in the case of *S. aureus* DSM799 and *A. niger* DSM1957 microbial strains and showed significant prevention of growth for the other strains [42]. The results of these investigations [40,42] show **1** as a promising antibacterial agent. 

Apart from cytotoxic and antimicrobial activities, compound **1** has also been tested for its inhibitory activity against the chlorinating activity of the myeloperoxidase (MPO) enzyme [44]. MPO has been explored as a target for anti-inflammatory therapy due to its ability to generate hypochlorous acid. Compound **1** was identified as a potent inhibitor of the chlorinating activity of MPO with an IC_50_ value of 0.26 ± 0.04 µmol/L in a cell-free, purified MPO system. Interestingly, for a simple chalcone and aniline, both did not show any inhibition to the chlorinating activity of MPO, thus showing that the presence of both the amino and chalcone groups is important for any significant activity. The activity of **1** has also been compared with 4-hydroxychalcone, which also did not show any inhibitory activity. Thus, the presence of an amino group seems important compared to an electron-donating group. Moreover, the IC_50_ value for **1** is comparable to 5-fluorotryptamine, which is considered a potent MPO inhibitor.

**Figure 4:**
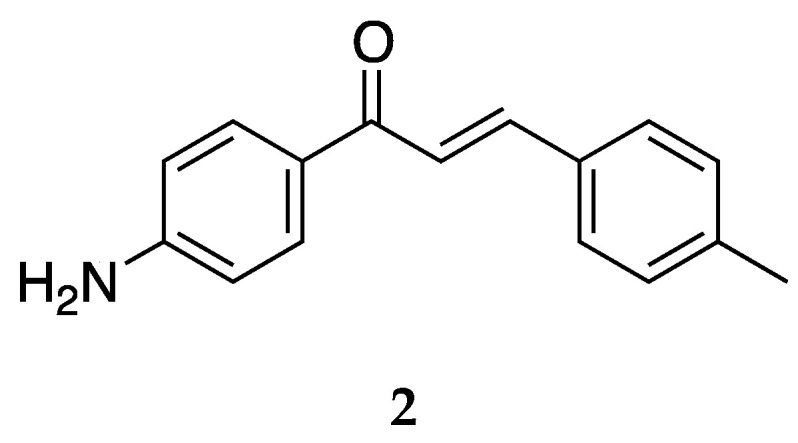
Chemical structure of 4-aminochalcone derivatives with methylation on ring B.

**Figure 5:**
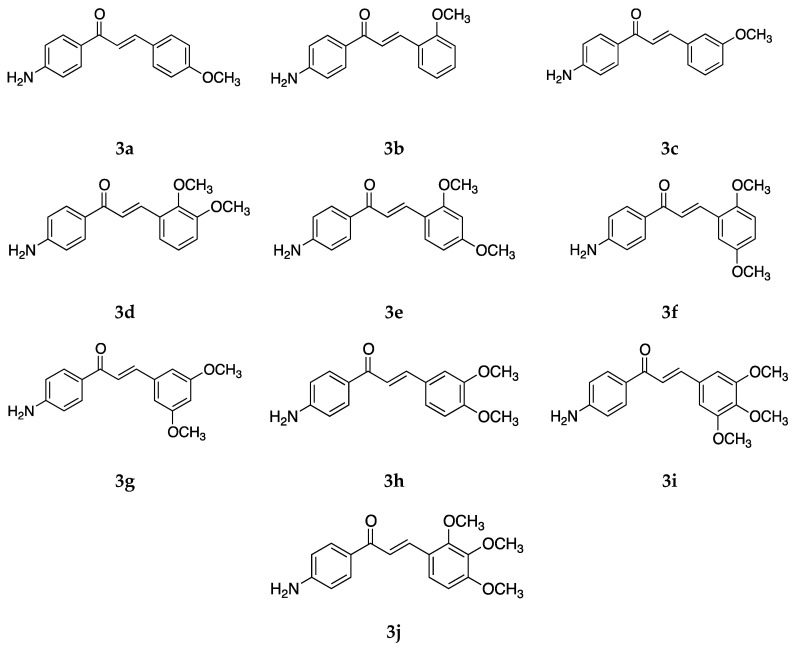
Chemical structures of 4-aminochalcone derivatives with methoxy substitutions on ring B.

Several derivates of 4-aminochalcone have been synthesized by various groups over the years, with one of them being derivates with aliphatic alkylation on position 4 of ring B (Figure 4 and Figure 5). 

Compound **2** was tested for its cytotoxic activities by Dimmock et al. [36], Santos et al. [43], and Santos et al. [39], shown in Table 3. 

Aminochalcone **2** has IC_50_ values similar to **1**; thus, the anticancer activity of 4-aminochalcone does not increase or significantly decrease with the addition of a methyl group on ring B. Santos et al. [43] also reported the activity against canine malignant histiocytic cell line DH82 for **2**, which was 74.7 ± 1.7%. This value is higher than **1**, showing that the addition of a methyl group on ring B increased the potency of the compound.

In addition to the cytotoxic activity, the antioxidant activity of **2** has also been tested by Prasad et al. [45]. Many degenerative diseases such as atherosclerosis, cancer, inflammatory joint disease, asthma, diabetes, senile dementia, and degenerative eye disease originate from deleterious free radical reactions. Most free radical damage is caused by reactive oxidant species (ROS) which can damage genetic material, cause lipid peroxidation in cell membranes, and inactivate membrane-bound enzymes [46]. While the human body has mechanisms to protect itself from these ROS, often times, exogenous antioxidants are needed to overcome the effects. Prasad et al. [45] tested the antioxidant activity of a series of aminochalcones. Unfortunately, compound **2** did not show any significant activity with IC_50_ values of 86.02 µg/mL, 192.15 µg/mL, and 64.85 µg/mL against superoxide radical, hydroxyl radical, and ABTS (2,2′-azino-bis(3-ethylbenzothiazoline-6-sulfonic acid) cation radical, respectively. Compound **2** at 50 µg showed inhibition of 17.33% against lipid peroxidation, which is lower than the percentage inhibition of ascorbic acid 20.46% at the same concentration.

Just like compound **1**, compound **2** was also tested for its inhibitory activity against the chlorinating activity of the myeloperoxidase (MPO) enzyme by Zeraik et al. [44]. Compound **2** showed IC_50_ values similar to **1** and was 0.25 ± 0.01 µmol/L for a cell-free, purified MPO system. Thus, the methylation on ring B does not change the potency of aminochalones towards MPO. 

Several 4-aminochalcones have been reported with methoxy side chains on ring B (Figure 5). These compounds have been tested for some biological activities.

The cytotoxic activity for these compounds on different cell lines has been reported by various research groups, which are shown in Table 4.

Compounds **1** and **3a** differ by a methoxy group on ring B. Though the cytotoxic activity between the two does not greatly differ in some cell lines, in others such as CEM and L1210, the IC_50_ value for **1** is lower than that of **3a**. Between the mono methoxylated compounds **3a**–**c**, the values for **3c** are higher than the other two for the Molt 4/C8, CEM, L1210, HeLa, FM3A, and HL-60 cell lines. The cytotoxic activity seems to decrease when the methoxy group is placed on position three on ring B. The most active compound for Molt 4/C8, CEM, L1210, HeLa, and FM3A is **3i**, which is a tri-methoxylated aminochalcone. For the K562 cell line, compound **3d** was the most potent compound, with activity comparable to imatinib and greater than Accutane. Imatinib and Accutane are commercial drugs used to treat different types of cancers. Compounds **3b** and **3f** showed activity higher than both imatinib and Accutane. Suwito et al. [40] showed that the methoxy-4-aminochalcones show better anticancer activity compared with methoxy chalcones and methoxy chalcones with 4-bromo; thus, the presence of 4-amino on ring A is essential for significant antiproliferative activity. The number and position of the methoxy group play a part in the anticancer activity of the compounds. Among known structures that bind to tubulin effectively are combretastatin A-4-5 and colchicine, which seem to have some structural overlap with some of these methoxy-substituted chalcones [41]. 

Apart from these cancer cell lines, the cytotoxic activity of **3a** was also tested against a canine malignant histiocytic cell line (DH82) by Santos et al. [43], with the mean percentage of DH82 cell death equal to 46.6 ± 1.0%, which is quite less when compared with **1** (59.2 ± 4.2%) and **2** (74.7 ± 1.7%), thus showing that the substitution of a strong electron-donating group on ring B decreased cytotoxicity. A similar conclusion was made by Santos et al. [39] when they tested **3a** against estrogen-receptor-positive breast cancer cells MCF-7 and the triple-negative breast cancer (TNBC) cells MDA-MB-23. Compound **3a** did not show any significant activity against the two cell lines.

The antioxidant activities of **3a**, **3h**, and **3i** were tested by Prasad et al. [45]. Compound **3i** had an IC_50_ value of 78.32 ± 1.59 µg/mL against the superoxide radical which was the best among all of the tested chalcones. Compounds **3a** and **3h** had IC_50_ values of 80.09 ± 1.21 and 80.65 ± 1.58 µg/mL, respectively, against the superoxide radical. Compounds **3a**, **3h**, and **3i** all showed a dose-dependent inhibition activity against the hydroxyl radical, with IC_50_ values of 179.01 ± 1.58, 168.63 ± 1.55, and 158.18 ± 1.63 µg/mL, respectively. Against the ABTS radical cation, all three **3a**, **3h**, and **3i** showed remarkable activities which were comparable to the standard drug, ascorbic acid. The IC_50_ values were 56.91 ± 0.68, 52.89 ± 1.31, and 50.06 ± 1.41 µg/mL, respectively, amongst which **3i** showed maximum activity from the tested compounds. For the inhibition of lipid peroxide, **3i** showed the most potency as well (IC_50_ value = 547.18 ± 2.01 µg/mL). The results of this study show that chalcones with electron-releasing groups such as methoxy have greater activity against oxidative species. Between **3a**, **3h**, and **3i**, the scavenging activity increased as the number of methoxy substituents increased on ring B.

Apart from the cytotoxic activities of methoxy-substituted 4-aminochalcomes, the antimicrobial activity of **3a**–**f** has also been tested by Suwito et al. [40] against *Escherichia coli* ATCC 25923, *Staphylococcus aureus* ATCC 259, and *Candida albicans* ATCC 10231 using different concentrations of the compounds, similar to **1**. All of the compounds showed promising antibacterial activity, especially **3d**, which showed the strongest inhibition activity against *Escherichia coli* ATCC 25923 (diameter of inhibition = 10.68 ± 0.16 mm at 500 µg/mL), *Staphylococcus aureus* ATCC 259 (diameter of inhibition = 10.33 ± 0.01 mm at 500 µg/mL), and *Candida albicans* ATCC 10231 (diameter of inhibition = 11.30 ± 0.15 mm at 500 µg/mL). The activity of compound **3d** was comparable to the positive controls sulfamerazine and sulfadiazine. 

**Figure 6:**
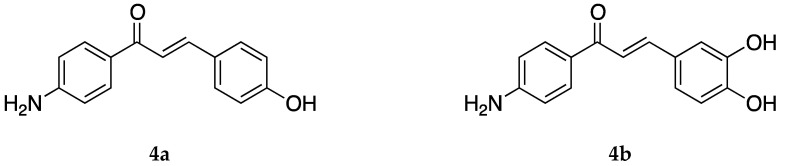
Chemical structures of 4-aminochalcone derivatives with hydroxy substitutions on ring B.

4-Aminochalcones with hydroxy groups on ring B (Figure 6) have also been synthesized and tested for their biological activities. 

Both **4a** and **4b** were tested as α-glucosidase inhibitors, along with several sulfonamide chalcones [47]. α-Glucosidase inhibitors can be used in the treatment of cancer, diabetes, and viral diseases. As glycosidase enzymes are responsible for the processing and synthesis of complex carbohydrates, inhibitors of these molecules can be important tools in glycobiology and can help modulate cellular functions along with biological recognition processes. α-Glucosidases catalyze the release of α-D-glucopyranose from the non-reducing ends of various substrates, inhibitors of which can control the uptake of dietary carbohydrates and, thus, can decrease postprandial hyperglycemia, which may be useful in treating diabetes or obesity. Compounds **4a** and **4b** showed strong inhibitory activities against the three glycosidases that it was tested on. The IC_50_ values for **4a** were 41.0 and 268.9 µM respectively against α-glucosidase from baker’s yeast and α-amylase from *Bacillus licheniformis*. The values for **4b** were 62.1 and 126.8 µM against α-glucosidase from the baker’s yeast and ß-amylase from barley. While this activity did not exceed that of the sulfonamides, it was still considered significant, especially when compared with the respective 3-aminochalcones. 

Kang et al. [48] tested the inhibitory activities of **4a** and **4b** against ß-secretase (BACE1), which is strongly associated with the onset of Alzheimer’s disease (AD) and had IC_50_ values of 48.2 ± 1.2 and 17.7 ± 0.8 µM, respectively. The 3,4-dihydroxy derivate was more potent than the one bearing a 4-hydroxy substituent. While they exhibited inhibitory activity, the respective sulfonamide chalcones were manyfold more potent than these two compounds (and will be discussed in their section). 

Similar to compounds **1** and **2**, **4a** was tested for its inhibitory activity against the chlorinating activity of the myeloperoxidase (MPO) [44]. While both **1** and **2** showed potency towards MPO, the IC_50_ value for **4a** was reported to be greater than 50.00 µM. This indicated that the presence of an easily oxidizable group cancels the inhibitory activity of the aminochalcone. 

Seo et al. [49] also reported the anti-pigmentary effect of **4a**. It had an IC_50_ value of 4.8 µM for tyrosinase enzyme and 48.7 µM against the melanin formation cell. The compound was more potent than kojic acid (IC_50_ = 16.3 µM), a known inhibitor of tyrosinase. 

**Figure 7:**
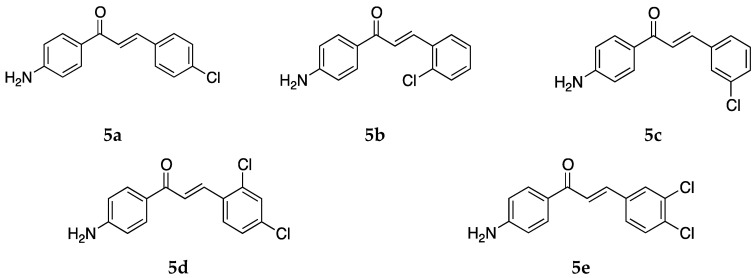
Chemical structures of 4-aminochalcone derivatives with chloro substitutions on ring B.

Several 4-aminochalcones with halogen substitutions, particularly chloro-, fluoro-, and bromo- on ring B have also been reported by various research groups over the years. The 4-aminochalcones with various chlorine substitutions have been reported by four research groups and are shown in Figure 7. The anticancer activity reported for **5a**–**e** by various research groups is shown in Table 5.

The cytotoxicity for Molt 4/C8, CEM, P388, and L1210 was retained when chlorine substitutions were introduced on ring B when compared with **1**. The IC_50_ values for both **5a** and **5e** were found comparable to those of melphalan against some cell lines (IC_50_ values of melphalan: 3.24 ± 0.79, 2.47 ± 0.30, 0.22 ± 0.01, and 2.13 ± 0.03 µM against Molt 4/C8, CEM, P388, and L1210, respectively). Both **5a** and **5e** were more potent to the cell lines as compared to the acylated versions of these aminochalcones [36]. Mai et al. [38] also determined the antiproliferative activities of **5a** and **5d**, and the study revealed that **5a** was more potent than **5d** against most of the cancer cell lines, with its best activity against the CEM-SS cell line. The activities of **5d** were poor against all the cell lines investigated in this study [38]. The 3-chlorophenyl derivative, **5c**, was the most potent compound against the MCF-7 cell line amongst the tested chloro-4-aminochalcones; on the other hand, **5a** showed better activity against the MDA-MB-231 cells. These compounds were more potent than other derivatives with cyano, nitro, and bromo groups [39]. Compound **5a** was the more potent compound amongst all tested by Santos et al. against the canine malignant histiocytic cell line DH82 [43]. The mean percentage cell death of DH82 was 91.0 ± 1.3 at 15.0 µg/mL of the compound. Compound **5a** was subsequently selected for cytotoxic assay against DH82 and MDCK (non-tumorigenic Madin–Darby canine kidney) cells. The IC_50_ values were 31.4 and 57.2 µM, respectively. The selectivity ratio was 1.8. The activity of **5a** against DH82 was greater than etoposide (IC_50_ = 95.5 µM), the reference drug used.

Compounds **5a**, **5b**, and **5d** were also tested for their antioxidant activity by Prasad et al. [45] (Table 6). Amongst the three chloro-4-aminochalcones, **5a**, the 4-chloro-4-aminochalcone was found to be the more potent and showed dose-dependent inhibition against the superoxide radical, the hydroxyl radical, and lipid peroxidation. The activity of **5a** was comparable to the reference ascorbic acid (IC_50_ = 45.42 ± 1.24 µg/mL) for the ABTS cation radical. 

**Figure 8:**
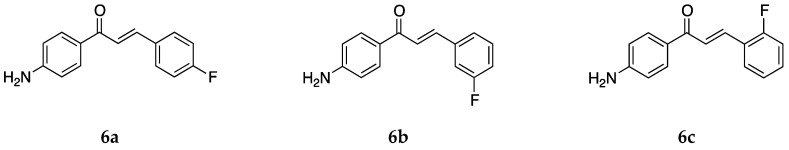
Chemical structures of 4-aminochalcone derivatives with fluoro substitutions on ring B.

Continuing with halogens, research groups also reported the synthesis and activity of fluoro-4-aminochalcones (Figure 8). 

The anticancer activity of the three mono fluoro derivatives was reported by Santos et al. [39] against estrogen-receptor-positive breast cancer cells MCF-7 and the triple-negative breast cancer (TNBC) cells MDA-MB-231. Compound **6a** did not show any significant activity, and the IC_50_ values were not determined. The IC_50_ values for **6b** were >100 and 74.1 ± 1.2 µM for MCF-7 and MDA-MB-231 cancer cells, respectively. Compound **6c** was the more potent against these cells among all of the compounds tested with IC_50_ values 13.2 ± 3.5 and 34.7 ± 5.2 µM, respectively. Compound **6c** induced apoptosis rather than necrosis in both cells. The 3-chloro-4-aminochalcone compound **5c** was also very active against the MCF-7 cell line. A comparison among values for the different compounds tested showed that more electronegative halogens on ring B showed a higher degree of antiproliferative activity against the MCF-7 and MDA-MB-231 cancer cell lines. Electron-donating groups, on the other hand, had a negative effect on bioactivity, as can be seen from the values of compounds **2** and **3a**.

The cytotoxic activity for **6a** was also tested against the canine malignant histiocytic cell line DH82 and non-tumorigenic Madin–Darby canine kidney MDCK cells [43] (IC_50_ = 34.4 and 70.1 µM, respectively). The selectivity ratio was 2.0. Thus, **6a** had a greater selectivity against the DH82 cancer cell line. The three most active compounds in this paper were **2**, **5a**, and **6a**. Compound **2**, having a methyl group, is electron-donating, and the halogen-substituted compounds are electron-withdrawing atoms. Thus, it was concluded that the electronic effect of the substituent did not matter much for the antiproliferative activity against these particular cell lines. The halogen chalcones **5a** and **6a** along with the methyl chalcone **2** are lipophilic and more active than the other compounds tested. Thus, compounds with hydrophobic substitutions on ring B were more potent.

Apart from the anticancer activity, the antioxidant activity for **6a** was also tested by Prasad et al. [45] with IC_50_ values of 79.73 ± 1.58, 175.71 ± 1.61, 61.06 ± 1.78, and 552.18 ± 2.98 µg/mL against the superoxide radical, hydroxyl radical, ABTS cation radical, and lipid peroxidation, respectively. While it was not the most potent compound among all those tested, **6a** showed dose-dependent activity against all of the radicals tested. Its activity was comparable to the standard ascorbic acid for the ABTS cation and superoxide radical. Compound **6a** was also tested for its inhibitory activity against the chlorinating activity of the myeloperoxidase (MPO) enzyme by Zeraik et al. [44] and had an IC_50_ value of 0.25 ± 0.08 µmol/L in a cell-free, purified MPO system. This value is similar to those of **1** and **2**. Similar values for compounds substituted with methyl and fluorine on ring B showed that the main property contributing to MPO inhibition is the presence of both amino and chalcone groups in the compound.

**Figure 9:**
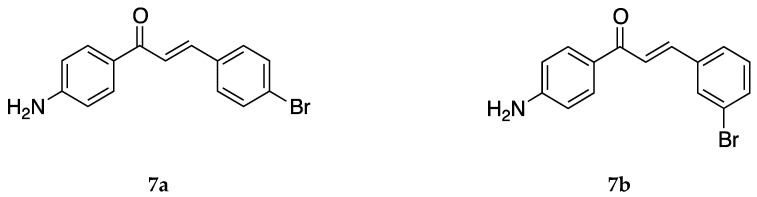
Chemical structures of 4-aminochalcone derivatives with bromo substitutions on ring B.

4-Aminochalcone compounds with bromine substitutions on ring B (Figure 9) have also been reported. 

Both **7a** and **7b** were also tested against the estrogen-receptor-positive breast cancer cells MCF-7 and the triple-negative breast cancer (TNBC) cells MDA-MB-231 [39]. However, these derivatives were not very potent towards the cell lines, especially when compared with compounds substituted with fluorine and chlorine.

The antioxidant activity [45] for **7b** was tested, and the IC_50_ values were reported to be 85.65 ± 1.30, 204.33 ± 1.53, 74.45 ± 0.96, and 600.85 ± 2.38 µg/mL against the superoxide radical, hydroxyl radical, ABTS cation radical, and lipid peroxidation, respectively. While this activity was not terrible, it was not comparable to that of ascorbic acid or other derivatives of 4-aminochalcones.

**Figure 10:**
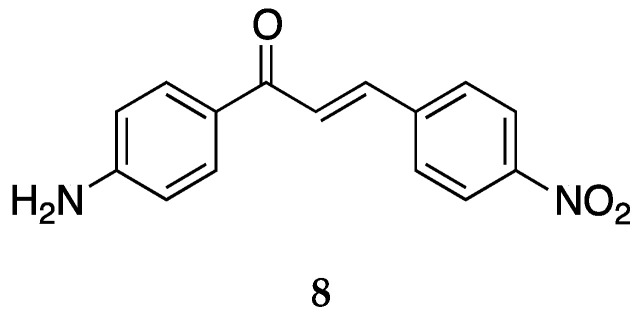
Chemical structures of 4-aminochalcone derivatives with nitro substitution on ring B.

The 4-nitro-4-aminochalcone compound (Figure 10) has also been reported in many articles of some groups.

The cytotoxic activity for **8** was tested against many different cancer cell lines (Table 7). 

The IC_50_ values [36] for the Molt 4/C8, CEM, P388, and L1210 cell lines against compound **8** were better when compared with the simple 4-aminochalcone **1**. The activity for **8** was better than all derivatives, including chloro and methyl, tested for the Molt 4/C8, P388, and L1210 cells. The cell lines tested by Kozlowska et al. [42] showed **8** to be decently potent. Among these cell lines, compound **8** exhibited the highest cytotoxic activity against the HT-29 cell line. Compound **8** exhibited activity better than cisplatin against the HT-29, LoVo, and LoVo/DX cell lines (IC_50_ values of cisplatin: 16.73 ± 0.58, 1.49 ± 0.13, 2.09 ± 0.12, and 2.03 ± 0.17 µg/mL against HT-29, LS180, LoVo, and LoVo/DX, respectively). However, it was also toxic to the normal green monkey fibroblast line COS7 (IC_50_ = 3.10 ± 0.50 µg/mL) used to determine the toxicity of the compounds. Compound **8** was also tested for its anticancer activity against MCF-7 and MDA-MB-231 [39]; however, no significant activity was observed. The mean percentage of DH82 cell death [43] was 52.4 ± 6.7% for **8**.

Kozlowska et al. [42] tested the antimicrobial activity of **8** against two strains of bacteria, *E. coli* ATCC10536 and *S. aureus* DSM799; one strain of yeast, *C. albicans* DSM1386; and three strains of fungi, *A. alternata* CBS1526, *F. linii* KB-F1, and *A. niger* DSM1957. Compound **8** prevented the growth of *E. coli* with minimum inhibitory concentration (MIC) value equal to 0.25 mg/mL, two times stronger than reference oxytetracycline (MIC = 0.5 mg/mL). Compound **8** also prevented the growth of *S. aureus* and *F. linii* (MIC = 0.25 and 1.0 mg/mL).

Prasad et al. [45] also tested the antioxidant activity and scavenging effects for **8**. It had IC_50_ values of 85.31 ± 1.30, 194.22 ± 1.76, 62.32 ± 1.33, and 612.46 ± 2.99 µg/mL, respectively.

**Figure 11:**
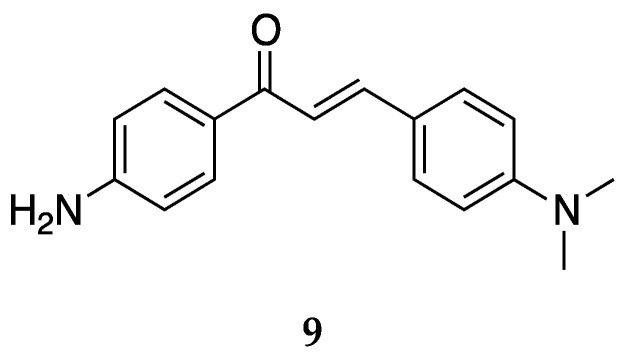
Chemical structures of 4-aminochalcone derivatives with dialkylated amino substitution on ring B.

The antioxidant and anticancer activities were also tested for another very interesting compound with a dialkylated amino group on ring B (Figure 11).

Romagnoli et al. [37] tested **9** against the L1210, FM3A, Molt4, CEM, and HeLa cell lines with IC_50_ values equaling 67 ± 33, 96 ± 32, 9.9 ± 1.0, 20 ± 3, and 28 ± 10 µM. Compound **9** was concluded to show weak antiproliferative activity towards these cell lines.

The potency of **9** against oxidative species, on the other hand, was quite good. The IC_50_ values [45] against the superoxide radical, hydroxyl radical, ABTS cation radical, and lipid peroxidation were 78.87 ± 1.32, 151.48 ± 1.09, 50.36 ± 1.22, and 423.08 ± 2.26 µg/mL, respectively. Compound **9** was one of the most potent compounds tested in this analysis and showed dose-dependent inhibition activities against all assays. It showed the maximum activity, comparable to the standard ascorbic acid against the hydroxyl radical, ABTS radical cation, and inhibition of lipid peroxidation. Compound **8**, having an electron-withdrawing group on ring B, was not very potent against these assays. It was, thus, concluded that electron-releasing substitutions like methoxy and dimethylamino on ring B contribute to the antioxidant activity.

**Figure 12:**
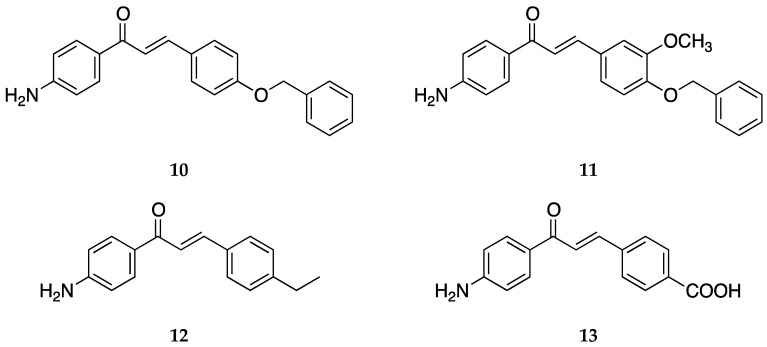
Chemical structures of 4-aminochalcone derivatives.

4-Aminochalcone derivatives with some other substitutions on ring B (Figure 12) were also synthesized and tested by Kozłowska et al. [42].

The anticancer activities of **10**–**13** are shown in Table 8.

The 4-benzyloxy moieties, **10**, **11**, and 4-amino-4-ethylchalcone **12** exhibited inhibition stronger than the reference drug cisplatin (IC_50_ = 16.73 ± 0.58 µg/mL) against the HT-29 cell line. Compound **13** showed the poorest inhibition among all the reported aminochalcones.

The antimicrobial activity for **10**, **11**, and **13** were reported [42] against *E. coli* ATCC10536. The minimal inhibitory concentrations were 0.125, 0.5, and 0.125 mg/mL respectively. Both **10** and **13** showed better inhibition when compared to reference compound oxytetracycline (MIC = 0.5 mg/mL against *E. coli* ATCC10536).

**Figure 13:**
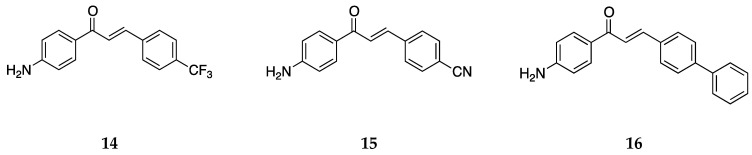
Chemical structures of 4-aminochalcone derivatives.

The 4-aminochalcones synthesized by Santos et al. [39] included those shown in Figure 13.

These compounds were tested against the estrogen-receptor-positive breast cancer cells MCF-7 and the triple-negative breast cancer (TNBC) cells MDA-MB-231. Compounds **14** and **15** did not show very significant antiproliferative activity as their percentage of metabolically viable cells (%MVC) was greater than 98% against both cell lines. The IC_50_ values for **16** were 22.7 ± 6.0 and >100 µM against MCF-7 and MDA-MB-231, respectively. Compound **16** had a %MVC value of 44.2 ± 3.3 against MCF-7.

**Figure 14:**
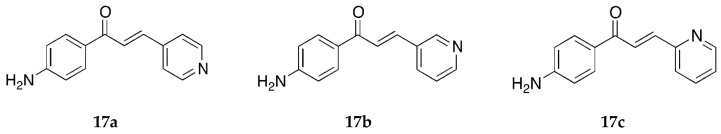
Chemical structures of 4-aminochalcone derivates containing pyridine rings.

Some derivatives containing pyridine rings have also been reported (Figure 14).

Compounds **17a** and **17b** were synthesized by Santos et al. [39] using the Claisen-Schmidt aldol condensation method, and their cytotoxic activity was tested against the MCF-7 and MDA-MB-231 cancer cell lines as well. Compound **17b** showed significant antiproliferative activity against the MCF-7 cell line with an IC_50_ value of 15.7 ± 5.9 µM. Compound **17b** was also potent against the MDA-MB-231 cell line (IC_50_ = 33.9 ± 7.1 µM). Apart from the 4-fluorophenyl derivative **6c**, **17b** was the only compound among those reported in the manuscript which showed significant potency against both cell lines. Compound **17b** induced apoptosis rather than necrosis in both cells and upregulated the p53 expression in the MCF-7 cell line. Compound **17a** was active against the MCF-7 and showed a percentage of metabolically viable cells (%MVC) value of 61.3 ± 3.4. Its IC_50_ value was not determined.

The antioxidant activity for **17a**, **17b**, and **17c** was tested by Prasad et al. [45] Many 4-aminochalcones reported in this manuscript have already been discussed earlier, with some of them showing significant antioxidant activity comparable to the reference ascorbic acid. Compounds **17a**, **17b**, and **17c**, however, did not show very significant activity against the superoxide, hydroxyl, ABTS cation radicals, and lipid peroxidation (Table 9). The IC_50_ values for these compounds were not comparable to ascorbic acid or the more potent compounds tested by the group.

**Figure 15:**
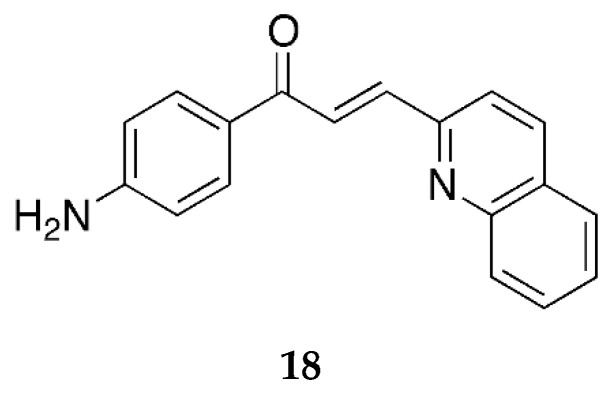
Chemical structures of the 4-aminochalcone derivate with a quinoline ring.

**Figure 16:**
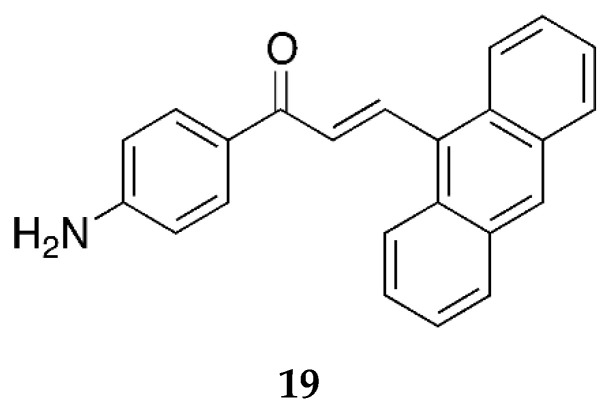
Chemical structures of the 4-aminochalcone derivate with an anthracene ring.

Prasad et al. [45] have also reported the 2-quinolinyl 4-aminochalcone derivative (Figure 15) and aminochalcone containing anthracene ring (Figure 16).

Compound **18** showed values similar to the pyridinyl derivatives (**17a**, **17b**, and **17c**). Its IC_50_ values against the superoxide radical, hydroxyl radical, ABTS cation radical, and lipid peroxidation were 88.87 ± 1.78, 197.87 ± 1.48, 77.89 ± 2.52, and 656.54 ± 1.75 µg/mL, respectively.

Compound **19** also did not show very significant antioxidant activity with IC_50_ values 89.25 ± 1.99, 198.22 ± 1.69, 76.32 ± 2.66, and 652.25 ± 1.44 µg/mL against the superoxide radical, hydroxyl radical, ABTS cation radical, and lipid peroxidation, respectively. Lack of significant activity might be due to the absence of any strong electron-releasing group such as methoxy or dimethylamino on ring B, as derivatives containing these substitutions were more potent.

**Figure 17:**
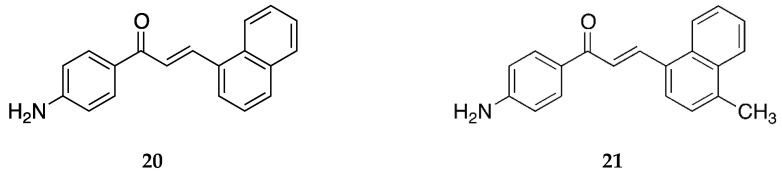
Chemical structures of 4-aminochalcone derivates with a naphthalene ring.

4-amino-1-naphthylchalcone **20** and its methylated derivative **21** (Figure 17) were synthesized by Seba et al. [50], and their activity against osteosarcoma cells was reported.

Osteosarcoma is the most recurrent malignant bone tumor. This tumor shows a high propensity for local invasion and distant metastasis. Due to this, the long-term survival rate of osteosarcoma patients with metastasis is decreased by 20–30% [51]. Seba et al. [50] studied the inhibitory effects of **20** and **21** against osteosarcoma migration. They found that, while these compounds neither induce apoptosis in osteosarcoma cells can suppress tumor cell lines U2OS and SOAS, they showed inhibitory effect against migration and invasion of osteosarcoma cells. This effect was more potent in p53-expressing cells, namely, U2OS. They increased p53 expression and reduced the activity and expression of two matrix metalloproteinases: MMP-2 and MMP-9. These compounds also downregulated the epithelial–mesenchymal transition (EMT). The migration and invasion of tumor cells towards the vascular/lymphatic system involves MMPs, which ultimately triggers metastasis. Tumor cells undergo various changes during their EMT, which relate to amplified migration, invasion, and metastasis of these cells. Thus, the targets of compounds **20** and **21** are important in the process of migration and invasion of osteosarcoma cells.

Compound **20** was also reported by Santos et al. [39]. It showed significant antiproliferative activity against the estrogen-receptor-positive breast cancer cells MCF-7 (IC_50_ = 14.3 ± 2.9 µM). It was inactive against the triple-negative breast cancer (TNBC) cells MDA-MB-231 with an IC_50_ value of >100 µM.

Santos et al. [43] reported the cytotoxic activity of **20** against the canine malignant histiocytic cell line DH82 as the mean percentage of DH82 cell death, which was 40.3 ± 0.6.

**Figure 18:**
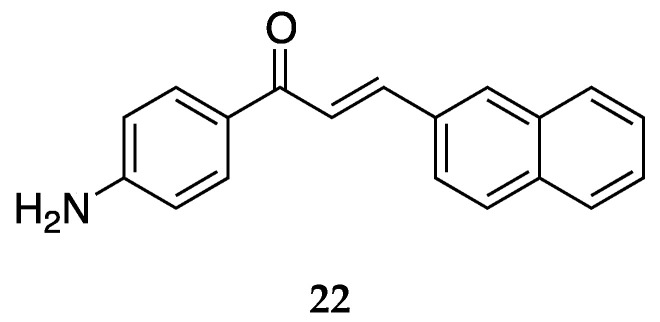
Chemical structure of 4-aminochalcone derivate with a naphthalene ring.

A 2-napthyl derivative of 4-aminochalcone, **22**, was also synthesized and tested by Santos et al. [43] (Figure 18). It showed a mean percentage of DH82 cell death as 40.4 ± 3.7.

Both chalcones with naphthyl rings showed poor activity against the DH82 cell line.

**Figure 19:**
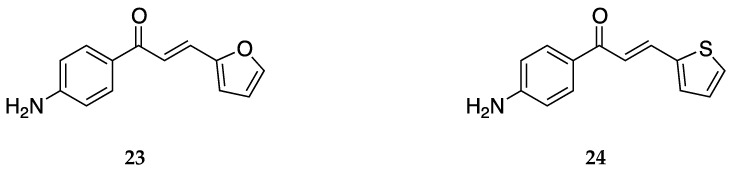
Chemical structures of 4-aminochalcone derivates with heterocycles.

4-aminochalcones with heterocycles instead of the phenyl ring B have also been synthesized (Figure 19).

The antiproliferative activity of compounds **23** and **24** was reported by Santos et al. [43] against the canine malignant histiocytic cell line DH82. The mean percentages of DH82 cell death values were 18.8 ± 4.0 and 43.6 ± 3.7, respectively. Thus, neither compound showed significant activity against the cell line and **23** had the poorest activity amongst all of the compounds reported in the manuscript. In addition to this, **23** was also tested by Santos et al. [39] against the estrogen-receptor-positive breast cancer cells MCF-7 and the triple-negative breast cancer (TNBC) cells MDA-MB-231. It showed %MVC values of 104.5 ± 3.6 and 100.1 ± 7.1 against the cell lines, respectively. Thus, it can be concluded that these compounds do not show significant cytotoxic activity.

**Figure 20:**
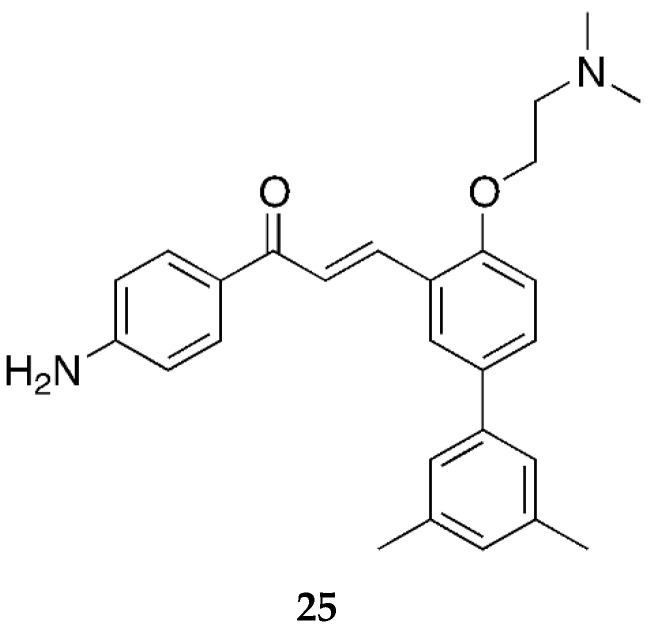
Chemical structures of the 4-aminochalcone derivate.

Moving towards the end of our discussion for 4-aminochalcones, compound **25** (Figure 20) was synthesized by Nielsen et al. [52] and tested for its antibiotic activity along with many other chalcones.

Compound **25** was tested against the *S. aureus* ATCC33591 (resistant to methicillin), *E. faecium* 17501 (vancomycin-resistant clinical isolate), and *E. faecium* ATCC29212 bacterial cell lines and showed MIC values of 10, 10, and 20 µM, respectively. As the values indicate, **25** had no significant effect on the activity. However, it showed some influence on the bacterial membrane. This is likely due to the protonation of the dimethylamino group on ring B.

#### 2.1.2. 2-Aminochalcone and Derivatives

**Figure 21:**
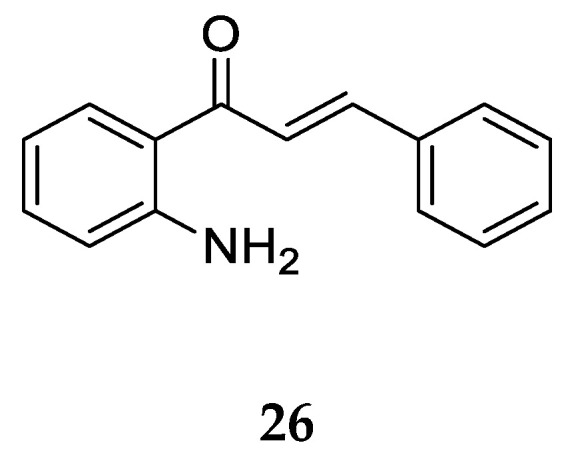
Chemical structure of 2-aminochalcone.

Aminochalcone **26** (Figure 21) with a -NH2 substitution at position 2 on ring A is the simplest among 2-aminochalcones. Kozlowska et al. [42] obtained it through a modified protocol for a base-catalyzed Claisen-Schmidt condensation described by Amir et al. [53] using 2-aminoacetophenone and benzaldehyde.

Following synthesis, its anticancer activity was tested against various cancer cell lines using green monkey fibroblast COS7, selected as the normal cell line to determine toxicity (Table 10) [42]. Besides, Mai et al. [38] also synthesized and tested the antiproliferative effect of **26** using the methyl thiazolyl tetrazolium (MTT) assay on ten different cancer cell lines, namely, human estrogen receptor-positive breast cancer cells (MCF7), human estrogen receptor-negative breast cancer cells (MDA-MB-231), human cervical cancer cells (HeLa), human ovarian cancer cells (Caov-3), human lung cancer cells (A549), human liver cancer cells, (HepG2), human colorectal cancer cells (HT-29), human nasopharyngeal cancer cells (CNE-1), human T-lymphoblastoid leukaemia cells (CEM-SS), human erythromyeloblastoid leukaemia cells (K562), and normal human embryonic kidney (HEK-293) cells.

Aminochalcone **26** was the strongest inhibitor of HT-29 cell line proliferation, as indicated by the values in Table 10. It exhibits activity almost 12 folds higher than that of reference compound cisplatin (IC_50_ = 16.73 ± 0.58 µg/mL) and 4 folds lower than that of doxorubicin (IC_50_ = 0.33 ± 0.02 µg/mL). Its cytotoxicity against the COS7 normal cell line indicated its poor selectivity against cancer cells [42]. Additionally, in comparison to the 4-aminochalcone discussed earlier, the IC_50_ values of **26** are lower for the cell lines in Table 1. This increase in potency is caused just by the change in position of the amine group from 4 to 2 on ring A.

Mai et al. [38] reported that the selectivity ratio (IC_50_ HEK-293/IC_50_ HT-29) of **26** (ratio > 22.78) fulfilled the threshold limit (ratio ≥ 5); it was one of the promising anticancer agents. They also observed that, among all the chalcones tested, **26** showed the most potent antiproliferative effect against HT-29 cells, indicating that the -NH2 group on ring A plays an important role. The anti-proliferative effects exhibited by the aminochalcone **26** were due to apoptosis rather than necrosis and were dose-dependent.

Kozlowska et al. [42] investigated the antimicrobial activity of **26** against bacterial strain *Staphylococcus aureus* DSM799 and strains of fungi, *Alternaria alternata* CBS1526 and *Aspergillus niger* DSM1957. The minimum inhibitory concentration (MIC) values were evaluated and compared with reference substances, such as oxytetracycline (against bacteria) and nystatin (against fungi). The MIC value of **26** was 1.0 mg/mL for *A. alternata*. Complete growth inhibition was observed in cases of *S. aureus* (MIC = 0.25 mg/mL) and particularly *A. niger* (MIC = 0.125 mg/mL), which was two times more effective than nystatin.

Furthermore, the antimicrobial activity of **26** against several bacteria was tested and reported by Ruanwas et al. [54] Against all the gram-positive bacteria—*Bacillus subtilis*, *Staphylococcus aureus*, *Enterococcus faecalis* TISTR 459, methicillin-resistant *Staphylococcus aureus* (MRSA) ATCC 43300, and vancomycin-resistant *Enterococcus faecalis* (VRE) ATCC 51299—and gram-negative bacteria—*Salmonella typhi*, *Shigella sonei*, and *Pseudomonas aeruginosa*—compound **26** showed MIC values >300 µg/mL, which were significantly greater than the vancomycin standard (MIC < 2.34 µg/mL). These results are similar to the antimicrobial activity of 4-aminochalcone, which differs only by the position of the -NH2 group on ring A. This suggests that a change in position from 4 to 2 does not cause a big difference in antimicrobial activity. The same group also tested the antioxidant activity of compound **26** via a DPPH (2,2-diphenyl-1-picrylhydrazyl radical) antioxidant assay, which was later compared to the standard ascorbic acid. Aminochalcone **26** showed a percentage inhibition of 18.7%, which was the highest among the aminochalcones tested for free radical scavenging. However, it was notably less than the ascorbic acid standard, which exhibits 92.1% inhibition [54].

A variety of 2-aminochalcone derivatives with substitutions on ring B have been synthesized and reported for biological activity by Santos et al. [43].

**Figure 22:**
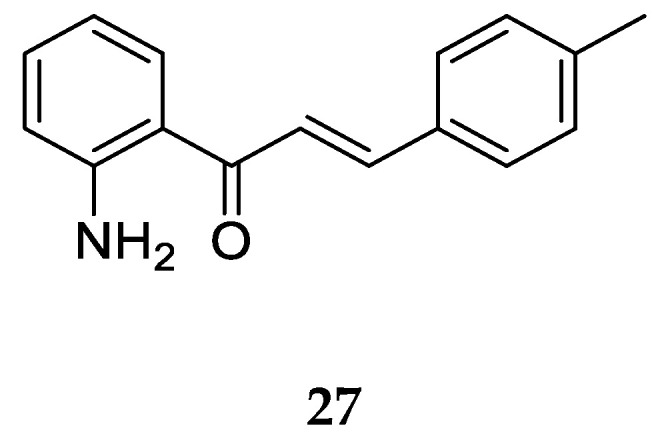
Chemical structure of 2-aminochalcone derivatives with methylation on ring B.

Aminochalcone **27** (Figure 22) is an analog of the methylated 4-aminochalcone discussed earlier. It was synthesized and evaluated for anticancer activity against the canine malignant histiocytic cell line (DH82). The mean percentage of cell death was found to be 88.9 ± 1.5 at 15.0 µg/mL of the compound. Compound **27** was one of the three most active compounds tested due to its selective toxicity and apoptosis induction in DH82 cells compared to a nontumorigenic canine cell line (Madin-Darby canine kidney cells, MDCK). Compound **27** also showed a slight increase in cytotoxicity in comparison to its 4-aminochalcone analog. The compound largely owes its activity to the presence of a hydrophobic group on ring B. Similarly, against DH82 and MDCK cells, a nontumorigenic cell line, the IC_50_ values of **27** were 38.2 µM and 23.3 µM, reflecting approximately three times more potency against DH82 than the reference antitumor drug etoposide (IC_50_ = 95.5 µM). It was also observed that **27** induces cell death through apoptosis rather than necrosis in canine cells. Therefore, it can be deduced that compound **27** possesses a remarkable potential for anticancer activity against the tested cell lines.

**Figure 23:**
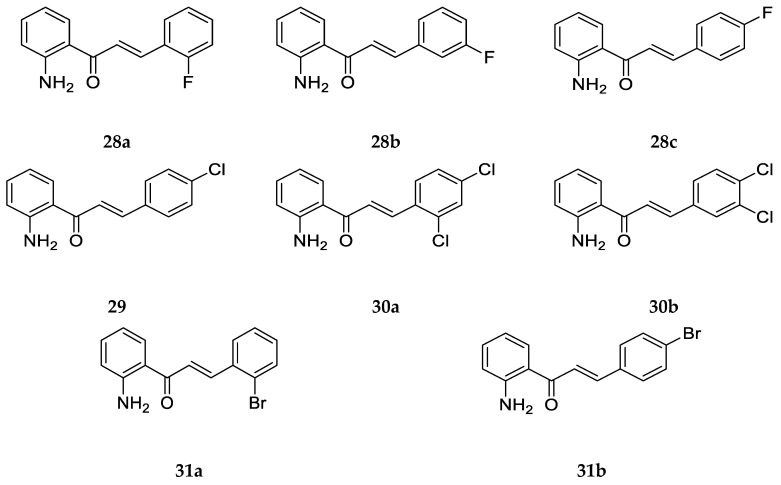
Chemical structures of derivatives of 2-aminochalcone with halogen substitution on ring B.

Santos et al. [43] and Zhang et al. [55] also prepared and investigated the biological activity of a variety of 2-aminochalcones derivatives with halogens substituted on ring B (Figure 23).

The anticancer activity of **28c**, **29**, and **31b** against canine malignant histiocytic cell line (DH82) was determined as the mean percentage of DH82 cell death ± SEM at 15.0 mg/mL of the compound. The activities of **28c**, **29**, and **31b** were 76.6 ± 0.1, 83.2 ± 5.7, and 87.0 ± 7.5, respectively. These halogenated 2-aminochalcones exhibited cytotoxic activity similar to that of their halogenated 4-aminochalcone analogs [43].

Like **26**, compound **29** was also prepared and tested against ten cancer cell lines for antiproliferative activity by Mai et al. [38]. The IC_50_ values of **29** are summarized in Table 11.

From the values in Table 11, we can infer that the activity of **29** is the best against the HT-29 cell line. It is seen from the comparison of the activity of **29** with **26** that compound **26** is more potent against most cell lines including A549, HEPG2, CNE-1, K562, and CEM-SS whereas **29** has a higher antiproliferative activity against the Caov-3 cell line and HT-29 shows similar values for both **26** and **29**. Therefore, the presence of -NH2 on ring A position 2 has a more significant contribution to the anticancer activity as opposed to the substitutions on ring B.

Additionally, Zhang et al. [55] tested the antifungal activity of compounds **28a**–**c**, **30a**–**b**, and **31a** against a single strain *Trichophyton rubrum*. Their MIC80 values were found to be 1 µg/mL, 0.5 µg/mL, 1 µg/mL, >64 µg/mL, >64 µg/mL, and 1 µg/mL, respectively. Compound **28b** was the most potent and **30a–b** was the least potent among the compounds, and most of the MIC80 values recorded were greater than that of the reference drug Fluconazole (MIC80 = 0.25 µg/mL).

**Figure 24:**
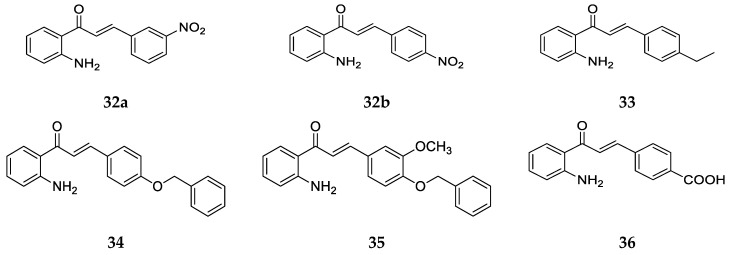
Chemical structures of derivatives of 2-aminochalcone with substitution on ring B.

More derivatives of 2-aminochalcones, some of which were novel with different substituents on ring B (Figure 24), were synthesized by Kozlowska et al. [42], Mai et al. [38], and Zhang at al. [55].

The biological activity of the compounds was investigated against cancer cell lines and different microbes.

Table 12 shows the IC_50_ values of compounds **32b**, **33**, **35**, and **36** against cancer cell lines as investigated by Kozlowska et al. [42] and Mai et al. [38].

Compound **33** showed IC_50_ values lower than **36** but higher than **32b** for the cancer cell lines tested. Similar to the anticancer effect of compound **27** with the methyl group on ring B, the higher potency of **33** compared to **36** is also due to the hydrophobicity of the ethyl group. Also, the cytotoxicity of **33** against the COS7 normal cell line is lower than that of **26**, the simple 2-aminochalcone which indicates the positive effect of ethyl substituent on ring B. Besides, the anticancer activity of **36** is observed to be the weakest and it was not tested against some cancer cell lines. The activity of **32b** is impressive in comparison to the others tested. This compound was made and investigated for bioactivity by both groups [38,42] as shown in Table 12 as IC_50_ values. Kozlowska and colleagues [42] found compound **32b** to be slightly more potent against the common cell line HT-29 but still less active than compound **26**. When compared with **33** and **36**, **32b** was more potent against all four cancer cell lines while also exhibiting toxicity against the COS7 normal cell line. Compound **35** showed moderate anticancer activity, with lower toxicity against the COS7 normal cell line, but there was no notable feature observed in the overall activity of **35** [42]. Mai et al. [38] reported that, since **32b** exhibited good anticancer activity against most of the cell lines tested and the selectivity ratio (IC_50_ HEK-293/IC_50_ HT-29) of **32b** (ratio = 6.77) was greater than the threshold value (ratio ≥ 5), it was one of the promising anticancer agents like **26**.

In addition to anticancer activity, Kozlowska et al. [42] also determined the antimicrobial activity in terms of minimum inhibitory concentrations (MIC) of compounds **33**, **34**, **36**, and **32b** against bacterial and fungal strains. Compound **32b** was tested against *Escherichia coli* ATCC10536 with (MIC = 0.25 mg/mL), *Candida albicans* DSM1386, and *F. linii* KB-F1, with MIC values = 0.5 mg/mL each. These values are almost equivalent to the standard drugs against which they were compared, and **32b** showed MIC values lower than its 4-aminochalcone analog, thus revealing its antimicrobial potential and the significant impact of the position of the NH2 group (ring A) on the biological activity of aminochalcones. Compound **33** showed MIC value = 0.5 mg/mL when tested against *Escherichia coli* ATCC10536, which was equal to the standard oxytetracycline. Compound **34** was only tested for antimicrobial activity and displayed MIC values of 0.25 mg/mL, 0.25 mg/mL, and 0.5 mg/mL against *Escherichia coli* ATCC10536, *Staphylococcus aureus* DSM799, and *Candida albicans* DSM1386, respectively, and a better activity than the standard oxytetracycline (MIC = 0.5 mg/mL) for *E. coli*. The MIC values of **36** were 0.125 mg/mL against *Escherichia coli* ATCC10536, *Staphylococcus aureus* DSM799, *Candida albicans* DSM1386, and *Aspergillus niger* DSM1957 and 0.5 mg/mL against *Alternaria alternata* CBS1526. Compound **36** was found to be the best inhibitor of *C. albicans* among the compounds tested, with four times higher activity than the standard cycloheximide. For the fungal strains, **36** showed a lower MIC value and thus higher activity than the standard drug nystatin, overall exhibiting a remarkable antimicrobial activity. Finally, the minimal inhibitory concentration of compound **35** was determined for *Escherichia coli* ATCC10536, *Staphylococcus aureus* DSM799, and *F. linii* KB-F1 and was found to be 0.5 mg/mL for all three strains. Overall, it displayed an equal or poorer performance in comparison to the respective standard drugs and in comparison to other chalcones tested. The MIC values for **35** were higher than its structural analog **34**, meaning that the addition of the methoxy group on ring B along with the benzyloxy group does not improve bioactivity but decreases it.

Lastly, values of the minimum inhibitory concentration required to inhibit 80% of cells (MIC80) for compounds **32a**–**b** were determined by Zhang et al. [55]. The compounds were found to be potent against *Trichophyton rubrum*, with MIC80 values of 0.5 µg/mL each. This value was greater than the standard reference, but **32a**–**b** was among the three most active antifungal agents compared to other chalcones tested.

**Figure 25:**
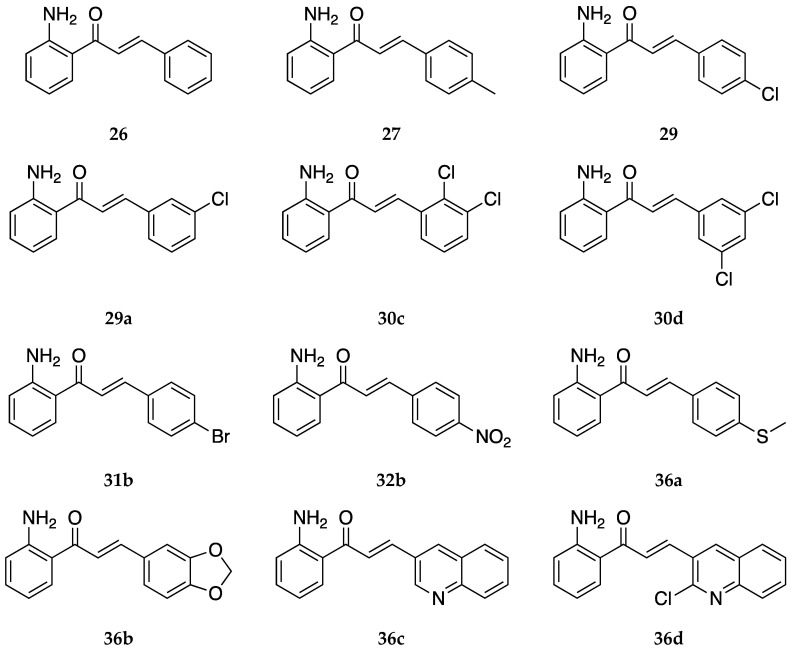
Chemical structures of derivatives of 2-aminochalcone with substitution on ring B.

Sakata et al. [56] synthesized a series of 2-aminochalcones (Figure 25) and tested their activity against the neurodegenerative Alzheimer’s disease markers, namely, acetylcholinesterase (AChE), β-secretase (BACE-1), and the amyloid-β (Aβ). As discussed earlier, some of these compounds have been also reported by other groups.

The activities of these compounds against AChE and BACE-1 are shown in Table 13.

Tacrine was used a positive control for AChE inhibition, and quercetine was used for BACE-1. Against AChE, all compounds tested showed some inhibition activity, with compounds **27**, **30c**, **36a**, **36b**, and **36c** showing IC_50_ values lower than 10 µM. The 2′,3′-dichloro substituted aminochalcone **30c** exhibited the highest potency, with IC_50_ equal to 0.08 µM. These compounds were docked into the active site of AChE, and it was concluded that the high potency for some of these compounds was due to the hydrophobic features of the analogues as the aromatic ring helped in the adoption of a proper conformation at the ligand cleft. The IC_50_ values for BACE-1 inhibition were obtained only for compounds that showed decent percentage inhibition values. Against BACE-1, compound **30c** again showed the highest potency (IC_50_ = 2.71 µM). Some chalcones without the amino group having structural similarities with compounds in Figure 25 were tested against BACE-1. These chalcones did not inhibit the enzyme, thus showing the importance of the amino group on ring A. The molecular docking studies performed showed that the amino group on ring A interacts with the aspartic acid residues of the catalytic diade of BACE-1. The inhibitory effect on the Aβ amyloid aggregation was determined using thioflavin-T (ThT), which binds to the β-amyloid fibrils. This interaction increases the emission of ThT fluorescence; thus, the presence of an inhibitor will decrease the fluorescence. Aminochalcones **29a**, **29**, **30c**, and **36b** showed a good effect against Aβ fibrils formation and reduced the ThT fluorescence emission by 37, 52, 33, and 29%, respectively. The most potent compound was **29**, as it was comparable to the reference compound curcumin (inhibition of fibril formation = 56%). Moreover, the cytotoxicity for these 2-aminochalcones was tested against the human glioblastoma M059J, mouse fibroblast Vero, and kidney epithelial 3 T3 cell lines. While 3 T3 was more sensitive to the chalcones, the overall IC_50_ values obtained were quite high, showing that they were not potentially cytotoxic. Furthermore, it was determined that these compounds have low water solubility, which might be due to the presence of crystal packing. In conclusion, these compounds are promising leads against AD especially if the hydrosolubility is improved.

**Figure 26:**
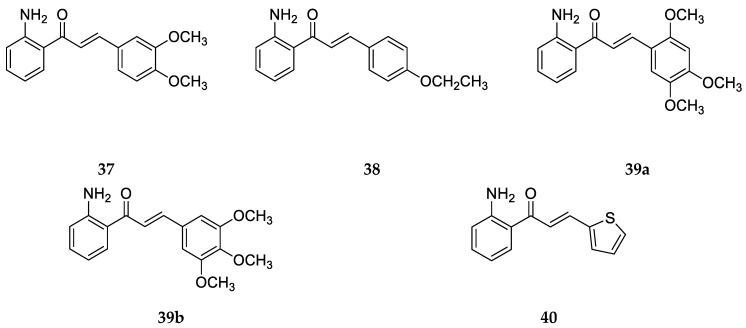
Chemical structures of derivatives of 2-aminochalcone with substitution on ring B.

Moreover, 2-aminochalcone derivatives with methoxy and other substitutions on ring B (Figure 26) were synthesized by Zhang et al. [55], who tested the antifungal activity, and Ruanwas et al. [54], who screened them for antioxidant and antibacterial activity.

Aminochalcone **37** was tested for antifungal activity against *Trichophyton rubrum*, and its minimum inhibitory concentration needed to inhibit 80% of cells (MIC_80_) was determined to be greater than 64 µg/mL, which was higher than most of the chalcones tested and overall demonstrated that **37** was not a good antifungal agent [55].

2-Aminochalcones **38**, **39**, and **40** also showed poor antibacterial activity against the gram-positive bacteria—*Bacillus subtilis*, *Staphylococcus aureus*, *Enterococcus faecalis* TISTR 459, methicillin-resistant *Staphylococcus aureus* (MRSA) ATCC 43300, and vancomycin-resistant *Enterococcus faecalis* (VRE) ATCC 51299—and the gram-negative bacteria—*Salmonella typhi*, *Shigella sonei*, and *Pseudomonas aeruginosa* [54]. The MIC values of these compounds for all bacterial strains were ≥300 μg/mL, like compound **26**. The low MIC value of the vancomycin standard (<2.34 μg/mL) demonstrates the inadequate activity of the compounds against the strains tested. The antioxidant activities of compounds **38**–**40** were tested through a DPPH radical scavenging method and were compared to ascorbic acid as a standard reference. The range of the percentage inhibition of free radical scavenging activity of **38**–**40** was 0.41% to 3.27%, with **38** showing the highest and **39b** showing the lowest activity among these compounds. These activities are lower than that of **26** (18.71%) found during the same investigation and significantly weaker than the ascorbic acid reference (92.06% inhibition). The results suggest that the general antioxidant activity by the α,β-unsaturated carbonyl group was reduced due to the substituents on ring B in **38**–**40** [54].

**Figure 27:**
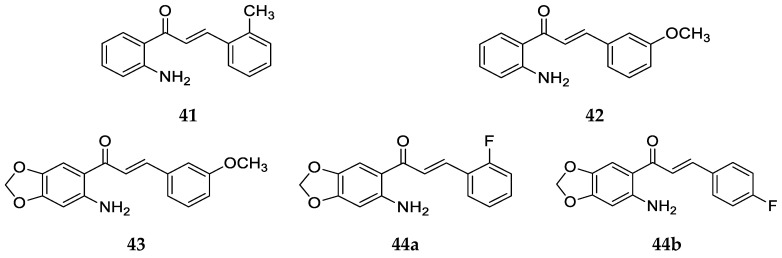
Chemical structures of derivatives of 2-aminochalcone with substitution on rings A and B.

Adding diversity to the plethora of 2-aminochalcones, we will discuss Xia et al.’s [57] prepared derivatives of 2-aminochalcone with substituents on both rings ranging from simple methyl to methylenedioxy substitution (Figure 27).

These compounds (**41**–**43, 44a**, and **44b**) were synthesized by a base-catalyzed condensation of 2-amino acetophenones and aldehydes with appropriate substitutions. Then, they were assayed for cytotoxicity in vitro against nine human tumor cell lines [57], namely, epidermoid carcinoma of the nasopharynx (KB), P-gp-expressing epidermoid carcinoma of the masopharynx (KB-VIN), osteosarcoma (Hos), melanoma (SKMEL-2), ileocecal carcinoma (HCT-8), breast cancer (MCF-7), lung carcinoma (A-549), glioblastoma (U87-MG), and ovarian cancer (1A9) cell lines. The ED_50_ values of the compounds are listed in Table 14.

Compounds **41**–**44b** showed significant cytotoxic activity (ED_50_ ≤ 4.0 mg/mL) and pronounced selectivity against the KB, KBVIN, and 1A9 cell lines. Compound **42** was more potent than **41** against these cancer cell lines, with notably lower values for the compound concentration, affording a 50% reduction in cell number. However, compound **43**, which is a structural analog of **42** and contains a methylenedioxy moiety on ring A (positions 4 and 5), was the most active in this study, with the lowest ED_50_ values, making it a potential lead for antitumor agents. Among **44a** and **44b**, only differing in the position of fluorine on ring B, a relatively better activity was shown by **44a** and the highest ED_50_ values; thus, the least potency was displayed by **44b** for most of the cancer cell lines. Also, when compared to a similar chalcone only without a –NH_2_ group at position 2 on ring A, compound **44a** was about 40-fold more active, demonstrating the importance of the amino group for biological activity. It was concluded that the introduction of a methylenedioxy moiety on ring A led to enhanced cytotoxic activity compared with the A-ring-unsubstituted compound **42** and that the presence of a methoxy group at position 3 on ring B was beneficial for increased cytotoxicity [57].

**Figure 28:**
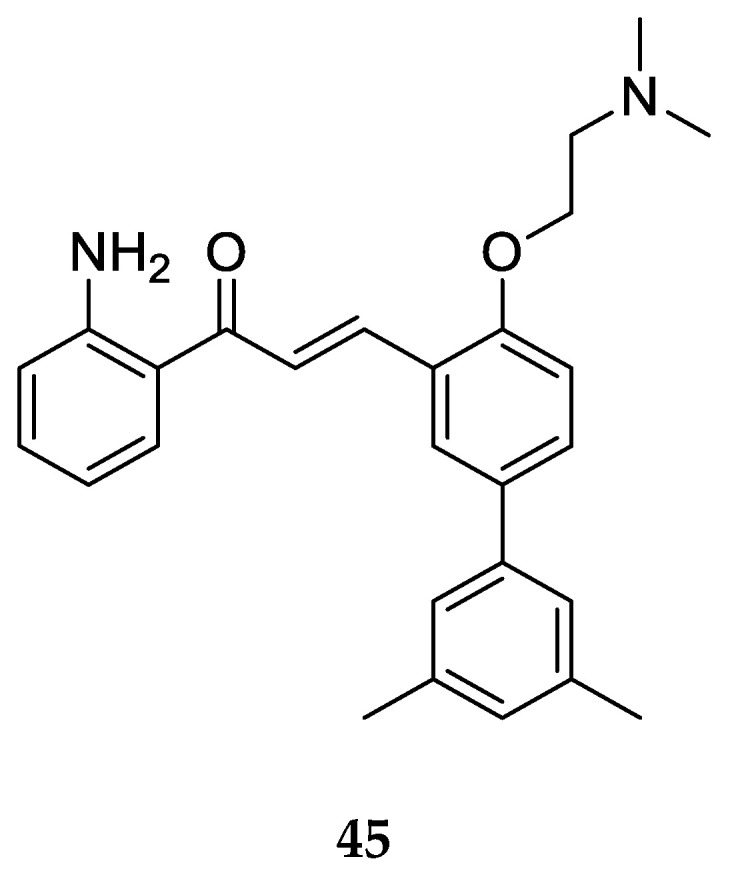
Chemical structure of the 2-aminochalcone derivative with substitution on ring B.

The 2-aminochalcone derivative with larger substitutions on ring B was prepared and tested for antibacterial activity by Nielsen et al. [52] (Figure 28).

Compound **45** was tested against bacterial strains *Staphylococcus aureus* ATCC33591 (resistant to methicillin), *Enterococcus faecium* 17501 (vancomycin-resistant clinical isolate), and *Enterococcus faecium* ATCC29212, with MIC values 5 µM, 5 µM, and 10 µM, respectively. Compound **45** and similar 2-aminochalcones with ring B substitutions were found to be more selective for the bacterial membrane. The MIC values also illustrated better antibacterial activity of **45** as compared to its 3-aminochalcone analog (MIC = 10 µM each) and 4-aminochalcone analog **25** (MIC = 10 µM and 20 µM), indicating that the position of the -NH_2_ group on ring A matters for antibacterial activity.

**Figure 29:**
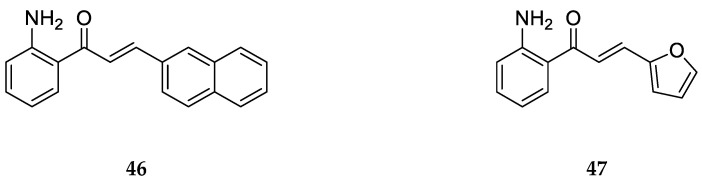
Chemical structures of 2-aminochalcone derivatives with substitution on ring B.

Similarly, 2-aminochalcones **46** and **47** are derivatives with substitutions on ring B synthesized and screened for antifungal activity by Zhang et al. [55] (Figure 29).

Compounds **46** and **47** displayed no significant antifungal activity against *Trichophyton rubrum* with MIC_80_ values >64 µg/mL. This shows that the substitutions on ring B of these compounds are not suitable to inhibit fungal activity, and this might be due to the bulk of the groups as the 2-aminochalcones with smaller substituents showed potent antifungal activity [55].

#### 2.1.3. 3-Aminochalcone and Derivatives

**Figure 30:**
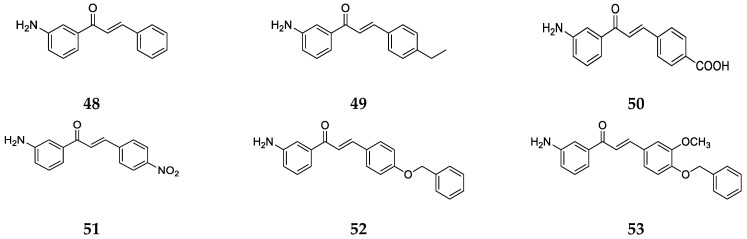
Chemical structures of 3-aminochalcone derivatives with substitution on ring B.

Approaching the last class of aminochalcones that has a -NH_2_ substitution on ring A, we introduce 3-aminochalcones and its derivatives with an amine group on position 3 of ring A. In addition to the variety of 4-aminochalcones and 2-aminochalcones, a diverse group of 3-aminochalcones including the simplest one was also synthesized by Kozlowska et al. [42] (Figure 30).

The antiproliferative potential was evaluated on four different human colon cancer cell lines, HT-29, LS180, LoVo, and LoVo/DX, using the Sulforhodamine B (SRB) assay followed by comparison with normal green monkey kidney fibroblast COS7 cells. The activities are summarized in Table 15 as IC_50_ values.

The best activity was observed for compound **48** against the HT-29 cell line and for compound **51** against the LoVo and LoVo/DX cell lines. However, all compounds were cytotoxic against COS7, demonstrating poor selectivity. In general, compounds with ethyl, nitro, and benzyloxy groups at position 4 on ring B represented a group of inhibitors stronger than cisplatin against the HT-29 cell line. For the bioactivity of compound **50** and its analogs **13** and **36,** the role of the position of the amine group played a significant role. The strongest inhibition was observed for 2-aminochalcone **36** followed by 3-aminochalcone **50** and then at last 4-aminochalcone **13**.

Moreover, the antimicrobial activity of compounds **48**–**53** tested by Kozlowska et al. [42] is displayed in terms of minimal inhibitory concentration (MIC) values in Table 16.

The growth of *E. coli* ATCC10536 was prevented to a decent degree by most of the tested aminochalcones with the lowest MIC value shown by **52**. Likewise, complete growth inhibition was observed for all these compounds in the case of *S. aureus* DSM799. Compounds **48**, **51**, and **53** successfully prevented the growth of *C. albicans* DSM1386, and *A. alternata* CBS1526 were tested. Compound **51** produced better results than its structural analog 4-aminochalcone **8** against *F. linii* KB-F1. The overall antimicrobial activity of these compounds is propitious and reveal the significant impact of the position of an amine group on ring A on biological activity [42].

Moreover, the simple 3-aminochalcone **48** (Figure 30) along with the aminochalcone analogues **1** and **26** were also synthesized by Trein et al. [58] and tested against the parasitic protozoan *Trichomonas vaginalis*. This flagellate protozoan is the etiological agent of trichomoniasis, a very common nonviral sexually transmitted disease. *T. vaginalis* is also known to facilitate the transmission of HIV (human immunodeficiency virus) and HPV (human papillomavirus) [59]. Among these compounds, **48** exhibited IC_50_ equal to 29 µM, while the simple chalcone and compounds **1** and **26** showed inferior activity. This data corroborates the importance of the presence and position of the amino group on ring A. Moreover, at 29 µM, **48** was also found to affect the growth kinetics of *T. vaginalis*. However, it was found to be toxic against vaginal epithelial cells HMVII in vitro, with a selectivity index of 0.72. At the same time, injection in the *Galleria mellonella* larvae indicated that **48** was not toxic to complex living organisms. The researchers tested **48** for its hemolytic effect and ability to induce ROS accumulation in *T. vaginalis* trophozoites in order to determine the mechanism responsible for this activity. However, the molecular targets remained unidentified. *T. vaginalis* induces ROS accumulation in cells as part of the immune response. Compound **48** was found to activate ROS production in neutrophils incubated with *T. vaginalis*, thus enhancing this immune response.

**Figure 31:**
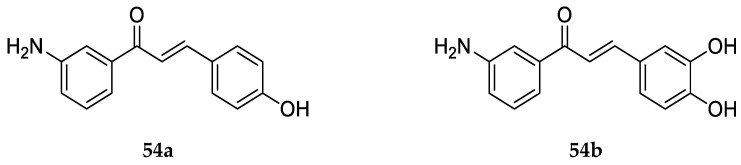
Chemical structures of 3-aminochalcone derivatives with substitution on ring B.

The hydroxy- and dihydroxy-substituted derivatives of 3-aminochalcones (Figure 31) have also been synthesized and tested for α-glucosidase inhibition by Seo at al. [47].

The IC_50_ values of **54a** and **54b** as α-glucosidase inhibitors were >200 µM each and greater than most of the chalcone derivatives tested in the manuscript and poor in comparison to 4-aminochalcone analogs **4a** and **4b**, with IC_50_ values 41 µM and 62.1 µM, respectively. According to the study, **54a** and **54b** only exhibited mild inhibitory activity against β-amylase; hence, they cannot act as reliable lead compounds for such inhibitors [47].

**Figure 32:**
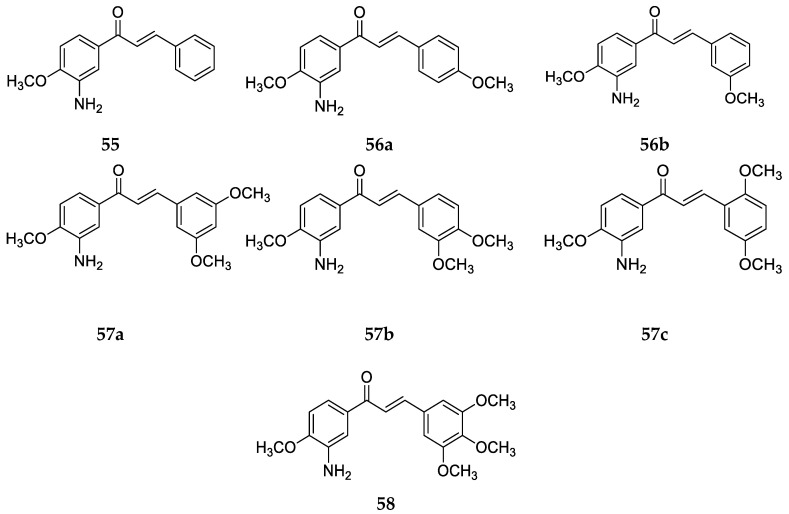
Chemical structures of 3-aminochalcone derivatives with methoxy substitution on rings A and B.

Pati et al. [60] synthesized and studied the cytotoxic properties of several 3-aminochalcones with different methoxy substitutions on both rings A and B (Figure 32).

The anticancer activity of compounds **55**–**58** was evaluated against murine melanoma B16 cells via the MTT colorimetric assay, and the IC_50_ values are given in Table 17.

The findings revealed trends in the cytotoxic properties of 3-aminochalcones **55**–**58**. The increase in the number of methoxy groups on ring B showed a marked increase in potency as IC_50_ values of **57a**–**c** are much lower than that of compounds **56a** and **56b**. However, this trend is only observable between the mentioned compounds, and compound **58** that has 3-methoxy groups on ring B shows a decreased inhibition compared to other compounds. Overall, **57c** was the most potent and **55**, without any substituent on ring B, was the least active compound among the 3-aminochalcones tested in this study. This might be due to some positions on ring B being favorable while others being unfavorable to the interactions between the compound and cancer cells [60].

**Figure 33:**
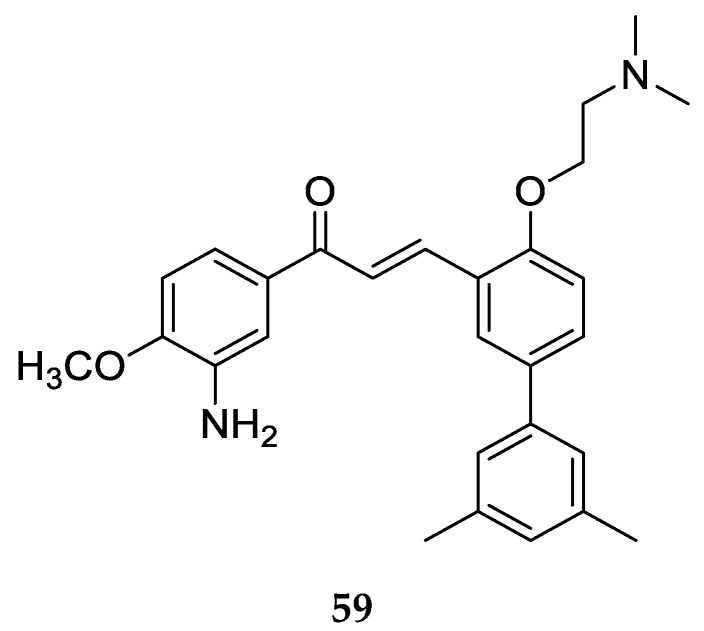
Chemical structures of the 3-aminochalcone derivative with substitution on ring B.

Finally, a 3-aminochalcone with a larger substituent on ring B was reported by Nielsen et al. [52] (Figure 33).

The antibacterial activity of compound **59** was tested in terms of MIC values against *Staphylococcus aureus* ATCC33591 (resistant to methicillin), *Enterococcus faecium* 17501 (vancomycin-resistant clinical isolate), and *Enterococcus faecium* ATCC29212, which was 10 µM for each strain. The 2-aminochalcone analog compound **45** showed better antibacterial activity against *S. aureus* and *E. faecium*, with MIC values of 5 µM each. The results reflect the relatively weaker antibacterial potential of **59**, the significance of the position of an amine group on ring A, and the possibility of using aminochalcones other than **59** as better leads for antibacterial agents, which brings our evaluation of aminochalcones with the -NH_2_ group on ring A to a conclusion.

### 2.2. Substitution on Ring B 

#### 2.2.1. 4′-Aminochalcone and Derivatives 

**Figure 34:**
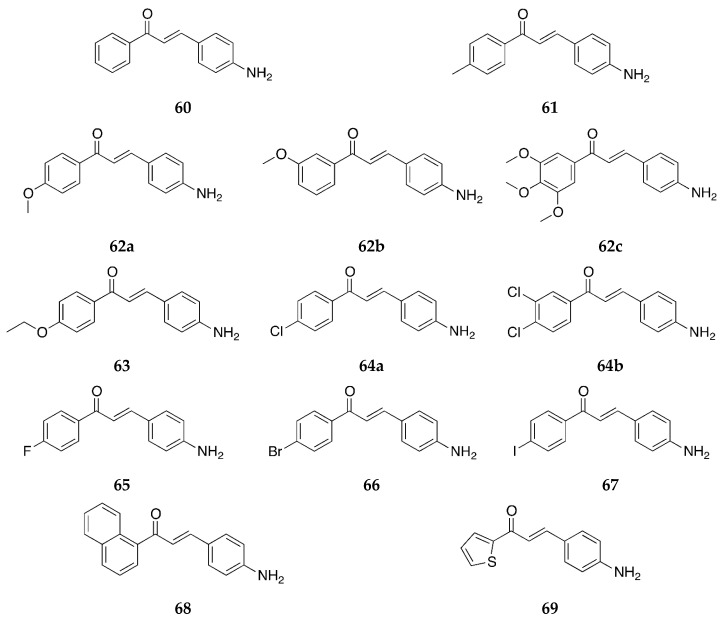
Chemical structures of 4′-aminochalcone and its derivatives with substitutions on ring A.

Chalcones with amino substitution on ring B have also been synthesized and tested for their bioactivity. Changing the position of -NH_2_ from one ring to the other changes the electronics of the compound, which may in turn change the way it interacts with biological targets. Romagnoli et al. [37] prepared a series of 4′-aminochalcones via the Claisen-Schmidt aldol condensation with different substitutions (Figure 34) and later converted them to their corresponding α-bromoacryloylamido derivatives to compare the cytotoxic activities. In this section, however, we will discuss only the 4′-aminochalcone precursors and their activities, which are shown in Table 18.

As discussed earlier, the simple 4-aminochalcone **1** was also reported in this paper. The IC_50_ values for 4-aminochalcone are shown in Table 19 in comparison to 4′-aminochalcone. 

Evidenced by the IC_50_ values, compound **1** has higher antiproliferative activity when compared with **60**. Shifting -NH_2_ from ring A to ring B decreases the anticancer activity of the chalcone. This observation is also seen in the methoxy-substituted compounds **62a**–**c** and their 4-aminochalcone analogs **3a**, **3c**, and **3i** discussed earlier, where the latter show better activity against all cell lines tested by Romagnoli et al. [37]. 4′-aminochalcone compounds, in general, showed very week antiproliferative activity, as is evident from the high IC_50_ values. From the tested compounds, a better activity was shown by **62c**, **63**, **64b**, and **68**. Both the methoxy- and ethoxy-substituted compounds showed better activity than the rest. The activity for the methoxy-substituted compounds increased as the substitution on ring A increased.

**Figure 35:**
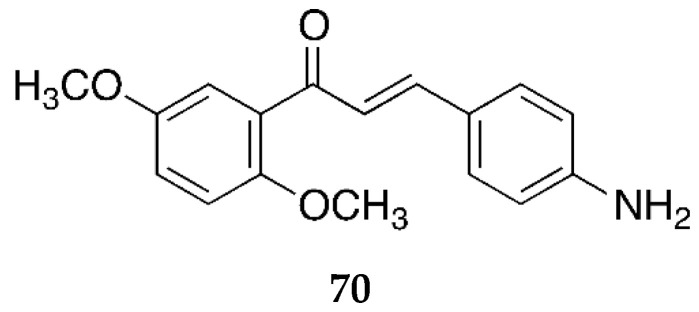
Chemical structures of 4′-aminochalcone derivative with methoxy substitutions on ring A.

**Figure 36:**
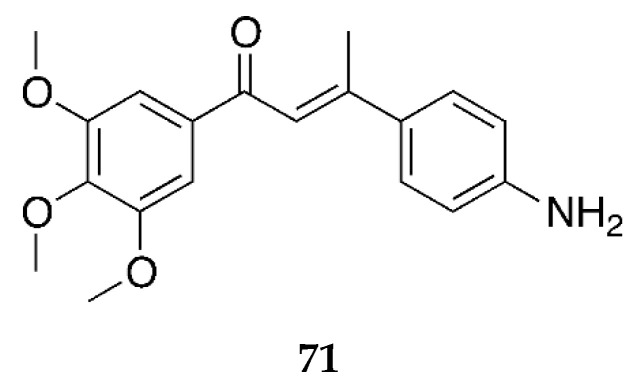
Chemical structures of the 4′-aminochalcone derivative with methoxy substitutions on ring A.

Some more methoxy-substituted compounds, including 3-aminochalcones, discussed earlier, were synthesized and analyzed by Pati et al. [60]. These compounds included **62c** (Figure 35) and **70** (Figure 36).

Cytotoxic activity, in the form of IC_50_ values, for **62c** and **70** against the murine melanoma B16 cells was reported to be 30 and >100 µM, respectively. Thus, neither of these compounds showed significant activity against the cancer cell line. However, it can be concluded that the cytotoxicity increases as the methoxy substitutions increase, as the activity increased threefold with the addition of a methoxy substituent on ring A. They also reported the activity against some 3-aminochalcones with methoxy substitutions including **57c** (IC_50_ against B16 cells = 5.6 µM), which showed much better activity when compared with **70**. The difference in the structures for the two compounds is the presence of the -NH_2_ group on ring A and an extra methoxy group on ring B for **57c**, which increases the cytotoxic activity by almost 18-fold. 

Like compound **62c,** Edwards et al. [61] reported an antimitotic ED value over 6 h against HeLa cells for compound **71** (Figure 36). 

The antimitotic activity for **62c** and **71** against the HeLa cells was reported to be 0.15 and 0.3 µg/mL, respectively. A methyl group on the beta carbon decreases the antimitotic activity of the chalcone by half. 

**Figure 37:**
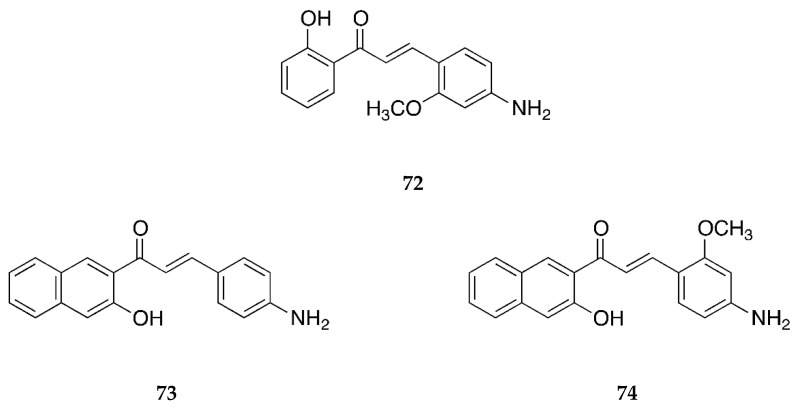
Chemical structures of the 4′-aminochalcone derivative with methoxy substitutions on ring A.

Radhakrishnan et al. [62] also synthesized some hydroxy and methoxy 4′-aminochalcone derivatives (Figure 37) via the Claisen-Schmidt reaction with subsequent reduction to yield the amines from the nitro compounds. They tested these compounds for antityrosinase activity in B16 cells.

Since tyrosinase is responsible for melanization in animals, tyrosinase inhibitors are useful for the treatment of such dermatological disorders, especially those associated with melanin hyperpigmentation and sunburn treatment. The tyrosinase inhibition activity in B16 cells was determined for **72**–**74** by measuring the rate of L-DOPA oxidation. The tyrosinase inhibitions for **72**, **73,** and **74** were reported to be 75.5 ± 1.12, 54.3 ± 2.01, and 39.6 ± 0.66%. Compound **72** exhibited the most potent tyrosinase inhibition activity (IC_50_ = 7.82 ± 0.42 µM), greater than the reference kojic acid (inhibition = 48.4 ± 0.32%; IC_50_ = 22.83 ± 0.66 µM). This can be due to the strong electron-donating substituents on the *ortho* and *para* positions on the ring B of **72**, which influences the electronic structure of ring B. The docking studies done in this investigation showed that these electron-donating groups increase the electron density of ring B through resonance, which results in more effective binding to the active site of the enzyme. While the inhibition for **73** and **74** is less than **72**, it is still greater than kojic acid for **73**. This change in activity occurs when the phenyl ring is replaced by a naphthyl ring. This suggests that the bulky naphthyl group hinders binding to the active site. These 4′-aminochalcones were concluded to be promising candidates for use as depigmentation agents and in the treatment of dermatological disorders. 

#### 2.2.2. 3′-Aminochalcone and Derivatives 

**Figure 38:**
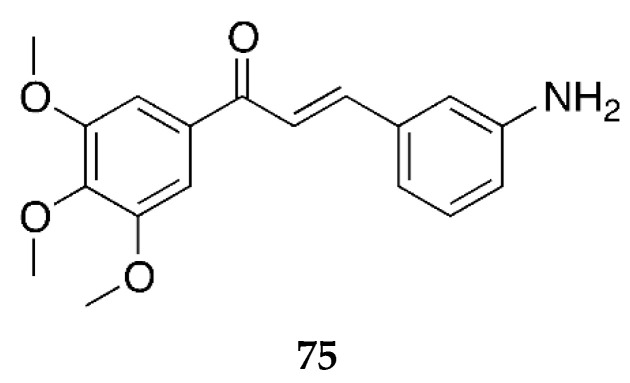
Chemical structures of the 3′-aminochalcone derivative with methoxy substitutions on ring A.

3′-Aminochalcones are chalcones with an -NH2 group on the 3′ position (on ring B). One such compound with methoxy substitutions on ring A (Figure 38) was synthesized, and its antimitotic activity was tested by Edwards et al. [61] against the HeLa cell line. 

The ED value over 6 h for **75** was 0.15 µg/mL, which is the same as its 4′-aminochalcone analog **62c**. Thus, the antimitotic potential for 3′- and 4′-aminochalcones was the same against this cell line. 

**Figure 39:**
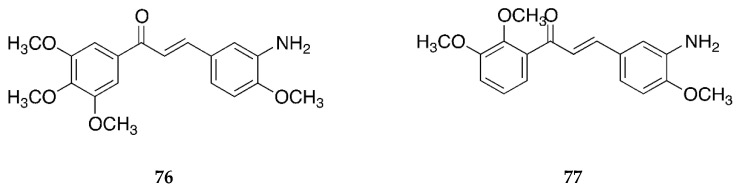
Chemical structures of 3′-aminochalcone derivative with methoxy substitutions on ring A and ring B.

Along with previously discussed 4′-aminochalcones, Pati et al. [60] also tested the antiproliferative activity of some 3′-aminochalcones (Figure 39) against the murine melanoma B16 cells. 

The IC_50_ values for **76** and **77** were 0.24 and 2.0 µM against the B16 cells. This activity is far greater than that of the 3-aminochalcones **55**–**58**, and the 4′-aminochalcones **62c** and **70** reported in the same paper suggested the appropriate positioning of the groups. Compound **76** was found to be at least 1000-fold more cytotoxic than compound **58**, a 3-aminochalcone with the same substitutions on opposite rings. Between **76** and **77**, the cytotoxicity increased with the addition of a methoxy group from **77** to **76**. Compound **76** showed the best activity among the compounds reported in the manuscript. 

**Figure 40:**
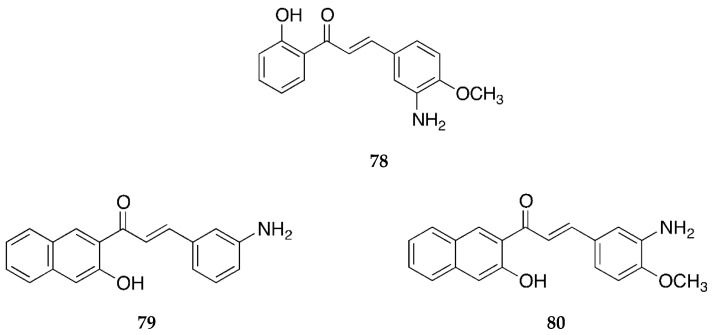
Chemical structures of 3′-aminochalcone derivatives with hydroxy and methoxy substitutions on ring A and ring B.

Radhakrishnan et al. [62] synthesized some 3′-aminochalcones (Figure 40) and tested their tyrosinase inhibition activity. 

Like **72**–**74**, compounds **78**–**80** were tested for their tyrosinase inhibition activity in B16 cells, which was reported to be 63.2 ± 0.12, 49.3 ± 0.45, and 58.5 ± 1.62, respectively. While the activity for these compounds was greater than the reference kojic acid (inhibition = 48.4 ± 0.32%; IC_50_ = 22.83 ± 0.66 µM), it was less than their 4′-aminochalcone analogs. Among compounds **78**, **79**, and **80**, compound **78** showed the greatest inhibition (IC_50_ = 9.75 ± 1.22 µM) comparative to that of **72**, which showed an IC_50_ value of 7.82 ± 0.42 µM. In conclusion, in this case, the 4′-aminochalcones exhibited more potent tyrosinase inhibitory activity. 

**Figure 41:**
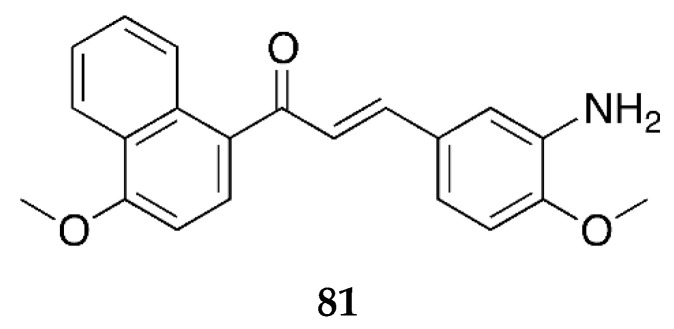
Chemical structures of 3′-aminochalcone derivatives with hydroxy and methoxy substitutions on ring A and ring B.

Wang et al. [63] in their paper discussed the synthesis and biological evaluation of **81** (Figure 41) along with other *N*-alkyl, *N,N*-dialkyl, and *N*-acyl compounds, which will be discussed in the later sections. 

Compound **81** was studied for their anticancer activity by targeting the tubulin colchicine binding site. Its antiproliferative activity was initially tested against human colon carcinoma cells HCT116 and human hepatocellular liver carcinoma cells HepG2. Compound **81** was found to be the most potent compound among all those tested, and its cytotoxicity was further tested against some cancer cell lines (DU145, B16-F10, MDA-MB-231, A549, and Hela), one normal (LO2) cell line, and one drug-resistant (HCT-8/T) cell line. Table 20 shows the IC_50_ values for **81** against these cell lines.

Colchicine was used as a positive control in this investigation. Chalcone **81** was the most potent compound against almost all cell lines in the manuscript. 1-(4-Methoxynaphthalen-1-yl)ethan-1-one, the naphthyl ketone used to synthesize **81**, showed much lower potency. Compound **81** showed potency almost 200-fold greater than colchicine (IC_50_ = 3.45 ± 0.35 µM) in prostate cancer cell line DU145. Similarly, in the cervical Hela cancer cell line, the activity of **81** was 100-fold greater than colchicine (IC_50_ = 8.89 ± 0.68 µM). The cytotoxicity of **81** was very similar to that of colchicine (IC_50_ = 5.53 ± 0.92 µM) for the normal cell line LO2, suggesting its selectivity to be similar to colchicine. Compound **81** was also found to be quite potent against the drug-resistant HCT-8/T cell line, with an IC_50_ value of 5.33 ± 0.86 µM, especially when compared to reference colchicine (IC_50_ = 227.40 ± 95.3 µM). Compound **81**′s tubulin polymerization inhibition was also tested, for which isolated pig brain tubulin was used. The study used five different concentrations of **81** and colchicine and evaluated the tubulin polymerization in vitro. The IC_50_ values for **81** and colchicine were 7.1 and 9.0 µM, respectively, which suggests that the molecular target of **81** is tubulin. This study concluded that **81** could serve as a candidate for cytotoxic drugs and tubulin polymerization inhibition. 

## 3. *N*-Alkyl and *N,N*-Dialkyl Aminochalcones 

After discussing the numerous -NH_2_-substituted aminochalcones, with substitutions at multiple positions on both the rings, we shift our gear and look at the aminochalcones that not only contain various functional groups on aromatic rings but also are *N*-alkyl or *N,N*-dialkyl. The discussion about these chalcones will be done like in the previous sections, where we will be discussing the alkylated aminochalcones with the amino group on position 4 of their ring A first. 

### 3.1. Substitution on Ring A

#### 3.1.1. *N*-alkyl Derivatives of 4-Aminochalcone 

**Figure 42:**
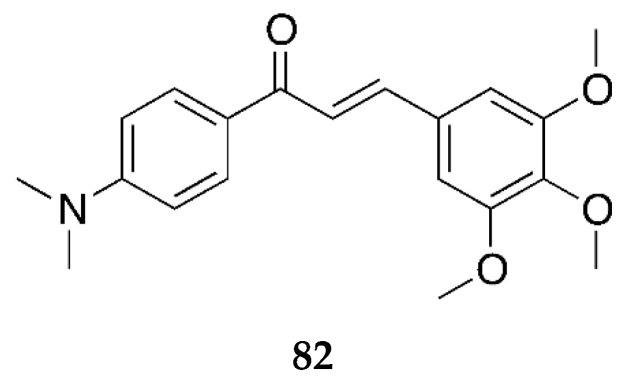
Chemical structure of the *N,N*-dimethyl 4-aminochalcone derivative with methoxy substitution on ring B.

An *N,N*-dimethylated aminochalcone (Figure 42) was synthesized and tested for its antimitotic activity against the HeLa cell line by Edwards et al. [61]. 

The ED value over 6 h for **82** against Hela cell line was 0.15 µg/mL. The reference drug, colchicine (concentration = 0.05 µg/mL), was 3 times more potent than the tested chalcone. The simple aminochalcone analog for **82** was not tested in this investigation. 

**Figure 43:**
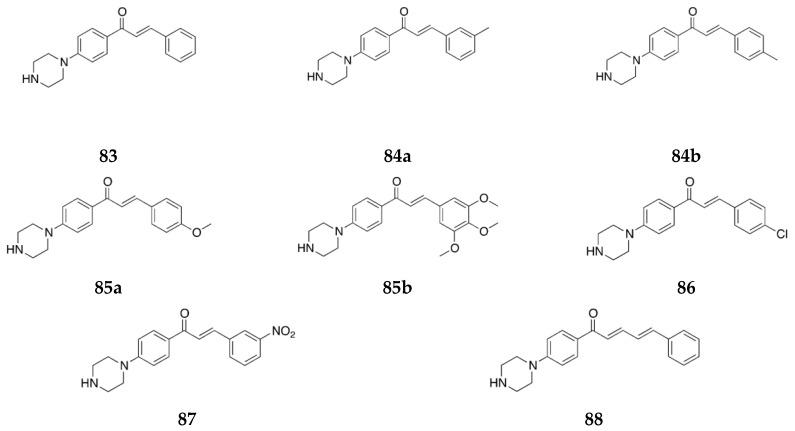
Chemical structures of *N,N*-dialkylated 4-aminochalcone derivatives.

Tomar et al. [64] synthesized several piperazine containing chalcones (Figure 43) via the Claisen-Schmidt condensation reaction and tested their antimicrobial potential. 

Since piperazine derivatives have been reported to show antifungal activity [65], it was hypothesized that chalcones, with their diverse therapeutic activities, would give potent compounds when coupled with piperazine. Thus, the synthesized compounds were tested for their antibacterial activity against *S. aureus* 209 p, *E. coli* ESS 2231, *Proteus vulgaris*, *Klebsiella pneumoniae*, and *Aspergillus fumigatus*. Their antifungal activity was tested against *Candida albicans*, *C. albicans* ATCC 10231, and *Candida glabrata*. The MIC values obtained are summarized in Table 21. 

Ciprofloxacin was used as the standard drug for antibacterial activity, and fluconazole was used as the standard for antifungal activity. The MIC value for ciprofloxacin against all five bacteria cultures was 100 µg/mL, and fluconazole’s MIC value against the three fungi strains was 50 µg/mL. The compounds **84a**, **85a**, and **87** showed very good activity against *S. aureus*, and **83**, **84a**, **85a**, and **87** showed very good potency against *E. coli*. Generally, compounds **83**, **84a**, **85a**, and **87** were found active against all strains. Most of the values were comparable to, if not many folds better than, the reference drugs. Comparing the simple compound with no substitution on ring B, **83** with **87,** which has a nitro substitution on the *meta* position, show that this substitution is well tolerated against *S. aureus* but not against some other strains such as *Proteus vulgaris* and *Klebsiella pneumoniae*. The methoxy substitution causes the activity against *S. aureus* to increase as well. However, with multiple methoxy substitutions in **85b**, the activity decreases. The simple chalcone **83** showed the best activity against all three fungal strains *Candida albicans*, *C. albicans* ATCC 10231, and *Candida glabrata*, which was approximately 10 times greater than fluconazole. Compound **1**, the simple 4-aminochalcone, was also tested against a strain of *E. coli* bacteria and had shown an MIC of 500 µg/mL. Thus, replacing the -NH_2_ group with a piperazine ring increases the potency of the chalcone compound. 

**Figure 44:**
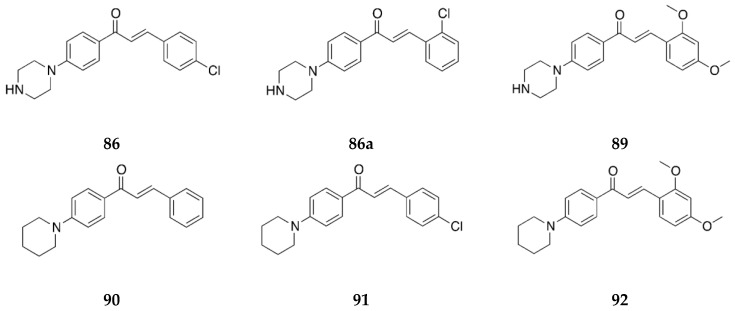
Chemical structures of *N,N*-dialkylated 4-aminochalcone derivatives.

Some more compounds with N-containing rings substituted on position 4 of ring A (Figure 44) were synthesized by Liu et al. [66] via the base-catalyzed Claisen-Schmidt condensation.

Liu et al. [66] tested these compounds for their ability to selectively inhibit P-glycoprotein (Pgp; ABCB1) and breast cancer-resistant protein (BCRP; ABCG2). These proteins belong to the ATP-binding cassette (ABC) family of proteins and are involved in actively flowing anticancer drugs out from their target cancer cells, thus limiting their efficacy. Multidrug resistance (MDR) is a phenomenon where cancer cells become resistant to anticancer drugs. MDR has been ascribed to overexpression efflux transporters such as Pgp. Pgp inhibition was evaluated by an in vitro calcein-AM assay carried out on Mardin–Darby canine kidney (MDCK) cells, and BCRP inhibition was evaluated by the mitoxantrone (MX) accumulation assay carried out in a human breast cancer cell line (MCF-7). The Pgp inhibition for these compounds was compared with the standard reference verapamil (% calcein accumulation = 188 ± 18). Compound **89** had activity comparable to verapamil, with % calcein accumulation equal to 216 ± 13. In another analysis to evaluate the accumulation of doxorubicin (DOX) in MCF-7 cells, the amount of DOX uptake measured by the level of fluorescence showed that **89**, among others, had Pgp inhibitory potential. The investigation to evaluate BCRP showed that tested compounds, including **89**, did nothing to increase MX accumulation in the cell line. However, the researchers concluded that, among all of the tested compounds, **89** had the potential to act as a dual inhibitor of Pgp and BCRP, as it was comparable to verapamil in increasing calcein uptake and nearly as equally efficient as fumitremorgin C (FTC) in increasing MX accumulation. Compound **89** also did not display significant cytotoxicity against MCF-7 and HCT116 cells (IC_50_ = 16.3 and 13.4 µM respectively), which was a positive observation. 

**Figure 45:**
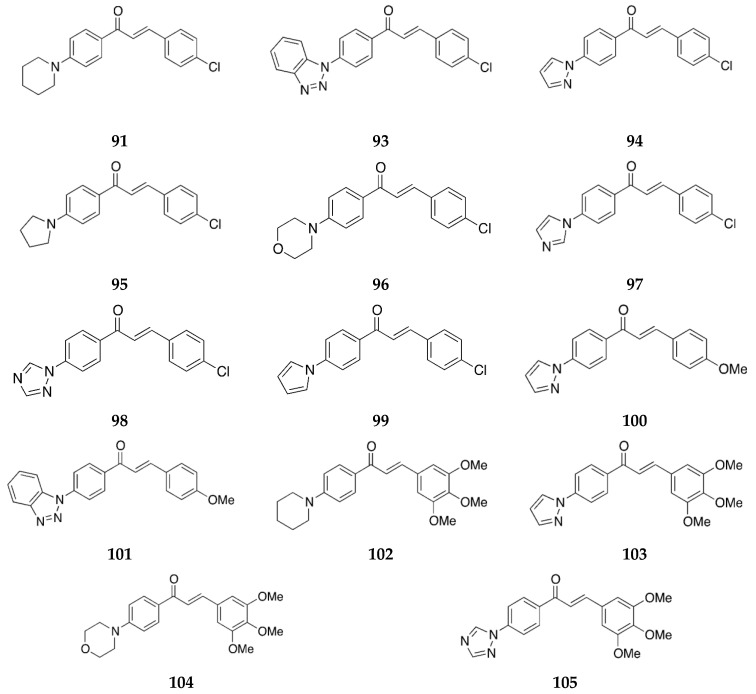
Chemical structures of *N,N*-dialkylated 4-aminochalcone derivatives.

Mishra et al. [67] synthesized fourteen 4-aminochalcones, with alkylation on the nitrogen atom (Figure 45) via the Claisen-Schmidt condensation method and tested these for their antimalarial activity. In some of these compounds, the N atom was part of an aromatic ring. Piperidine-substituted compound **91**, discussed earlier, was also synthesized and tested in this manuscript.

The antimalarial activity for compounds **91** and **93**–**105** depends on their ability to inhibit the parasitic enzyme cysteine protease. The IC_50_ values against the plasmodial effect of the malarial parasite are shown in Table 22.

All compounds **91** and **93**–**105** have heterocycles substituted on position 4. Among these compounds, somewhere, the nitrogen atom on position 4 of ring A is bonded with another nitrogen atom. As can be seen from the IC_50_ values, within the chloro-substituted compounds, **93**, **98**, and **99** showed very good antiplasmodial activity, with the triazole substituted chalcone **98** showing the best activity among all. The morpholine-substituted chalcone **96** displayed the lowest activity (IC_50_ = 9.1 µg/mL). In methoxy-substituted compounds, inhibition increases with the addition of methoxy groups, as can be seen from the IC_50_ values of **100** (monomethoxy) and **103** (trimethoxy). The relationship is unclear when comparing between the compounds with chloro and methoxy substituents on ring B. Comparing between **96** and **104**, the activity for the trimethoxy-substituted compound **105** is higher. However, comparing between **98** and **105**, chloro-substituted **98** showed better activity. The benzotriazole substituted chalcones **93** and **101** were not very potent. The activity for **98** was better than all the tested compounds. The potency of this compound suggests that nitrogen-containing small lipophilic groups enhance the antiplasmodial activity. Even though other compounds also exist with three nitrogen atoms in the heterocycle, such as **93** and **101**, it was presumed that, in **98**, the orientation and spacing of the nitrogen atoms in the chalcone allowed it to interact with the histidine residue at the active site of the cysteine protease enzyme. Even though more study needs to be done on these chalcones, they are promising antimalarial agents.

**Figure 46:**
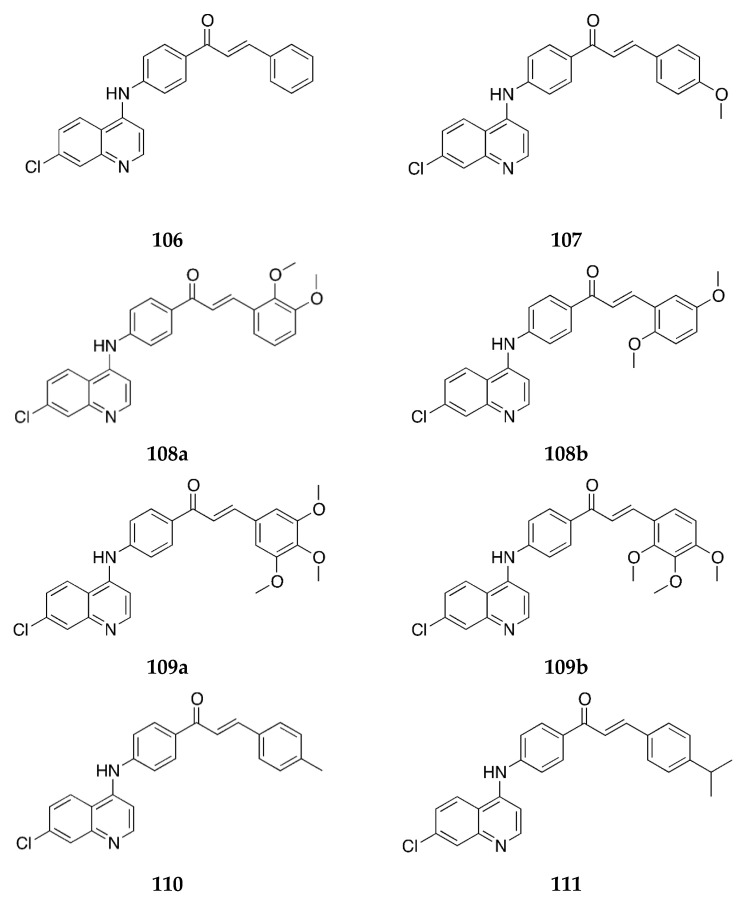
Chemical structures of quinolinyl-4-aminochalcones derivatives.

**Figure 47:**
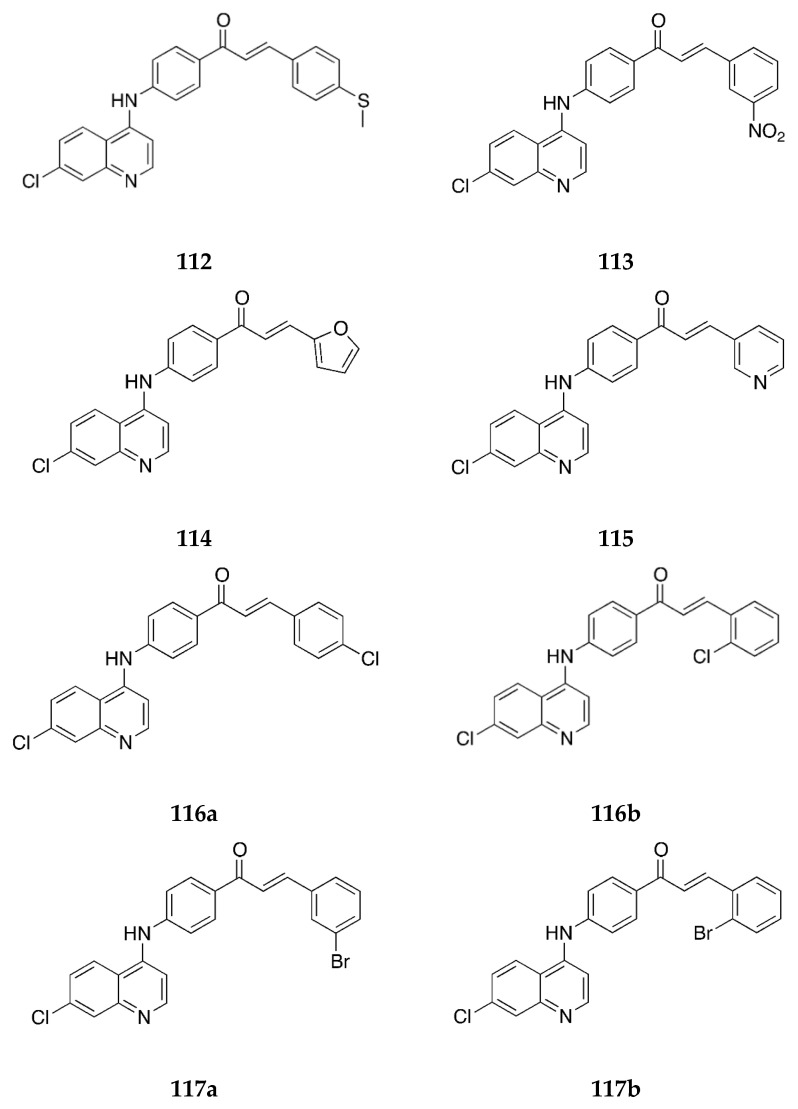
Chemical structures of quinolinyl-4-aminochalcones derivatives.

Sharma et al. [68] synthesized some substituted quinolinyl chalcones and quinolinyl pyrimidines. In this review, we will only be discussing the amino-linked quinolinyl chalcones. Figure 46 shows the unsubstituted chalcone and chalcones with methoxy and alkyl substitutions. Figure 47 shows chalcones with heterocycles as ring B and those with chloro-, bromo-, thio-, and nitro- substitutions on ring B.

These compounds **106**–**117** were tested in vitro for their antitubercular and antimalarial activities by screening the compounds against H_37_Rv strains of the *M. tuberculosis* and NF-54 strains of *P. falciparum*. These compounds did not show any significant activity against the malarial strain *P. falciparum*. In addition to the 4-amino linked quinolinyl chalcones shown in Figure 46 and Figure 47, Sharma et al. [68] also synthesized 4-oxo-linked quinolinyl chalcones. 4-amino-linked chalcones were more active than the 4-oxo-linked ones. Among the 4-amino-linked quinolinyl chalcones, compounds **108a** and **108b** exhibited a higher activity, with MIC being 3.15 µg/mL against the *M. tuberculosis* strain. This activity was 16-folds greater than that of pyrazinamide (MIC = 50 µg/mL), a medication used to treat tuberculosis. These dimethoxy quinolinyl chalcones were nontoxic towards the VERO and MBMDM (mouse bone marrow-derived macrophages) cell lines. Moreover, **116a** exhibited an MIC of 6.25 µg/mL, which shows that it is active against the *M. tuberculosis* strain as well. 

**Figure 48:**
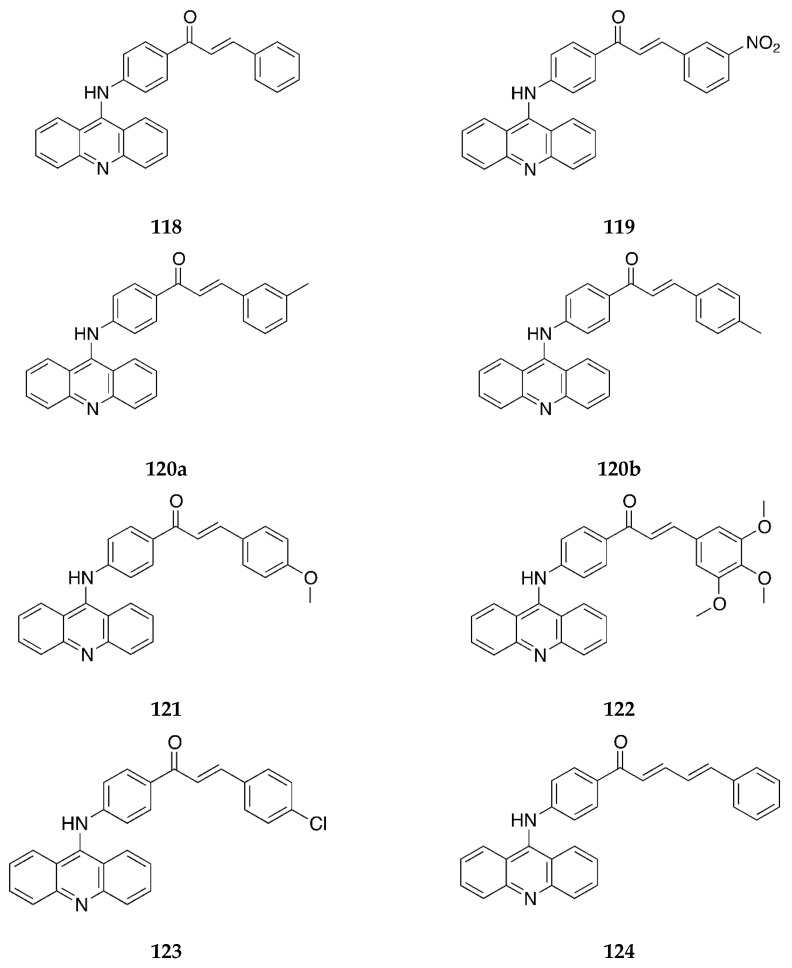
Chemical structures of 4-aminochalcones derivative chalcones with an acridinyl group.

Amino-linked chalcones with acridinyl group were synthesized by Tomar et al. [69] and tested for their antimalarial activity. The simple aminochalcones were synthesized via the Claisen-Schmidt reaction and then treated with 9-chloroacridine to yield the final chalcones. In addition to the 4-amino-linked chalcones (Figure 48), the authors also reported 3-amino-linked chalcones, which will be discussed in their section. 

The in vitro antimalarial activity for compounds **118**–**124** was reported on cultures of the *P. falciparum* NF-54 strain using chloroquine as the reference drug. In this investigation, Tomar et al. [69] reported the mean number of rings, trophozoites, and schizonts recorded per 100 parasites after incubation for 38 h and the percentage of schizont maturation inhibition at different concentrations of the chalcones. These compounds showed 100% inhibition of schizont maturation at 100, 50, and 10 µg/mL concentrations. At 2.0 µg/mL concentration, compounds **119**, **120a**, and **123** showed no inhibition. Compounds **118** and **120b** had 42.8% schizont maturation inhibition. Chalcone **121** showed 85.7%, 71.4%, and 14.3% schizont maturation inhibition at 2.0, 0.4, and 0.08 µg/mL, respectively. Compound **122** had percent inhibitions of 57.1 and 5.7 against 2.0 and 0.4 µg/mL concentrations. Compound **124** had an inhibition values of 64.3% against 2.0 µg/mL and 0% against 0.4 µg/mL. Among these chalcones, **121** exhibited good antimalarial activity, which is not surprising since it is the methoxy-substituted derivative. However, the potency decreased in the trimethoxy chalcone. Compound **121** was also tested against the chloroquine-resistant rodent malaria parasite *Plasmodium yoelii* (strain N-67) in a Swiss mice model. The authors concluded that these compounds, particularly **121**, can be used as antimalarial drugs but that it would be good to first profile these against some normal human cell lines.

**Figure 49:**
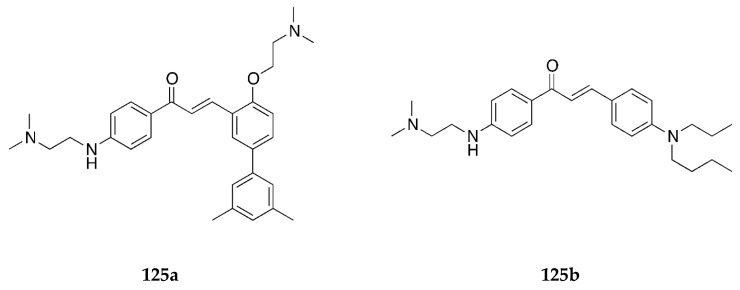
Chemical structures of mono alkylated 4-aminochalcones derivatives.

Lastly, for our alkylated 4-aminochalcones, compounds **125a** and **125b** (Figure 49) were reported by Nielsen et al. [52]. 

Compounds **125a** and **125b** were tested for their antibacterial potential against the *S. aureus* ATCC33591 (resistant to methicillin), *E. faecium* 17,501 (vancomycin-resistant clinical isolate), *E. faecium* ATCC29212, and *E. coli* ATCC25922 bacterial strains. The MICs of **125a** against these strains were 10, 10, 20, and 20 µM, respectively. The MIC for **125b** was 10 µM against *S. aureus*, *E. faecium* 17501, and *E. faecium* ATCC29212. These chalcones were not found to be very potent against bacteria, especially when compared with other compounds tested in the same investigation. 

#### 3.1.2. *N*-Alkyl Derivatives of 3-Aminochalcone 

**Figure 50:**
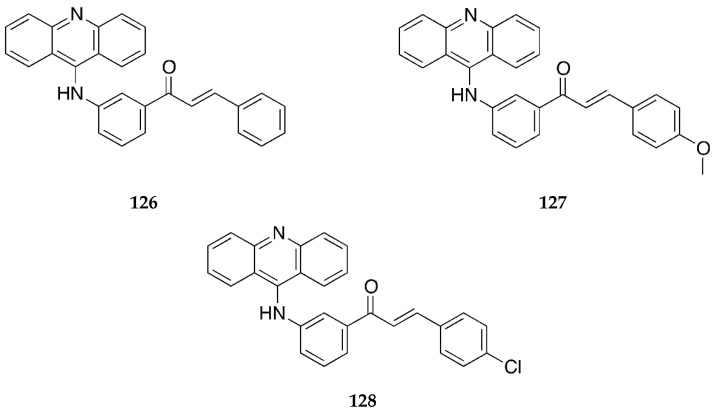
Chemical structures of 3-aminochalcones derivative chalcones with an acridinyl group.

Tomar et al. [69] reported 3-amino-linked chalcones (Figure 50) and tested these for their antimalarial activity.

Compounds **126**–**128** are chalcones with an acridinyl group linked to the position 3 on ring A of the chalcone via the amino group. The in vitro antimalarial activity for these compounds was also tested using cultures of the *P. falciparum* NF-54 strain, like compounds **118**–**124**. With compounds **126**–**128**, the percent schizont maturation was 100% for 100, 50, and 10 µg/mL. Compounds **126** and **127** showed percent inhibitions of 97.1% and 71.4% at 2.0 µg/mL and of 4.3% and 7.1% at 0.4 µg/mL concentration. Compound **128** showed no inhibition at 2.0 µg/mL. Among these acridinyl-containing 3-aminochalcones, **127** showed the best activity. Comparing the activity of **127** with the acridinyl-containing 4-aminochalcone **121** discussed earlier, it is evident that the 4-amino-linked analog **121** was more potent. At the 0.4 µg/mL concentration, **121** was 10-folds more active than **127**. This observation is in line with the previous observations made when comparing chalcones with amino groups at different positions.

**Figure 51:**
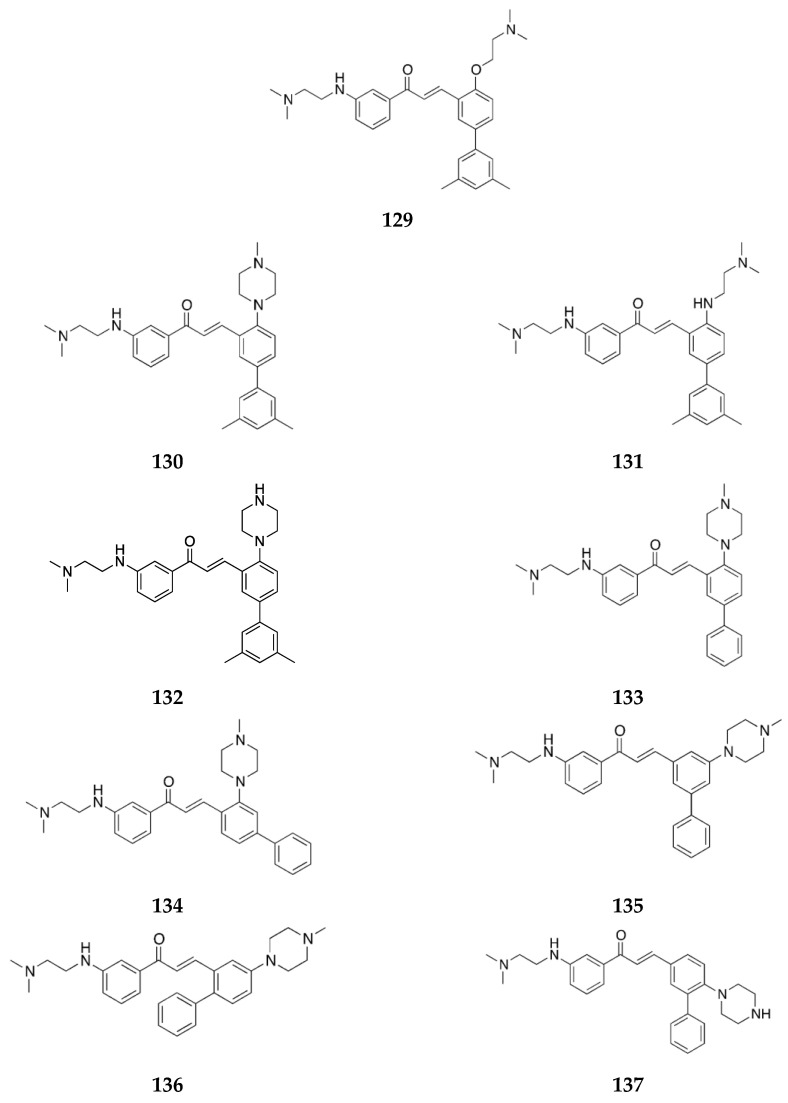
Chemical structures of 3-aminochalcones derivative chalcones with an acridinyl group.

Nielsen et al. [52] synthesized and tested more mono alkylated 3-aminochalcone analogs (Figure 51) of **125** and tested their antibacterial activities.

Compounds **129**–**137** were tested against gram-positive and -negative bacteria, namely, *S. aureus* ATCC33591 (resistant to methicillin), *E. faecium* 17501 (vancomycin-resistant clinical isolate), *E. faecium* ATCC29212, and *E. coli* ATCC25922. Table 23 shows the MIC. 

Mono alkyl aminochalcones **129**–**132** expressed better antibacterial activity as compared to the latter half of the compounds. Among these, **132** had a better activity for all cell lines. This potent compound has piperazine on ring B, which may contribute to its increased activity because of its basicity and by having a greater degree of protonation. Comparing **129** with **132** shows that the piperazine ring increases the potency of the compound. The biological potency of piperazine-containing compounds was also observed in **83**, **89**, and related compounds. The reason for the lower activity for the latter half of the compounds was concluded to be the unfavorable orientation of the molecule in the cell membrane. Comparing compounds **137** and **132**, the activity for **137** is many folds lower than **132**. Similarly, the activity for **134** is lower than that for **133** against the *E. faecium* 17501 strain. In both of these cases, the activity decreases as the piperazine and phenyl rings on ring B move closer to each other. The authors hypothesized that this could be due to the lipophilic and hydrophilic parts of the molecule coming closer together, making the fusion of these molecules with the cell membrane difficult. However, this trend does not hold when a comparison is made between compounds **136** and **135**, where the activity actually increases as the rings move closer. 

**Figure 52:**
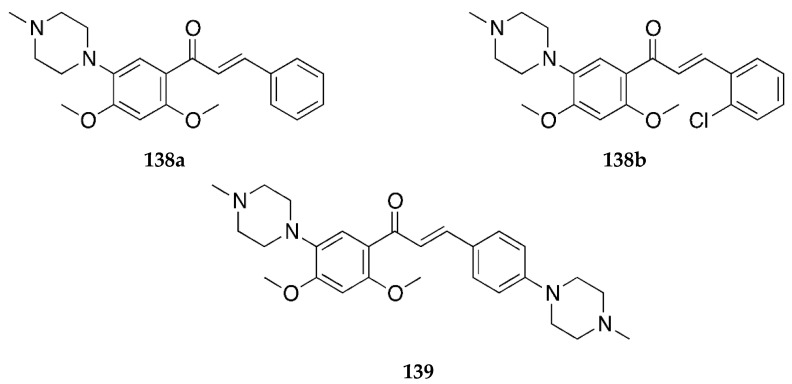
Chemical structures of *N,N*-dialkyl-3-aminochalcone derivatives.

Alkylated 4-aminochalcones (**86**–**92**) synthesized by Liu et al. [66] have been discussed in the earlier section. In the same manuscript, three N-alkylated 3-aminochalcones (Figure 52) were also synthesized and discussed for their ability to selectively inhibit P-glycoprotein (Pgp; ABCB1) and breast cancer-resistant protein (BCRP; ABCG2). 

The activity for all three compounds, **138a**, **138b**, and **139**, was not comparable to the reference verapamil or previously discussed **89**. Compared to **89**, these compounds have an alkylated piperazine ring and the methoxy substitutions are on ring A instead of ring B, which might change the way it interacted with Pgp.

#### 3.1.3. Derivatives of 2-Aminochalcone 

**Figure 53:**
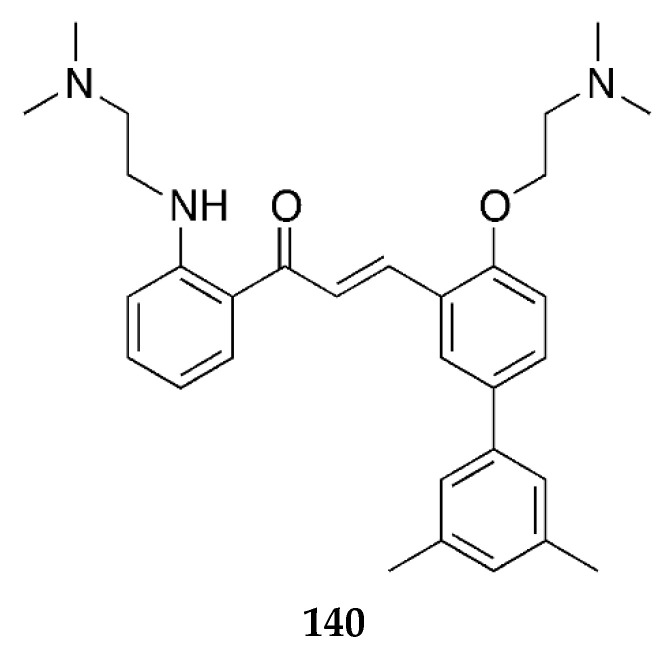
Chemical structure of the mono alkylated 2-aminochalcones derivative.

Only one alkylated 2-aminochalcone (Figure 53) was found in the literature. It is an analog of **125** and **129** reported by Nielsen et al. [52] and was tested for its antibacterial activity against the *S. aureus* ATCC33591 (resistant to methicillin), *E. faecium* 17501 (vancomycin-resistant clinical isolate), *E. faecium* ATCC29212, and *E. coli* ATCC25922 bacterial strains.

The MIC for **140** against all four bacterial strains was 10 µM. The activity for **140** was better than that for the N-alkylated 4-aminochalcone **125**. Compounds **125** and **129** showed similar activity against these strains. 

### 3.2. Substitution on Ring B

#### 3.2.1. *N*-Alkyl Derivatives of 4′-Aminochalcone 

**Figure 54:**
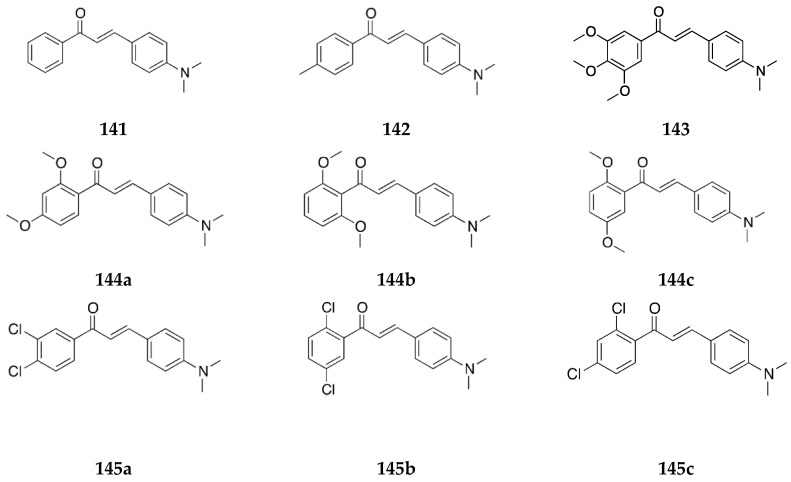
Chemical structure of *N,N*-dimethyl 4′-aminochalcones derivatives.

Simple *N,N*-dialkyl-4′-aminochalcones (Figure 54) have been reported in the literature by Rojas et al. [70]. These compounds have a dimethylamino group on position 4′ of ring B and different substitutions on ring A. 

Nitrogen intermediates such as NO (nitric oxide) play an imperative role in inflammatory reactions of the body. Influencing the expression of enzymes involved in the production of NO and prostaglandin E_2_ could lead to the development of anti-inflammatory drugs. Rojas et al. [70] tested these nine compounds’ inhibition activity in vitro against NO and PGE_2_ production using the mouse macrophage cell line RAW 264.7. Table 24 shows the inhibitory activity. 

The IC_50_ values for only two dimethoxy analog **144b** and **144c** were determined as they showed percentage inhibition greater than 50%, with IC_50_ values 0.7 and 0.6 µM, respectively. The rest of the compounds were inactive. While **144b** showed good activity against NO, it was rather poor in inhibiting the production of PGE_2_ (%I = 3.5 ± 3.5). Compound **144c** was the most potent inhibitor of NO and PGE_2_ production; however, it was less potent than the standard drug dexamethasone. It was also found that the dimethylamino-chalcone derivatives **144a**–**c** were not cytotoxic as they did not affect the cellular viability. The addition of methoxy substitutions on ring A increased the potency of the compound. This is an observation that was also made earlier, regarding the potency of the 4-aminochalcone series. All in all, the dimethoxy-substituted derivatives of *N,N*-dimethyl-4′-aminochalcone, **144b** and **144c**, can be promising leads to anti-inflammatory agents.

**Figure 55:**
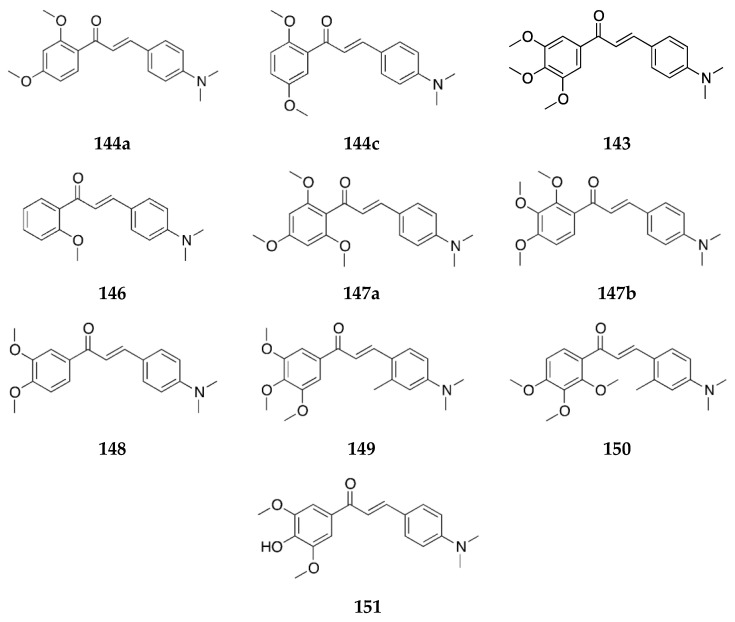
Chemical structure of *N,N*-dimethyl-4′-aminochalcone derivatives with methoxy substitutions on ring A.

In 1990, Edwards et al. [61] published a paper analyzing almost one hundred compounds for their antimitotic activity. The biggest class of compounds discussed were the 4′-aminochalcones with different methoxy substitutions on their ring A, among other variations. Figure 55 shows the compounds with the *N,N*-dimethyl group on position 4′ and methoxy substitutions on ring A.

**Figure 56:**
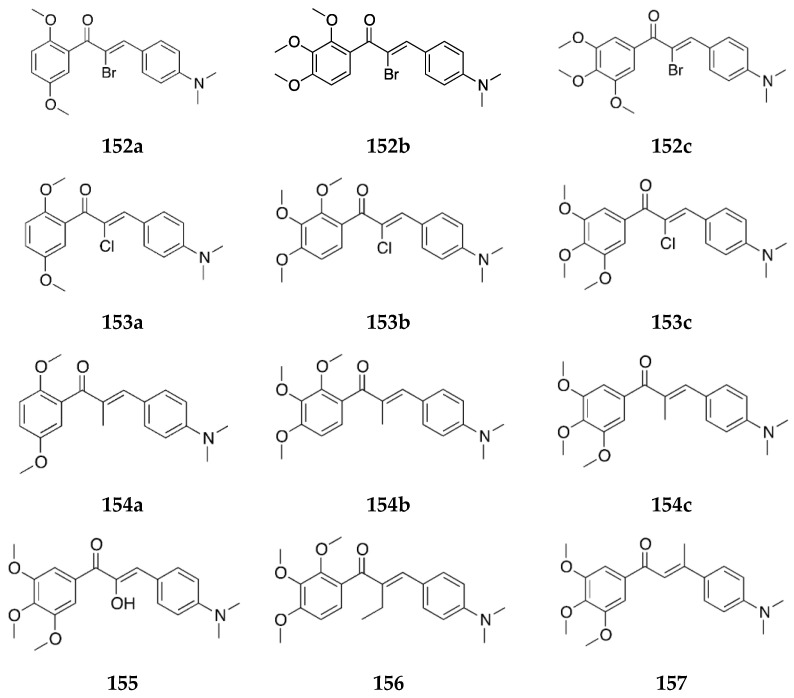
Chemical structure of *N,N*-dimethyl-4′-aminochalcone derivatives with methoxy substitutions on ring A and substitutions on C=C of chalcone.

In addition to those in Figure 55, Edwards et al. [61] also reported several *N,N*-dimethyl-4′-aminochalcones with substitutions on the C=C bond of the chalcone group (Figure 56).

**Figure 57:**
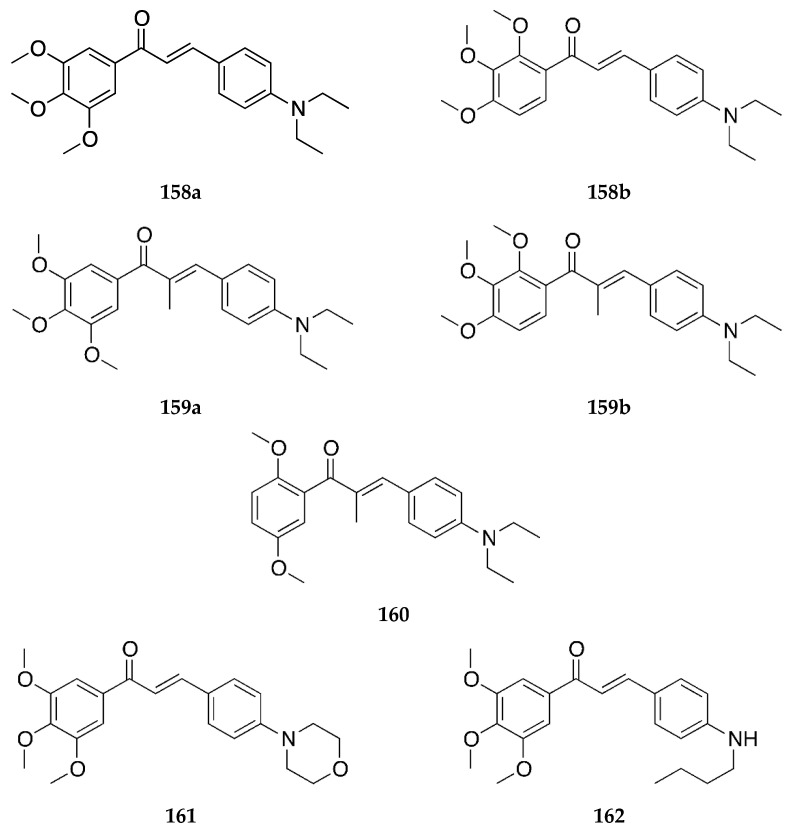
Chemical structure of other alkylated 4′-aminochalcone derivatives with methoxy substitutions on ring A.

In addition to the dimethylamino-chalcone compounds, the antimitotic activity of some diethylamino-chalcone compounds was also reported along with two other alkyl substitutions on the amino group at position 4′ (Figure 57).

All compounds **144a**, **144c**, **143**, and **146**–**162** (Figure 55, Figure 56 and Figure 57) were tested for their antimitotic activity via an in vitro assay system using HeLa cells. The cell cycle for this asynchronous line is 24 h, and mitosis requires 1 h. Colchicine was used as the reference drug, which stops mitosis at a concentration of 0.05 µg/mL. The activity for these chalcones was reported as the equivalent dose (ED), i.e., the concentration of the test compound with activity equal to 0.05 µg/mL of colchicine. The ED values are shown in Table 25. They also determined the irreversibility of the activity for some compounds by treating HeLa cells with the test compounds for 1 h, by washing them, and by subsequently checking the percentage of cells after 24 h, the results for which can be seen in the paper.

The first alkylated chalcone discussed in this paper, **82**, is an *N,N*-dimethylated aminochalcone (Figure 42). The dimethylamino group is on ring A instead of B. The analog for **82** in our current discussion is compound **143**. The 4′-aminochalcone **143** is 10-fold more active than **82** (ED = 0.15 µg/mL). In these compounds, the groups on both rings are electron-donating. Thus, more electron-donating groups on ring A give better activity. The non-alkylated 4-aminochalcone analog of **143**, **62c**, was discussed earlier. Its ED value was reported to be 0.15 µg/mL. The dimethylated aminochalone **143** is 10-fold more active than the -NH_2_-substituted analog **62c**. Apart from dimethylation, other alkylations were also performed on the nitrogen at position 4′ of the chalcone. The *N,N*-diethyl compound **158a** had an ED value of 0.0075 µg/mL; thus, it was twice as potent as **143**. However, for the 2,3,4-trimethoxy analogs, **147b** and **158b**, the relationship is the opposite, with the *N,N*-dimethyl 4′-aminochalcone **147b** being more potent; this may be a result of sterics. The morpholine-substituted chalcone **161** is less active than both **143** and **158a**. The ring conformation of the ethyl groups bonded with the electronegative oxygen atom decreases the activity by 830-fold, which is a huge decrease. This shows that the free conformation of *N,N*-diethyl is crucial for potency. A comparison of **158a** and **162** again exhibits the importance of the diethyl group. As can be seen, there are multiple variations of methoxy substitutions on the tested chalcones. The general trend showed that the 3,4,5-trimethoxy-substituted chalcones are more active against the HeLa cell line than their counterparts. Edwards et al. [61] also tested some chalcones with substitutions on their α-carbon atom, shown in Figure 56 and Figure 57. No clear trend was observed while comparing the activities of these compounds. However, they were quite potent with low ED values. The two most potent compounds of the investigation, **152c** and **154a** (ED value = 0.0038 µg/mL), had bromo and methyl substitutions on α-carbon. The methyl substitution on the β-carbon of the chalcone did not prove to be as successful. Compound **157** had an ED value of >100. Inactivity of **157** can be attributed to the bulky group attached to β-carbon. The inhibition activity may depend on the interaction of HeLa cells with β-carbon. This could also explain why the activity was enhanced upon substitution of α-carbon. Conclusively, the *N,N*-dialkyl-4′-aminochalcones are superb candidates as antimitotic agents and could be further finetuned to optimize their activity. 

**Figure 58:**
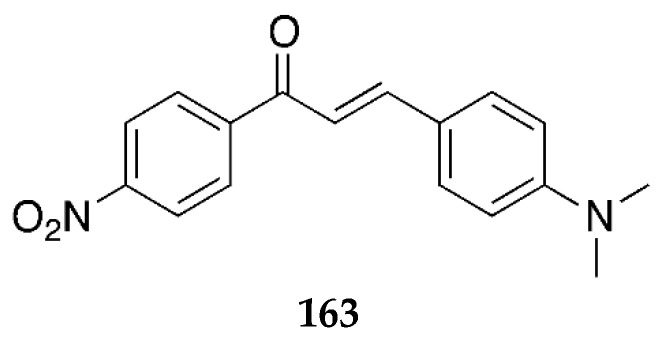
Chemical structure of the *N,N*-dimethyl-4′-aminochalcone derivative with nitro substitution on ring A.

Compound **163** (Figure 58) was synthesized by Doan et al. [71] and tested for its antioxidant and antimicrobial activities.

Apart from **163**, the compounds reported by Doan et al. [71] were methoxy and hydroxy chalcones. Compound **163** did not show any significant antioxidant or antimicrobial activity. 

**Figure 59:**
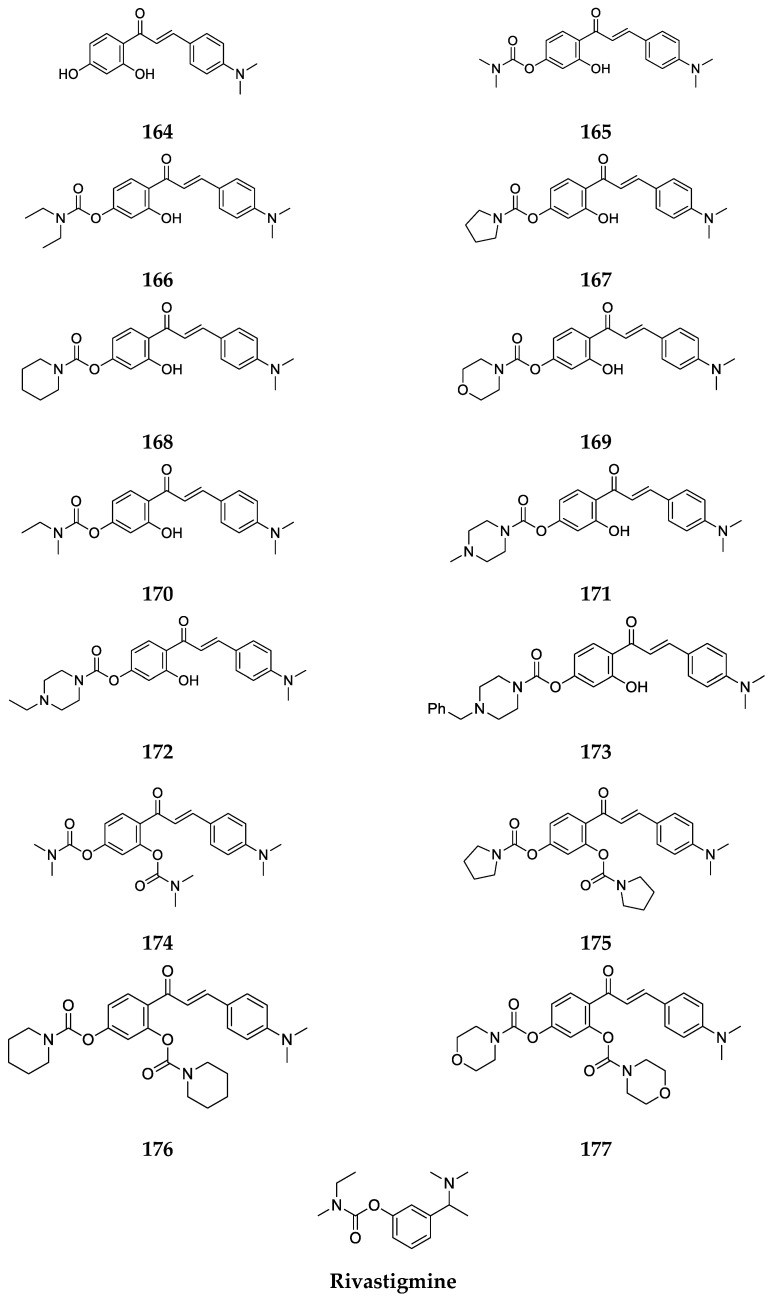
Chemical structure of *N,N*-dimethylated 4′-aminochalcone derivatives.

More *N,N*-dimethyl chalcone compounds considered to be 4-aminochalcone-rivastigmine hybrids (Figure 59) with substitution on the position 4′ of ring A were synthesized by Xiao et al. [72] and tested for their activity against Alzheimer’s disease (AD). 

**Figure 60:**
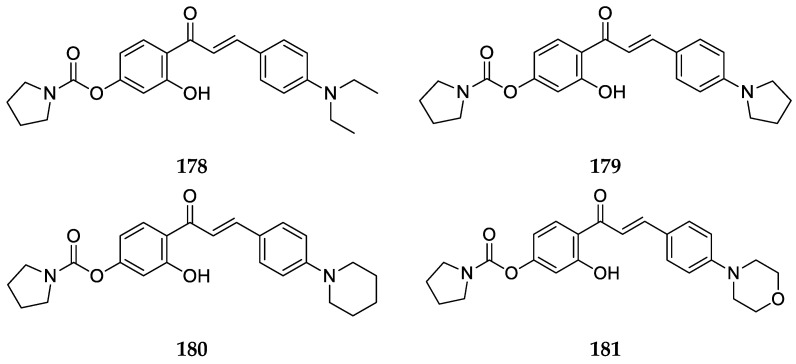
Chemical structure of *N, N*-dialkyl 4′-aminochalcone derivatives.

Some more *N,N*-dialkyl compounds were synthesized for the same investigation (Figure 60).

Compound **164** is the precursor, as it does not contain the carbamate functionality and rivastigmine, a cholinesterase inhibitor used for the treatment of AD and Parkinson’s disease. Multiple factors are involved in the onset and progression of AD, which includes deficits of acetylcholine (ACh), amyloid-β (Aβ) deposits, oxidative stress, and imbalance in the regulation of biometals. Some current therapies to combat AD include treatment with acetylcholinesterase inhibitors (AChEIs), for example, donepezil, rivastigmine, and galanthamine. In addition to this, monoamine oxidase (MAO) inhibitors can act as a potential treatment for AD due to their capacity to inhibit oxidative stress. The high expression of MAO in tissues causes oxidative stress by increasing the level of free radicals. The findings related to the ability of the chalcone analog to inhibit cholinesterase (ChE) and MAO lead these researchers to synthesize a series of chalcone-rivastigmine hybrids. These compounds were tested for their biological activities, namely, inhibition of cholinesterase, antioxidant and metal chelating effects, inhibitory effects on Aβ aggregation and MAO, and the ability to cross the blood–brain barrier (BBB).

The compounds’ inhibitory activity was tested against AChE, from the *electric eel*, and butyrylcholinesterase (BuChE) from *rat serum*, along with their oxygen radical absorbance capacity (ORAC), summarized in Table 26. None of the synthesized compounds showed any activity towards BuChE.

A comparison between the activities of **164** and **165** show that the introduction of the carbamate group increases the AChE inhibition by more than 2 folds. The inability of these compounds to inhibit BuChE is a limitation as BuChE is important in regulating the Ach levels during AD. At the same time, the selective inhibition of AChE over BuChE means that these compounds will have less side effects associated with peripheral inhibition of cholinesterase and can also serve as probes for mechanistic investigations. From the *N,N*-dimethyl-4′-aminochalcones, **164**–**177**, the compounds with cyclic amine groups at the carbamate tail, showed better activity than the linear chains. Compound **167** was the most potent among the lot, as it was twice as active as rivastigmine (IC_50_ = 9.94 ± 0.83 µM). Chalcones that were disubstituted with carbamate on ring A at positions 2 and 4, **174**–**177**, had diminished activity as compared to their monosubstituted analog. This could be due to the addition of a bulky group on ring A, which changes the way it interacts with the active site. Some compounds with aminoalkyl groups other than methyl on position 4′ of the chalcone were synthesized. These compounds **178**–**181** were less potent than **167**, which shows that the *N,N*-dimethyl group on position 4′ shows the best activity. Substitution of morpholine ring on position 4′ (compound **181**) caused the activity to decrease by 13-fold when compared with dimethyl and by 6-fold when compared with piperidine. It is possible that the presence of the electron-withdrawing oxygen atom in the ring causes the electron density on nitrogen to decrease, which affects protonation and subsequently the interaction of the compound with the catalytic active site. The results for the antioxidant study on these compounds showed a similar picture where compounds **167**, **170**, and **173** showed better activity. Replacement of the *N,N*-dimethyl group on position 4′ with other aminoalkyl groups resulted in a significant loss of activity, which shows that the methyl substituents on nitrogen are important for antioxidant potency. The study to investigate the effects of these compounds on Aβ aggregation showed that the simple *N,N*-dimethyl-4′-aminochalcone **164** as well as the 4-aminochalcone-rivastigmine hybrids have decent Aβ aggregation inhibition activity. It was concluded that the OH on position 2 of ring A was important for potency. The metal-chelating properties and MAO-A and -B inhibition studies were also discussed in that paper. To sum up, the dimethyl substitutions on position 4′ nitrogen was important for potency and substituting it with other aminoalkyl groups resulted in decreased activity.

**Figure 61:**
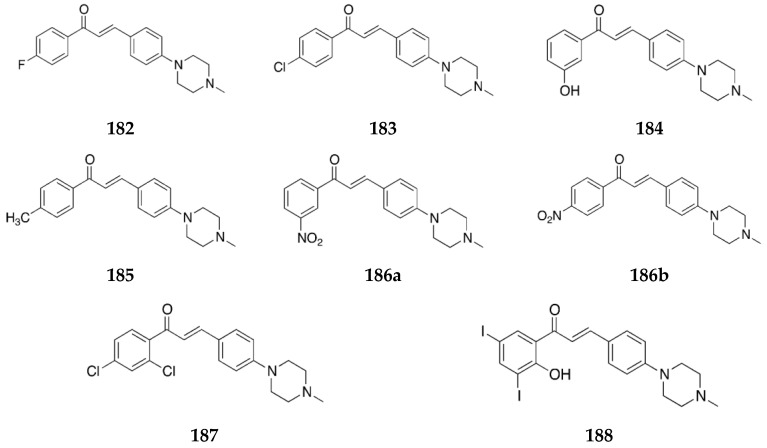
Chemical structure of methyl piperazine-substituted 4′-aminochalcone derivatives.

Noorulhaq et al. [73] reported the synthesis and antimicrobial studies of new chalcone derivatives, including some with methyl piperazine substitutions on position 4′ (Figure 61). 

Compounds **182**–**188** were tested for their antibacterial activity against *Escherichia coli*, *Staphylococcus aureus*, *Salmonella typhi*, and *Bacillus subtillis* and for their antifungal activity against *Aspergillus niger*, *Aspergillus flavus*, *Penicillium chrysogenum*, and *Candida albicans* strains, summarized in Table 27 with dimethyl sulfoxide (DMSO) used as the solvent control.

Penicillin was used as the standard reference drug for antibacterial activity, and nystatin was used as that for antifungal activity. While none of the compounds performed better than the reference drugs, they still showed good activity with a high zone of inhibition values, especially for **187** against *S. aureus* and for **184** against *A. flavus* and *A. niger*. The potency for these compounds can be attributed to the methylated piperazine substitution on position 4′. The increased activity due to piperazine was observed earlier in the discussion of compounds **83**–**88** and **132**–**137**.

**Figure 62:**
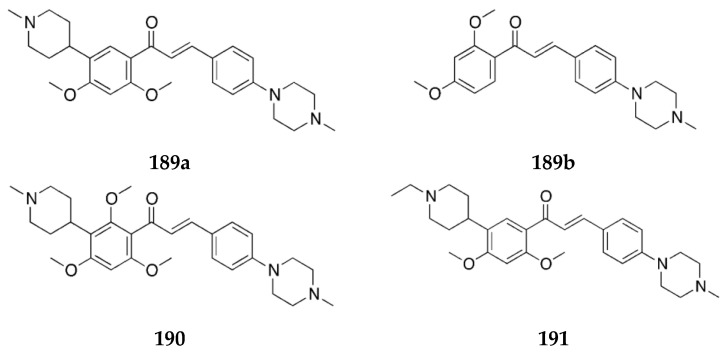
Chemical structure of methylated piperazine-substituted 4′-aminochalcone derivatives.

Apart from all of the other aminochalcones discussed earlier, Liu et al. [66] also synthesized some 4′-aminochalcones (Figure 62) which were tested for their ability to selectively inhibit P-glycoprotein (Pgp; ABCB1) and breast cancer-resistant protein (BCRP; ABCG2).

The activity for the four compounds, **189**–**191**, was not comparable to the reference verapamil or previously discussed compound **89**. Compared to **89**, these compounds have an alkylated piperazine ring at position 4′ and the methoxy substitutions are on ring A instead of ring B, which probably changes the way it interacts with Pgp.

Compound **137** discussed earlier for its antibacterial activity also has an amino group at the 4′- position.

#### 3.2.2. *N*-Alkyl Derivatives of 3′-Aminochalcone

**Figure 63:**
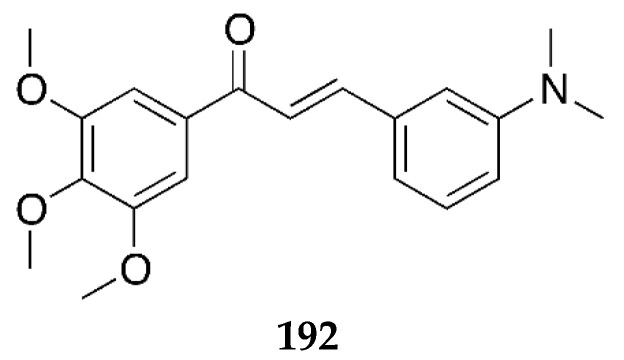
Chemical structure of *N,N*-dimethyl-3′-aminochalcone with methoxy substitutions on ring A.

An *N*-alkyl-3′-aminochalcone (Figure 63) was synthesized and tested by Edwards et al. [61] for its antimitotic activity against HeLa cells. 

The ED value for **192** against HeLa cells was >100 µg/mL. Comparing this value with the *N,N*-dimethyl-4′-aminochalcone analog **143** (ED = 0.015 µg/mL) shows the stark difference in the activity by just changing the position of the dimethylamino group from 4′ to 3′. The *meta* position on ring B is not favorable for antimitotic potency, possibly because the nitrogen on ring B is not in resonance with the enone-system or for sterical reasons. 

**Figure 64:**
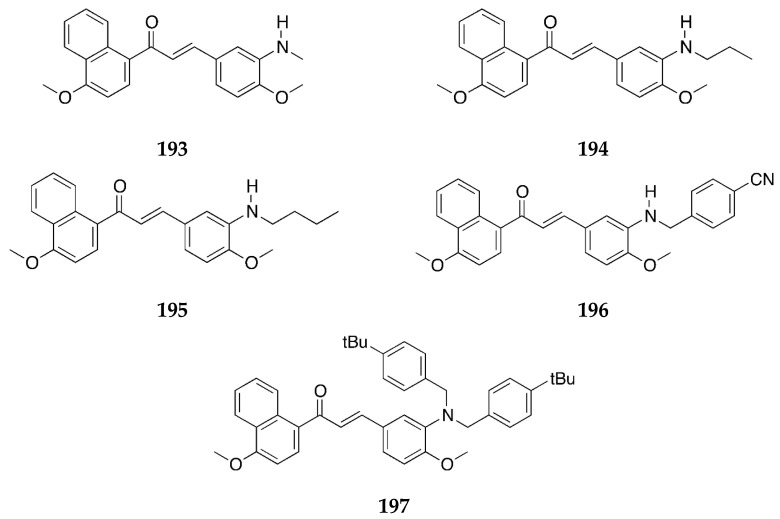
Chemical structure of *N*-alkyl 3′-aminochalcone derivatives.

Wang et al. [63] tested some *N*-alkyl-3′-aminochalcones (Figure 64) for their cytotoxic activities.

The antiproliferative activities **193**–**197** were tested against human cancer cell lines HepG2 and HCT116. Compounds **193**–**197** are 3′-aminochalcones with alkylation on the amino group on ring B. The ring A for these chalcones is a naphthalene ring with methoxy substitution at the 4 position. The IC_50_ values are shown in Table 28.

The in vitro cytotoxic activity for compounds **193**–**197** against human colon carcinoma cells HCT116 and human hepatocellular liver carcinoma cells HepG2 was determined using the MTT assay. The more potent *N*-alkyl compound was **196**. However, **196** was still 17-fold less active than the non-alkylated 3′-aminochalcone **81**, discussed earlier, against the HCT116 cell line (IC_50_ = 0.28 ± 0.06 µM) and 26-fold less active for HepG2 cell line (IC_50_ = 0.19 ± 0.04 µM). 

#### 3.2.3. *N*-Alkyl Derivatives of 2′-Aminochalcone

**Figure 65:**
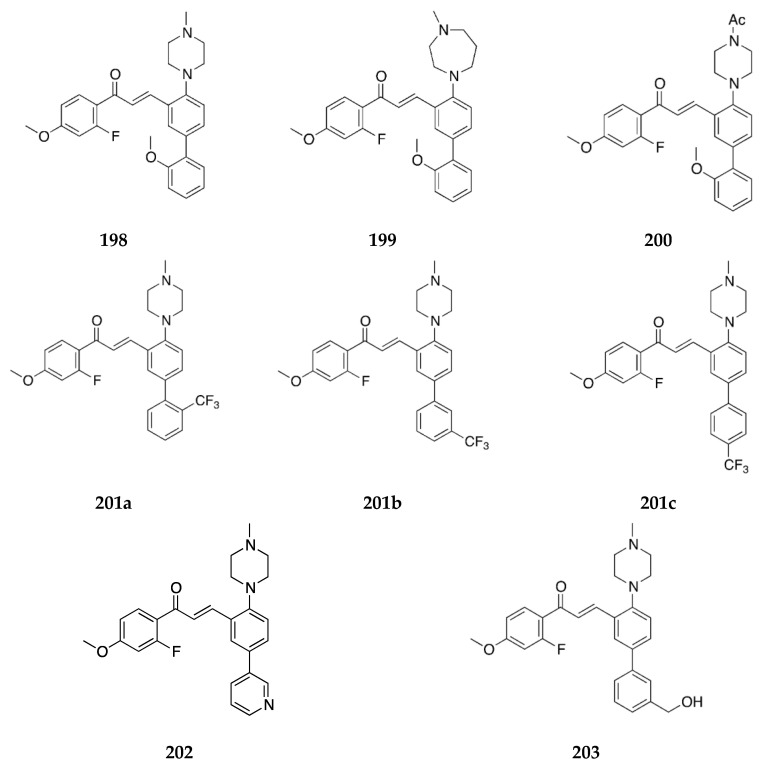
Chemical structures of *N, N*-dialkylated 2′-aminochalcone derivatives.

Very few *N*-alkyl-2′-aminochalcones have been reported in the literature. Nielsen et al. [52] synthesized and reported the antibacterial activity of some *N,N*-dialkyl-2′-aminochalcones (Figure 65).

The antibacterial activities for **198**–**203** against *S. aureus* ATCC33591 (resistant to methicillin), *E. faecium* 17501 (vancomycin-resistant clinical isolate), and *E. faecium* ATCC29212 are shown in Table 29.

Compound **201b** was the most active among all of them. Potency caused by the presence of the piperazine ring on ring B has been discussed earlier for compounds **131**–**135**. In this case, the potency for **201a** and **201b** might be due to the presence of the trifluoromethyl group on the phenyl ring at position 5′. The activity drastically decreases for **202** when the phenyl ring is replaced by pyridyl. 

## 4. *N*-Alkenyl-Aminochalcones

**Figure 66:**
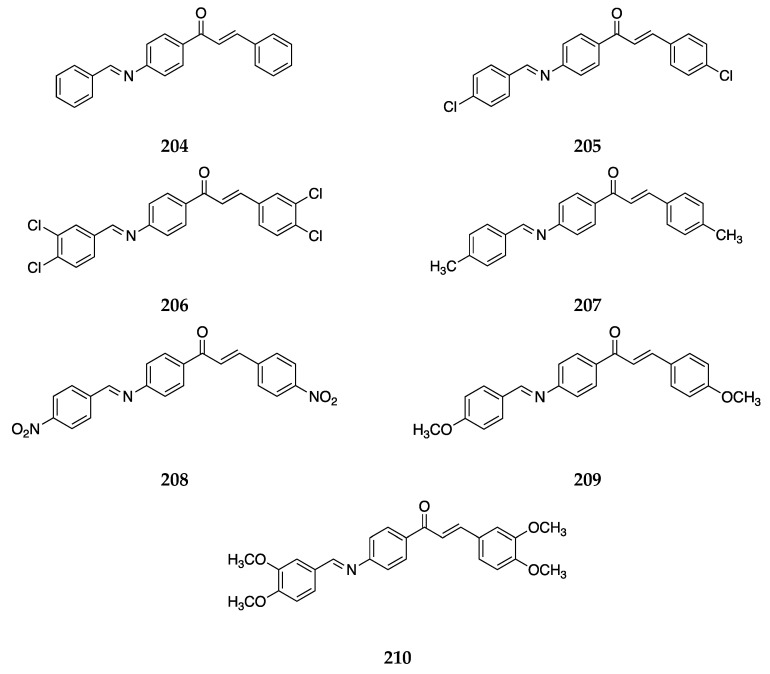
Chemical structures of *N*-alkenylated 4-aminochalcone derivatives.

Dimmock et al. [36] synthesized and tested the cytotoxicity of some *N*-alkenyl-4-aminochalcones (Figure 66).

Some of these compounds were tested for their cytotoxic activity against human Molt 4/C8 and CEM T-lymphocytes as well as murine P388 and L1210 leukemic cells. The IC_50_ values are shown in Table 30.

Since the imine linkage in these molecules is hydrolysable. These compounds tend to present activities similar to the parent compounds. For example, the activities of compound **204**, which is more active in the list, displays activities similar to compound **1**. The chloro-substituted *N*-alkenylated chalcones **205** and **206** had low activity except for against the P388 cell line. The simple mono- and dichloro-substituted-4-aminochalcones, **5a** and **5e**, were almost 50-fold (in some cases, 100-fold) more active against some cell lines (Table 5). This shows that the alkenylation of nitrogen drastically decreased the cytotoxic activity of these chalcones. Similarly, substituting chloro-, methyl-, and methoxy- groups on ring B also led to a decrease in the activity. 

## 5. *N*-Acyl-Aminochalcones 

### 5.1. Substitution on Ring A

#### 5.1.1. *N*-Acyl Derivatives of 4-Aminochalcone

**Figure 67:**
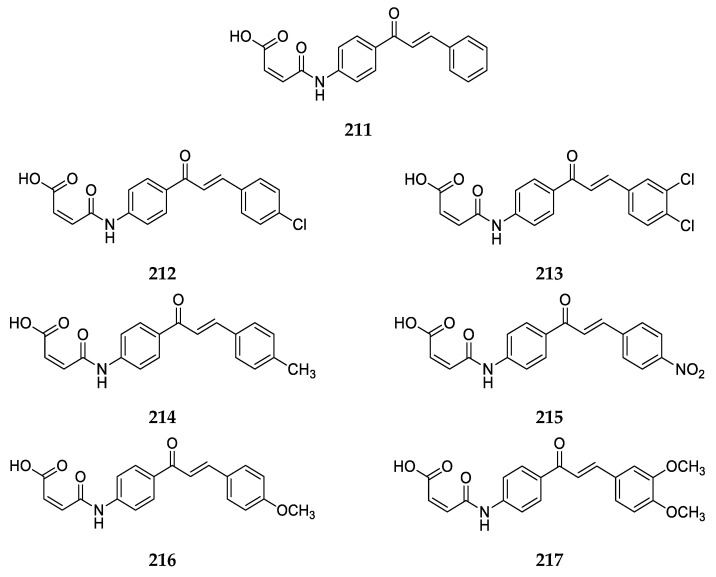
Chemical structures of *N*-4-aminochalcone maleamic acid derivatives with substitution on ring B.

Moving towards *N*-acyl-aminochalcones, there is a reasonable number of such compounds that have been synthesized and tested for biological activity. Different derivatives and types of *N*-acyl compounds have been synthesized by various groups over the years. Dimmock et al. [36] prepared and tested *N*-4-aminochalcone maleamic acids with different substituents on ring B (Figure 67).

The anticancer activity of compounds **211**–**217** against human cancer cell lines in terms of the concentration of compound required for 50% inhibition (IC_50_) is summarized in Table 31.

The chemistry of the *N*-acyl-aminochalcones varies from the previously discussed aminochalcones due to the difference in reactivity of the nitrogen-containing groups. In general, *N*-acylation of the primary amine group of the compounds **1**, **5a**, **5e**, **2**, **8**, **3a**, and **3h** to give compounds **211**–**217** proved to be detrimental to the potencies against the cancer cell lines. It was hypothesized to be due to the polar carboxyl group at the tail, but it would also be a result of the involvement of the lone pair of the *N*-atom in the newly formed amide bond with the acyl group. The results reveal a decent potency of **215** against murine P388 cells, which sets it apart from other compounds in the series. The potency of **215** is greater than the simple -NH_2_-substituted 4-aminochalcone **8** unlike all other comparisons between the two classes of compounds. Compounds **211**–**214**, **216**, and **217** are not promising anticancer agents, evident from their high IC_50_ values. In contrast, when compound **213** was tested against another cancer cell line, RPMI-8226, its IC_50_ value was found to be 0.112 µM [36]. This value demonstrates the high potency of **213** against this cell line and that it is 53 times more cytotoxic than melphalan. Compounds **211** and **214** were also tested for antifungal activity, but they showed no inhibition against *Aspergillus fumigatus* and *Candida albicans*. The activity for *N*-acyl compounds **211**–**217** was consistently lower than the *N*-alkenylated analogs **204**–**207** and **209**. Between the unsubstituted compounds **204** and **211**, activity for the *N*-alkenyl-4-aminochalcone was almost 20-fold higher than the *N*-acyl-4-aminochalcone against the Molt 4/C8, CEM, and L1210 cell lines.

**Figure 68:**
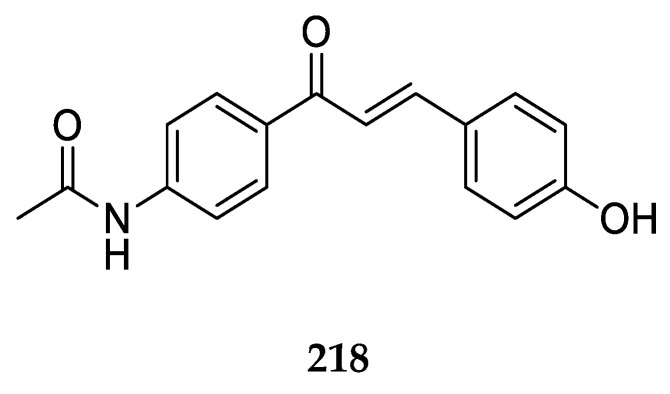
Chemical structure of *N*-acetyl-4-aminochalcone derivative with substitution on ring B.

A simple derivative of *N*-acyl-4-aminochalcone with hydroxy substitution on ring B was synthesized by Kang et al. [48] (Figure 68). 

The inhibitory effect of compound **218** was investigated against the action of β-secretase (BACE1), an enzyme strongly correlated with the onset of Alzheimer’s disease. However, the IC_50_ value of **218** was >200 µM, which was significantly higher than the other aminochalcone derivatives tested. The significant loss in potency was attributed to the acylation at nitrogen in compound **218**. 

**Figure 69:**
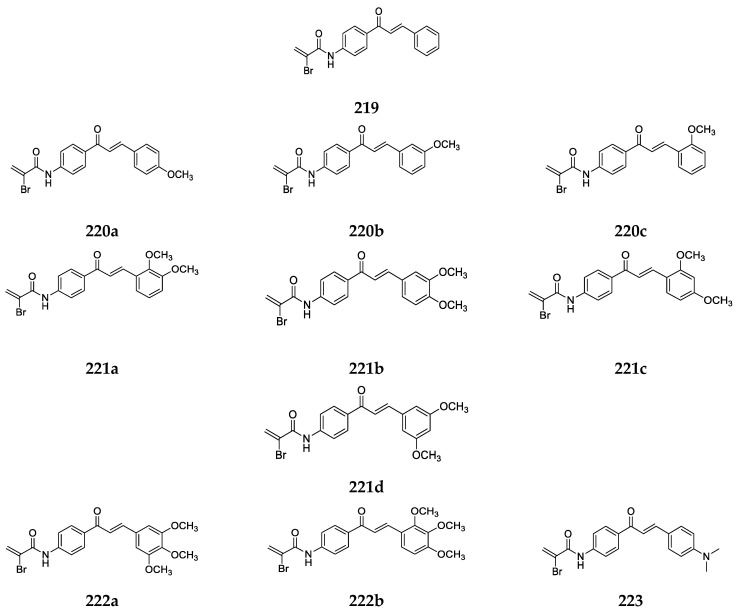
Chemical structure of α-bromoacryloylamido chalcone derivatives with substitution on ring B.

Next, a series of α-bromoacryloylamido chalcones was prepared and tested for antiproliferative activity against several cancer cell lines by Romagnoli et al. [37] (Figure 69).

The anticancer activity of compounds **219**–**223** was tested against six cancer cell lines, which are summarized in Table 32.

Overall, compound **222b** displayed the highest activity against the cell lines and is the most promising lead molecule of the series. Most of the compounds of this series have markedly better activity than the compounds **211**–**217**, with significantly lower IC_50_ values. The results of the investigation suggested that the position of the bromoacrylamide moiety on the chalcone is important because the activity of compounds **219**–**223** was found to be better than the compounds with the bromoacrylamide group on ring B tested in the same study [37], which will be discussed later. The compounds **219**–**223** show very similar activity that did not change much with the changing substitutions on ring B. Thus, the pharmacophore is likely to be the bromoacryloylamido moiety on ring A.

**Figure 70:**
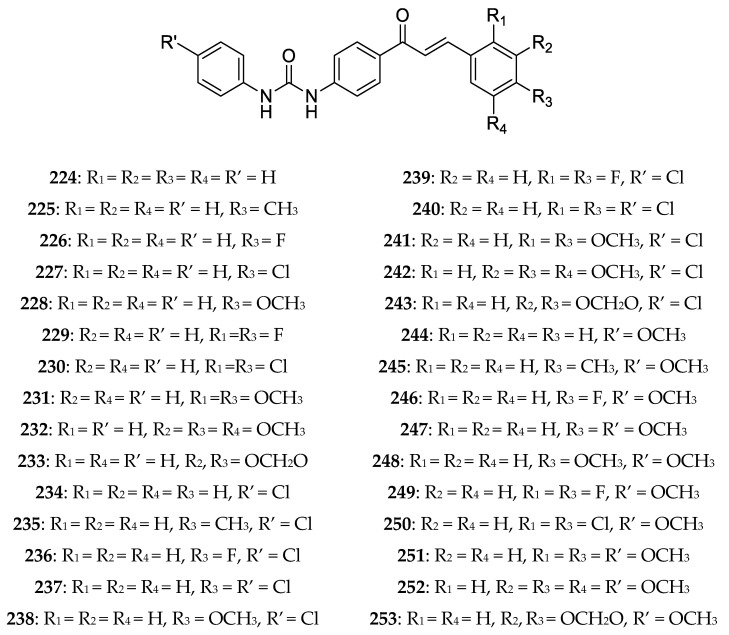
Chemical structure and side chains of *N*-acyl-4-aminochalcone derivatives with substitution on ring B.

**Figure 71:**
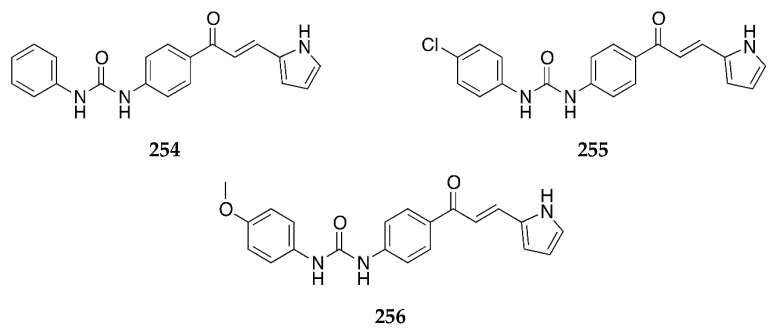
Chemical structure and side chains of *N*-acyl-4-aminochalcone derivatives with substitution on ring B.

Dominguez et al. [74] synthesized a long series of *N*-acyl-aminochalcones, with two types of compounds (Figure 70 and Figure 71) and tested their antimalarial potential specifically as inhibitors of in vitro development of a chloroquine-resistant strain of *Plasmodium falciparum*, the activity of the cysteine protease falcipain-2, and in vitro globin hydrolysis.

Among *N*-arylurea-aminochalcones **224**–**256,** compounds **234**, **237**, **239**, **240**, **242**, **253**, **255**, and **256** showed some decent ability to inhibit parasite development at or below 10 µM. These compounds were therefore tested against falcipain-2 in terms of their IC_50_ values shown in Table 33.

**Figure 72:**
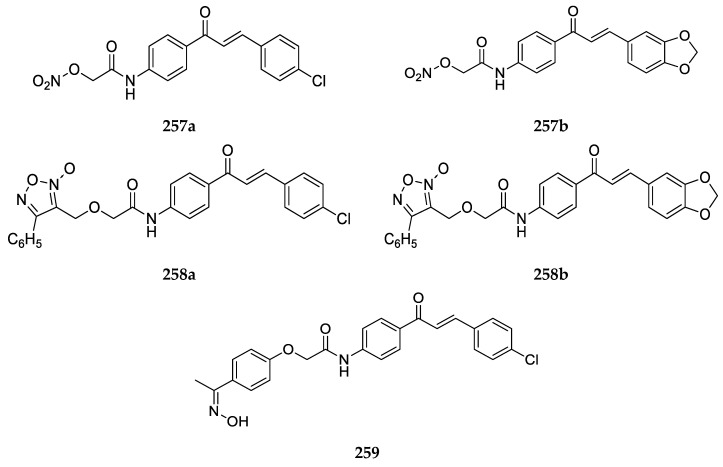
Chemical structure of *N*-acyl-4-aminochalcone derivatives with substitution on ring B.

The activity of compounds in Table 33 was impressive, with compound **242** being the most active against recombinant falcipain-2. Furthermore, compounds that inhibited parasite development were evaluated for the inhibition of globin hydrolysis. Compound **255** was the most active, with 98% inhibition of hemoglobin degradation at 5 µM. Dominguez et al. [74] also tested the ability of the compounds to inhibit hemozoin formation, which showed that compound **255** inhibits hemozoin with IC_50_ = 0.73 mM that is more potent than chloroquine (IC_50_ = 1.33 mM). The activity of **255** was associated with the presence of 2-pyrrolyl, which replaced the aromatic ring. This structure has a nitrogen-containing heterocycle that resembles the nitrogen of chloroquine. Some *N*-acyl, nitric oxide-donating, 4-aminochalcone derivatives (Figure 72) were prepared by Mourad et al. [75] and tested against a wide variety of cancer cell lines for cytotoxic activity. 

The cytotoxicity of **257a**–**259** was tested on sixty different leukemia, lung, renal, breast, colon, melanoma, prostate, ovarian, and skin cancer cell lines and reported as the concentration required for 50% maximal inhibition of cell proliferation for cytostatic agents (GI_50_ values). Among the five compounds, the activities of **257a**–**b** were remarkable against many cell lines. Compound **257a** had GI_50_ values in the range 0.30 µM to 15.4 µM for the sixty cell lines, while **257b** displayed similarly potent activity, especially against colon cancer cell lines. The furoxan-substituted compounds **258a** and **258b** did not show significant cell growth inhibition, while **259** exhibited moderate cell growth inhibition against melanoma UACC-62 (cell growth promotion = 37.53% and inhibition = 62.47%). It was hypothesized that the nature and size of the linker might have contributed to the decreased activity of compounds **258a**–**259**. Overall, the results revealed that the selected NO-donating compounds **257a** and **257b** exhibited strikingly potent cytotoxic activity against various cancer cell lines, where the maximum inhibitory activity was achieved with nitrate ester **257a**. Finally, these novel synthesized NO-donating hybrids denote a significant strategy against cancer that needs further biological studies [75].

**Figure 73:**
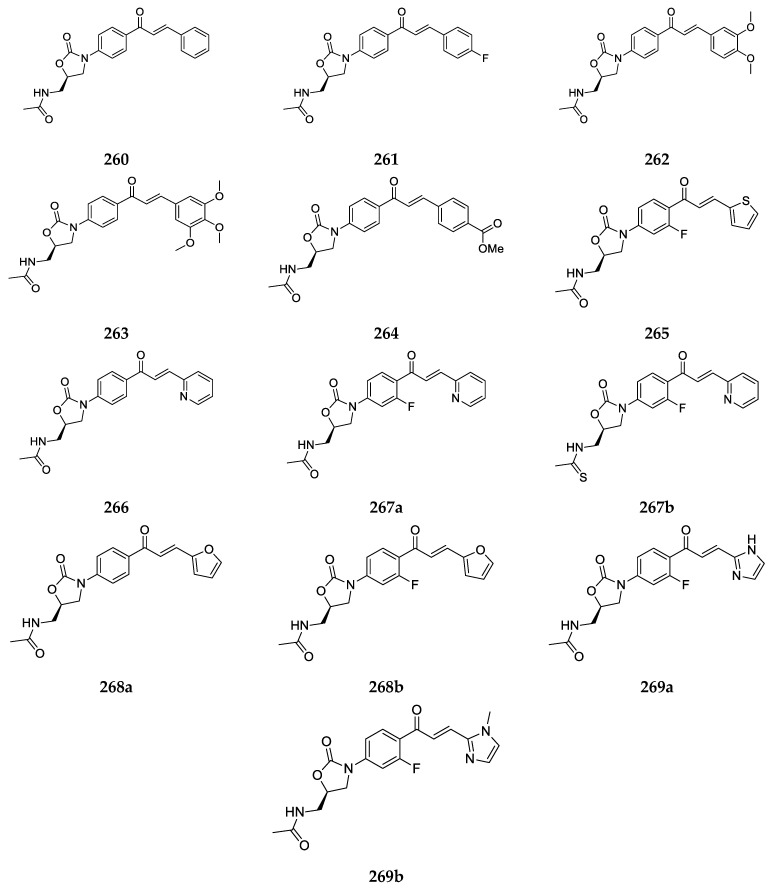
Chemical structure of 2-oxo-oxazolidin-3-yl-chalcone derivatives with substitution on ring A.

Another series of 2-oxo-oxazolidin-3-yl-chalcones was reported by Selvakumar et al. [76] (Figure 73).

The antibacterial activities of compounds **260**–**269b** were tested against six bacterial strains and reported as MIC values, shown in Table 34.

Among compounds **260**–**269b**, only compound **267b** had antibacterial activity equal to or better than the standard drugs Linezolid and Vancomycin against the bacterial strains tested. Compounds **266**–**267a** and **268a**–**269b** showed moderate activity, and it was observed that, when the fluorine atom was added to ring B of **266** and **268a** to give **267a** and **268b**, the antibacterial activity improved considerably. However, the addition of thiophene and imidazole heterocycles resulted in compounds **265**, **269a** and **269b** that were poor in activity. Interestingly, a change from oxygen to sulfur that led to **267b** from **267a** engendered an activity by **267b** many folds better than its acetamide analog **267a**. This gives a promising lead molecule for better antibacterial agents [76].

#### 5.1.2. *N*-Acyl Derivatives of 3-Aminochalcone

**Figure 74:**
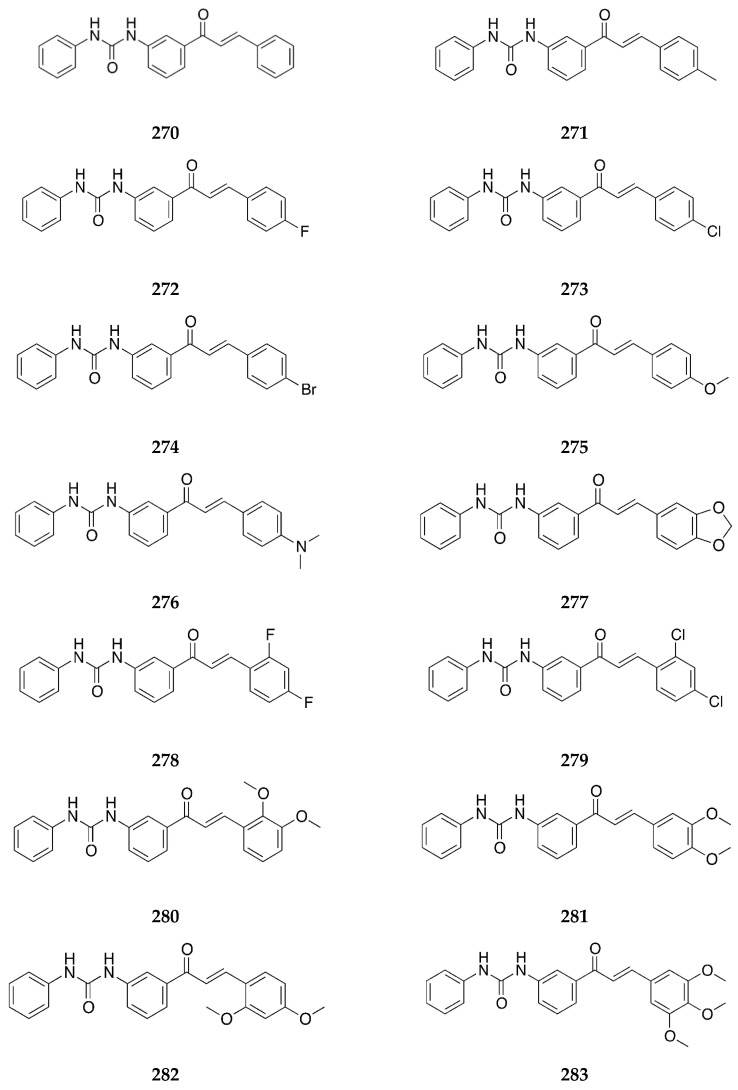
Chemical structure of *N*-acyl-3-aminochalcone derivatives with substitution on ring B.

Proceeding the discussion of *N*-acyl-aminochalcones, some research groups have prepared 3-aminochalcones, with acylation on nitrogen, and tested them for different biological activities. In addition to 4-aminochalcones, Dominguez et al. [74] have also prepared a series of *N*-arylurea-3-aminochalcones with different substituents on ring B (Figure 74).

The antimalarial activity of compounds **270**–**283** in the form of inhibition of in vitro development of a chloroquine-resistant strain of *Plasmodium falciparum*, of the activity of the cysteine protease falcipain-2, and of in vitro globin hydrolysis was tested following synthesis. Initially, fluorescence-activated cell sorting (FACS) analysis revealed their ability to inhibit parasite development. Selected compounds showed activity including **271** (IC_50_ = 2.74 µM), **274** (IC_50_ = 2.10 µM), **279** (IC_50_ = 2.14 µM), and **283** (IC_50_ = 1.76 µM). These selected compounds were then subject to tests for inhibition of globin hydrolysis and only showed mild inhibition of hemoglobin degradation (51–75%), with **279** and **283** performing relatively better than the other two compounds. The authors also thought that *para* and *meta* positions in the urea-substituted phenyl ring play an important role in their antimalarial activity, as could be observed for **279** and **283**. They concluded that derivative **283** had the best antimalarial properties while its mechanism of action did not appear to be related to hemoglobin hydrolysis or heme detoxification, and compared against compound **255**, at the same concentration, **283** was better at stopping the development of *P. falciparum* in culture (IC_50_ = 1.76 µM), but further studies are necessary [74].

**Figure 75:**
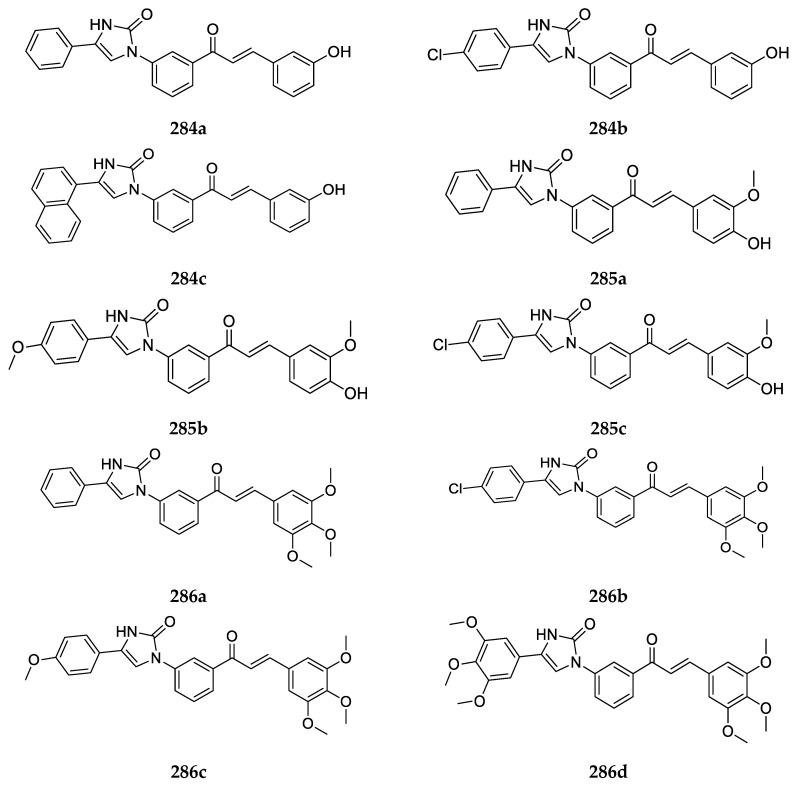
Chemical structure of 2-oxo-imidazol-1-yl-chalcone derivatives with substitution on ring B.

Several interesting 2-oxo-imidazol-1-yl-chalcone derivatives were synthesized and tested for antiproliferative activity by Kamal et al. [77] (Figure 75). 

Kamal et al. tested cell growth inhibition by compounds **284a**–**286d** against fifty-three different cancer cell lines from nine cancer types: leukemia, lung, colon, CNS, melanoma, ovarian, renal, prostate, and breast cancer. Most of the compounds displayed GI_50_ values in the range of 0.23 µM to 35.2 µM for the fifty-three cell lines. Three of the compounds, **285a**–**c**, showed reproducible results and antitumor activity (GI_50_ values) in the ranges of 1.35–6.81, 1.35–13.9, and 1.26–10.5 µM, respectively. FACS studies revealed significant cell-cycle arrest in the G2/M phase shown by **285a** and **285c** with **285a** [77].

### 5.2. Substitution on Ring B

#### 5.2.1. *N*-Acyl Derivatives of 4′-Aminochalcone 

**Figure 76:**
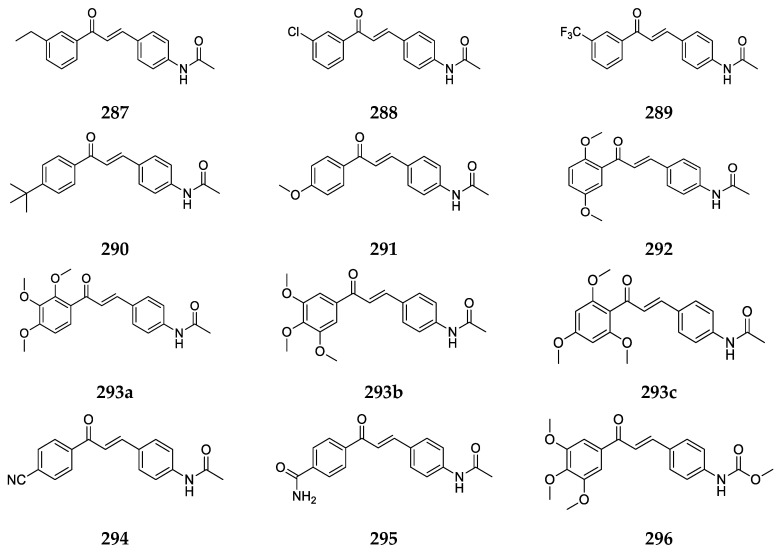
Chemical structure of *N*-acyl-4′-aminochalcone derivatives with substitution on ring A.

Now, we bring our attention to the compounds having an *N*-acyl group on ring B. The simplest series of such 4′-aminochalcones, **287**–**296**, was prepared by Edwards et al. [61] (Figure 76). Compounds **287**–**295** are *N*-acetyl derivatives. The compounds were synthesized by a base-catalyzed Claisen-Schmidt condensation of appropriately substituted aldehydes and ketones. 

Earlier, we have discussed -NH_2_-substituted and *N*-alkyl-aminochalcones from the same investigation. Similarly, the potential of compounds **287**–**296** as antimitotic agents was tested against HeLa cells. The activity of these compounds was reported as the equivalent dose (ED), which is the concentration of the compound found to be equivalent to 0.05 µg/mL colchicine. Table 35 represents the ED value of compounds **287**–**296** over 6 h [61]. Apart from the acetylation on nitrogen at position 4′, compound **62c** has the same structure as **293b**. Compound **62c** has also been added as a reference compound in Table 35. 

Compound **293b** acted as an initial lead for the other compounds. *Meta*-substituted derivatives **287**–**289** had diminished activity with high ED values. Compounds **293a**–**c** were equipotent in activity and showed the minimum ED values in this compound series. Overall, compound **290** was the most potent among these as an antimitotic agent [61]. Comparing **293b** with **62c**, it can be seen that the potency decreases with acetylation. 

**Figure 77:**
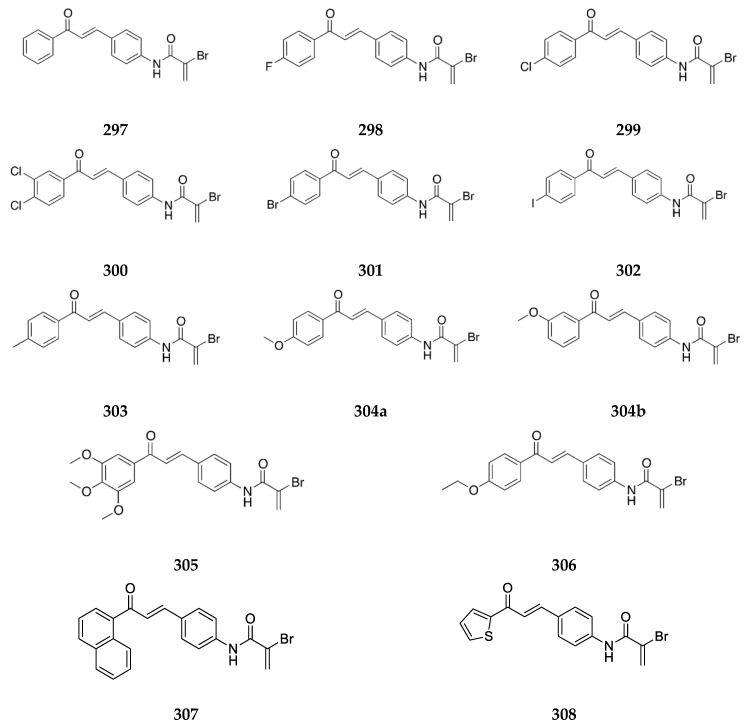
Chemical structure of α-bromoacryloylamido-chalcone derivatives.

Romagnoli et al. [37] also synthesized a novel series of α-bromoacryloylamido-chalcones and evaluated them for antiproliferative activity against five cancer cell lines. These compounds included phenyl-containing chalcones and chalcones with naphthalene and a heterocycle instead of the phenyl ring B (Figure 77).

The results of assays testing the anticancer activity of compounds **297**–**308** are summarized in Table 36.

Compounds **297**–**308** were designed to assess the structure–activity relationship (SAR) due to different substitutions, both electron-releasing and electron-withdrawing groups, and their positions on ring A. Due to the presence of an α-bromoacryloyl moiety, these compounds were significantly more potent in comparison to the 4′-aminochalcone analog having a -NH_2_ group, tested in the same study and shown in Table 18. Compounds **306** and **308** were the more active compounds in the series, which appeared to be due to the thiophene group in the case of **308**. Compound **306** appeared to induce apoptosis mediated by the involvement of mitochondria and by the activation of caspase-3. Moreover, a trend was observed for the halogen-substituted compounds **298**, **299**, **301**, and **302**, where activity increased in the following order: Br (**301**) > Cl (**299**), F (**298**) > I (**302**). The addition of another chlorine atom to **299**, to give the *meta*, *para*-dichloro derivative **300**, resulted in about a twofold decrease in the activity. Likewise, a two-fold decrease in activity was observed by shifting the methoxy group from the *para* (**304a**) to the *meta* position (**304b**) on ring A. On the whole, it was found that the introduction of either electron-releasing (ERG) or electron-withdrawing (EWG) substituents (derivatives **298**–**306**) enhanced antiproliferative activity juxtaposed with the unsubstituted analog **297**, and the distinctions between them were not prominent but the greatest improvement in activity seemed to occur with the bulkier substituents **301** and **306**. Moreover, these *N*-acyl compounds showed far greater activity than the simple -NH_2_-substituted chalcones tested by Romagnoli et al. [37]. Thus, it can be concluded that the potency of these compounds is due to the presence of bromoacryloylamido moiety.

**Figure 78:**
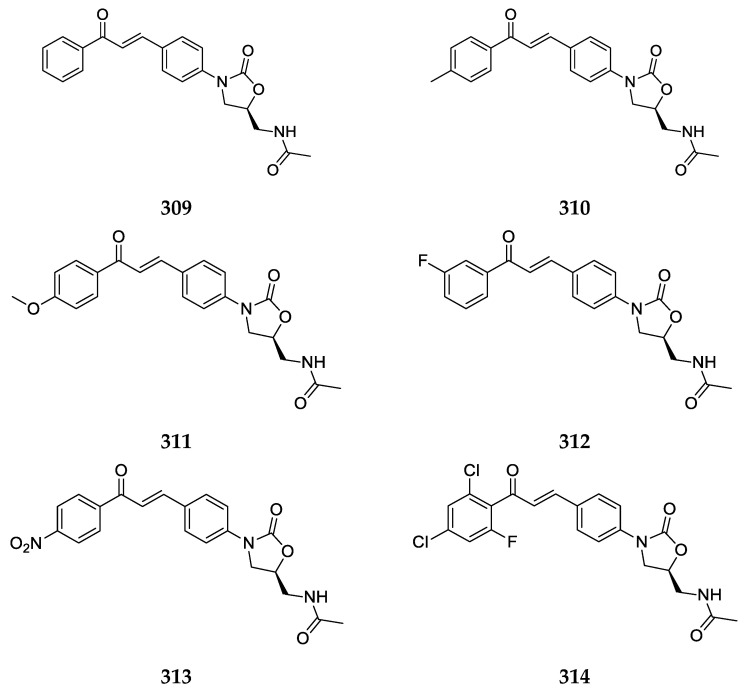
Chemical structure of *N*-acyl-4′-aminochalcone derivatives with substitution on ring A.

Apart from ring A-substituted 4-aminochalcones **260**–**269b** discussed earlier, Selvakumar et al. [76] also prepared and evaluated ring B-substituted *N*-acyl-4′-aminochalcones (Figure 78).

All compounds were made via the usual aldol condensation, and compounds **309**, **312**, and **313** were obtained as inseparable mixture of *E* and *Z* isomers. Compounds **310** and **311** had electron-donating groups, while **312**–**314** had electron-withdrawing groups. The inhibitory effects of these compounds against *Staphylococcus aureus* ATCC 33591 (methicillin-resistant), *Staphylococcus aureus* ATCC 49951, *Staphylococcus aureus* ATCC29213, *Enterococcus faecalis* ATCC29212 (vancomycin sensitive), *Enterococcus faecalis* NCTC 12201 (vancomycin-resistant), and *Enterococcus faecium* ATCC 12202 (vancomycin-resistant) were tested. However, all of them displayed trace or no antibacterial activity with MIC values ≥32 µg/mL for all strains tested. The antibacterial activity of structural analog **260**–**269b** (Table 34) was much better than this series of *N*-acyl-aminochalcones. Therefore, we can deduce that the location of the *N*-acyl-amino group matters for antibacterial activity and that the acylated amino group on ring A rather than B leads to better potency [76].

#### 5.2.2. *N*-Acyl Derivatives of 3′-Aminochalcone 

**Figure 79:**
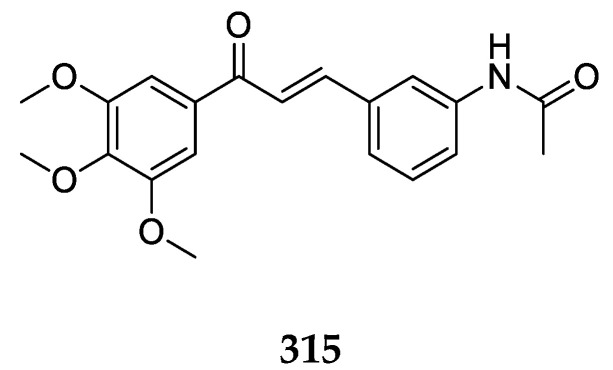
Chemical structure of *N*-actyl-3′-aminochalcone derivatives with substitution on ring A.

In addition to the *N*-acyl- and *N*-acetyl-amino compounds **287**–**296**, Edwards et al. [61] also prepared a single *N*-acetyl-3′-aminochalcone (Figure 79) and similarly tested it for antimitotic activity against HeLa cells.

The ED of compound **315** was >100 µg/mL. This activity was lower than most of the 4′-aminochalcone analogs in the series tested, including compound **293b**. The *meta* substitution appeared to be the cause of the drop in the antimitotic activity compared to **293b**. Hence, **315** neither is a promising anticancer agent nor leads to further developments [61].

**Figure 80:**
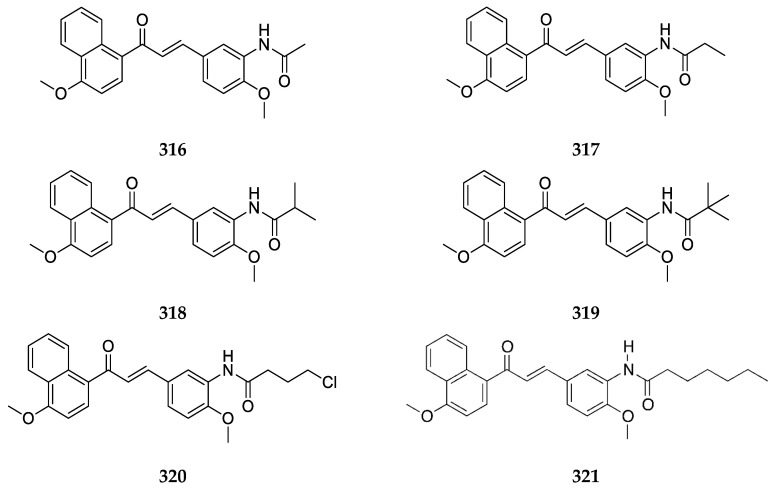
Chemical structure of *N*-acyl-3′-aminochalcone derivatives with substitution on rings A and B.

Finally, another set of *N*-acyl-3′aminochalcone derivatives with substitutions on rings A and B was made and reported by Wang et al. [63] (Figure 80) and evaluated for their antiproliferative activity against HepG2 and HCT116 human cancer cell lines. This investigation also reported the activity for some *N*-alkylated-3′-aminochalcones **193**–**197** discussed earlier.

The novel compounds **316**–**321** were analyzed for their in vitro cytotoxicity against human colon carcinoma cells (HCT116) and human hepatocellular liver carcinoma cells (HepG2) using 3-(4,5-dimethylthiazol-2-yl)-2,5-diphenyltetrazolium bromide (MTT) assay. The IC_50_ values of the compounds are shown in Table 37. Compound **193**, the non-acetylated 3′-aminochalcone analogue of **316**, was also added for comparison.

Compounds **316**–**318** exhibited good cytotoxic activity; however, these activities were still less than that of -NH_2_-substituted 3′-aminochalcone **81**. The activities of compounds **319**–**321** were poor against the cancer cell lines tested. Overall, it was observed that the introduction of normal or branched chain alkyl acyl substituents **316**–**321** at the amino group of ring B resulted in a decrease in the antiproliferative activity of the aminochalcone; therefore, further studies for appropriate substituents are needed to improve anticancer cancer activity. Compared to the *N*-alkylated-3′-aminochalcones **193**–**197**, **316**–**318** exhibited much better activity, which shows that, in this case, acylation on the amino group leads to higher potency as compared to alkylation [63].

## 6. *N*-Sulfonyl-Aminochalcones

### 6.1. N-Sulfonyl Derivatives of 4-Aminochalocone

**Figure 81:**
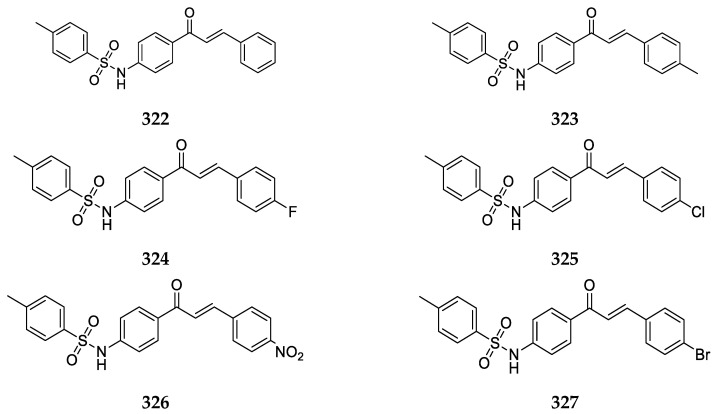
Chemical structure of sulfonamide-4-aminochalcone derivatives with substitution on ring B.

The next class of aminochalcones contains compounds that have a sulfonamide group and its derivatives with different substituents on ring B. Ghorab et al. [78] synthesized a novel series of sulfonamide derivatives of aminochalcones by the Claisen-Schmidt condensation between the appropriate acetophenone derivatives and aromatic aldehydes in the presence of potassium hydroxide in ethanol (Figure 81).

Compounds **322**–**327** were tested for in vitro anticancer activity against human liver cancer cell line HEPG-2. The results are presented in Table 38 in the form of concentration required for 50% inhibition (IC_50_ values).

Among the compounds tested, **322** showed no antiproliferative activity against HEPG-2. Although **326** performed relatively better than **327**, both were among relatively more potent compounds in the series. Compounds **326** and **327** were also screened against the cell line after exposure to γ-radiation, and their IC_50_ values were reduced to 20.5 µM and 26.8 µM, respectively [78].

**Figure 82:**
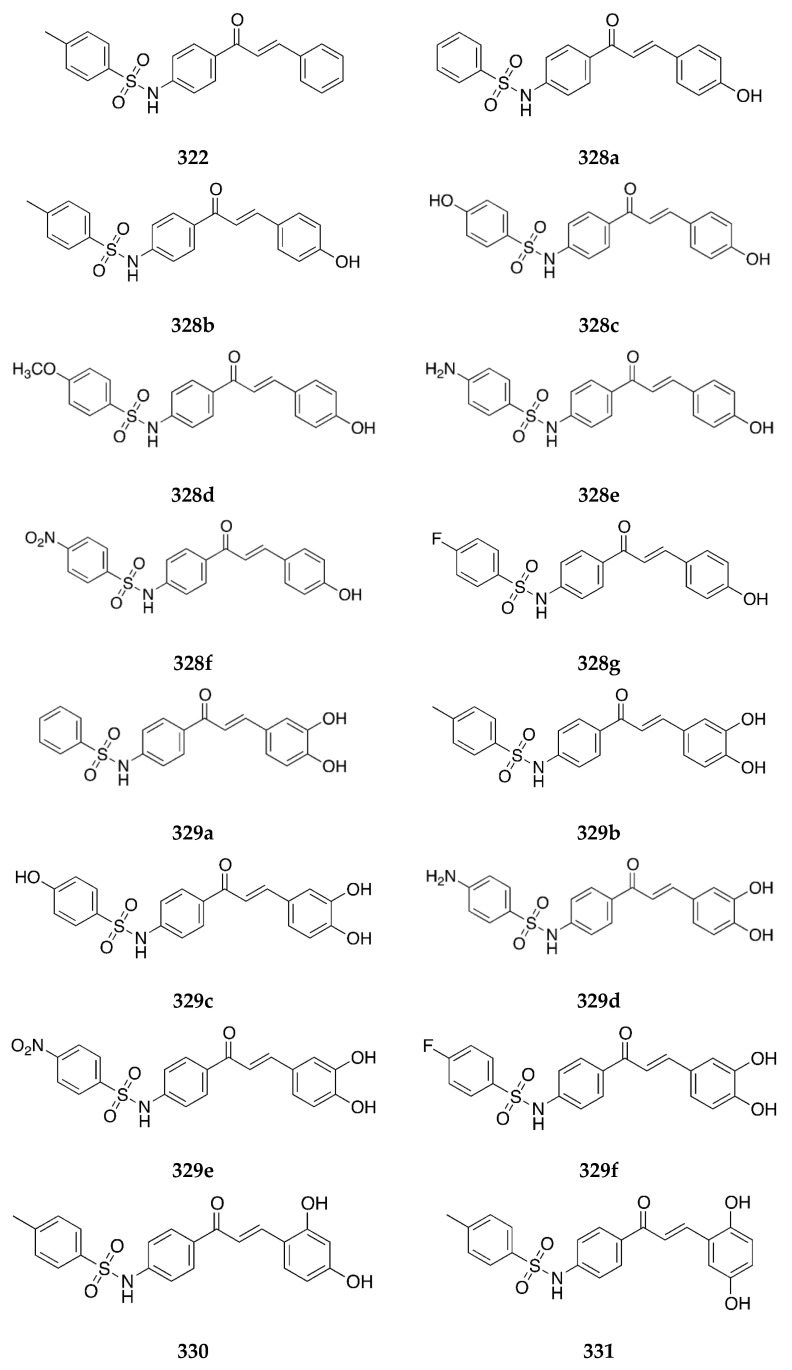
Chemical structure of *N*-sulfonyl-4-aminochalcone derivatives with substitution on ring B.

Kang and coworkers [48] also prepared an elaborate series of sulfonated-4-aminochalcones (Figure 82) and tested their activities against the β-secretase (BACE1), an enzyme linked to the onset of Alzheimer’s disease.

Table 39 summarizes the IC_50_ values of compounds **322** and **328a**–**331** against the action of β-secretase (BACE1).

Sulfonamide aminochalcones **328b** and **329a**–**d** displayed remarkable inhibitory effects against the β-secretase enzyme, with the lowest IC_50_ value shown by **329b**. Compound **329b** was also found to be 70-fold more effective than the corresponding aminochalcone bearing an unsubstituted amine **4b** (IC_50_ = 17.7 μM). Similarly, **328b** showed a markedly enhanced activity in comparison to **4a** (IC_50_ = 48.2 μM). The inhibition activity was observed to be significantly affected by the position and number of hydroxyl groups on ring B. This is also evident from the relatively poor activity of compound **322,** which does not carry a hydroxy group on ring B. A generally better inhibition was observed when the benzenesulfonyl ring had an electron-donating group in the *para* position; however, the electron-donating potential and size of the substitution also impacted the activity, where smaller groups were better than larger ones. Following this trend, the strongly electron-withdrawing group bearing compounds **328f**–**g** and **329e** were the least active inhibitors of BACE1. In addition, the **329**-series, bearing a 3,4-dihydroxy group ring B was in general more potent than the corresponding 4-hydroxy derivatives (**328a**–**g**). Therefore, these *N*-sulfonyl-aminochalcones act as valuable lead compounds and are useful in further experimentation and optimization [48].

Compounds **328b** and **329b** were also synthesized by Kim et al. [79] and tested for inhibition of trans-sialidase from *Trypanosoma cruzi* (TcTS), a parasite that causes Chagas disease—a disease with a mortality rate as high as 85% and results in interruption of host immune responses, heart disease, and malformation of the intestines. IC_50_ values of **328b** and **329b** were 73 μM and 0.9 μM, respectively. It was suggested that the dihydroxy aromatic moiety present in **329b** was a major contributor to the inhibition of TcTS activity.

Seo et al. [47] prepared compounds **328b** and **329b** along with other aminochalcones and tested their glycosidase inhibitory activities against α-glucosidase, α-amylase, and β-amylase. Compound **329b** proved to be the better inhibitor against α-glucosidase with IC_50_ = 0.4 μM. These compounds showed noncompetitive inhibition against the enzymes. Compound **328b** also showed an impressively low IC_50_ value of 0.98 μM promising good inhibition against α-glucosidase. Both the compounds displayed mediocre inhibition against α and β-amylases, with IC_50_ values in the range 65 μM to 247.3 μM [47].

In another study, Seo et al. [49] prepared compounds **328a**–**c** and **328e**–**g** and evaluated them for tyrosinase activity and depigmenting ability. The results of the investigation are presented in Table 40. 

It was found that the 4-hydroxy substitution on ring B was central to the competitive tyrosinase inhibition. Although compounds **328a**–**c** and **328e**–**g** showed slightly lower activities than some other chalcones, the more active compound among these was **328f** for tyrosinase inhibition and **328b** for melanin formation, respectively. The inhibitor constant, K_i_, which is the concentration required to produce half-maximum inhibition, indicating the potency of the inhibitor, was also determined for tyrosinase activity, and as predicted by its IC_50_ value, **328f** had the lower K_i_ value [49].

**Figure 83:**
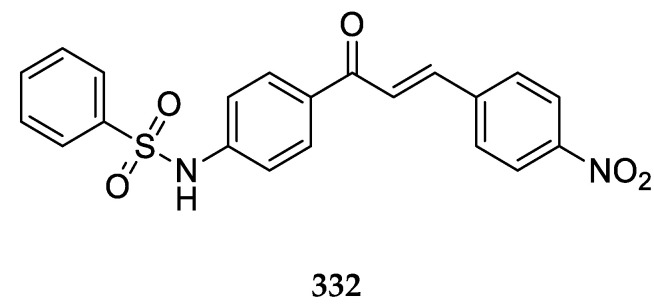
Chemical structure of a sulfonamide-4-aminochalcone derivative with substitution on ring B.

Moving on, Silva et al. [80] prepared a sulfonamide chalcone derivative with a nitro substituent on ring B (Figure 83).

Then, the genotoxic, cytotoxic, antigenotoxic, and anticytotoxic activities of **332** were assessed using the *Salmonella typhimurium* reverse mutation test (Ames test) and the mouse bone marrow micronucleus test. The Ames test results revealed the potential mutagenic and anti-mutagenic effects of compound **332** at relatively higher doses, whereas the mouse bone marrow micronucleus test uncovered the genotoxic and cytotoxic effects of compound **332,** later attributed to the presence of the nitro group. Overall, the results presented a moderate profile of the compound [80].

**Figure 84:**
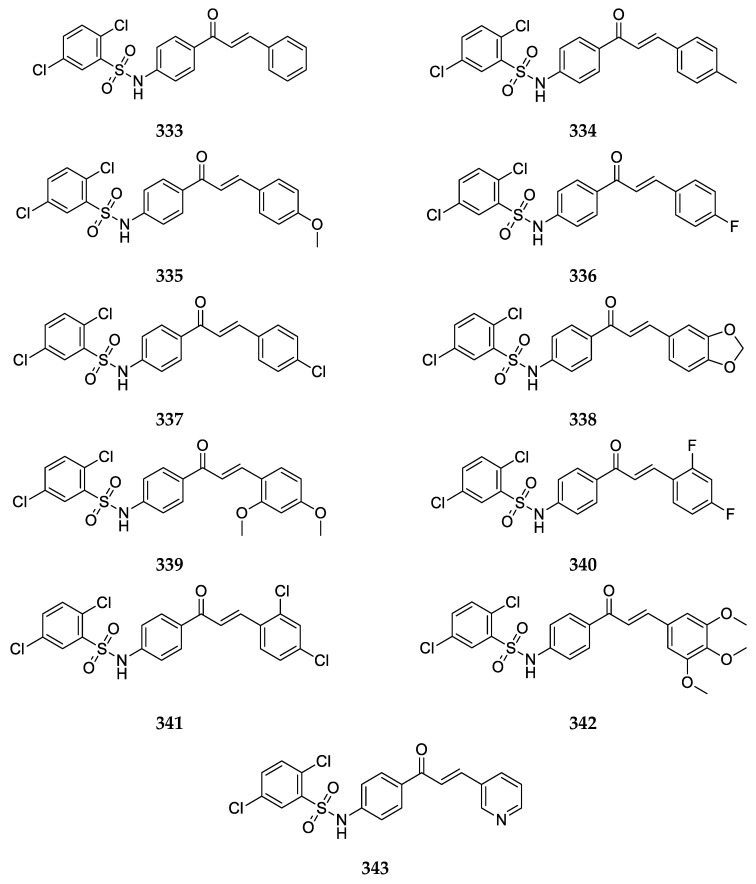
Chemical structure of sulfonamide-4-aminochalcone derivatives with substitution on ring B.

Lastly, Dominguez et al. [74] reported a series of sulfonamide-chalcone derivatives with different substituents on ring B (Figure 84).

This group evaluated the antimalarial activity of sulfonamide chalcones **333**–**343** against cultured *Plasmodium falciparum* parasites and their effect on inhibition of β-hematin formation in terms of their IC_50_ values shown in Table 41.

Compounds **333**–**337** and **341** showed no measurable activity against the β-hematin formation. On the other hand, compounds **340**, **342**, and **343** inhibited β-hematin formation better than chloroquine (IC_50_ = 1.33 µM). By a general comparison of **339** and **342**, the more active compound in the series, we can observe that, upon going from 2,4-dihydroxy substitution to 3,4,5-trihydroxy substitution on ring B, the inhibitory activity improved significantly. On the other hand, there was no significant inhibition of the development of cultured parasites except for compound **334,** which was potent but without any correlation with the inhibition of heme formation. Moreover, the presence of the pyridinyl moiety in **343** seemed to cause considerable improvement in antimalarial activity, which might be due to its resemblance with chloroquine [74].

### 6.2. N-Sulfonyl Derivatives of 3-Aminochalocone 

**Figure 85:**
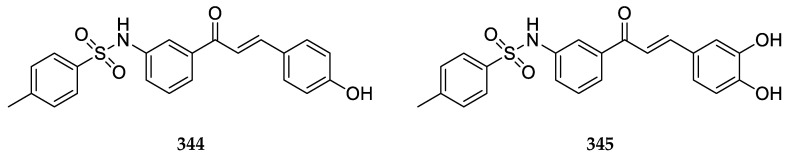
Chemical structure of sulfonamide-3-aminochalcone derivatives with substitution on ring B.

A pair of sulfonated 3-aminochalcone derivatives hydroxylated on ring B have also been made and reported by Seo et al. [47] and Kim et al. [79], shown in Figure 85.

Seo et al. [47] tested compounds **344** and **345** for glycosidase inhibitory activity against α-glucosidase, α-amylase, and β-amylase. The α-glucosidase activities (IC_50_ values) of **344** and **345** were 12.4 µM and 15.6 µM, respectively. IC_50_ values of **344** against α-amylase and β-amylase were 37.3 µM and 201.4 µM, respectively, and that of **345** against α- and β-amylase were 16.8 µM and 24.8 µM, respectively. These results suggested that the activities of compounds **344** and **345** were stronger against all three enzymes, with **345** being the more potent against α- and β-amylases than others. Both **344** and **345** exhibited weaker inhibition than **328b** against α-glucosidase. This means that position 3 on ring A for the sulfonamide group is more favorable only for inhibition of α- and β-amylases. Overall, the activity of compounds **344** and **345** was much better than other chalcone derivatives assessed [47].

Kim et al. [79] evaluated compounds **344** and **345** for the inhibition of trans-sialidase from *Trypanosoma cruzi* (TcTS), a parasite that causes Chagas disease. The concentration required for 50% inhibition was found to be 37 µM and 2.5 µM for **344** and **345**, respectively. Differing comparisons can be drawn for each of the compounds **344** and **345** when put next to their corresponding 4-aminochalcone analog **328b** and **329b**. For monohydroxy substituents like **344** and **328b**, a better inhibitory activity was observed by **344**; however, strangely for dihydroxy substituents **345** and **329b,** the trend was reversed, with **329b** showing better activity (IC_50_ = 0.9 µM) than **345**. Overall, the activities of dihydroxy-substituted aminochalcones were much better than monohydroxy substituted aminochalcones, and this sharp difference earned most of the attention in the discussion of results [79].

## 7. Concluding Remarks 

The focus of this review has been to accumulate aminochalcones in one place and to list their pharmacological activities. During this review, we have attempted to draw comparisons between the biological activities of these aminochalcones. However, a clear trend for the activities between the types of aminochalcones was not observed. Many compounds discussed had activities comparable to, if not better than, the reference drugs against which they were tested. While these investigations have paved a future for the pharmacological significance of aminochalcones, further study is needed to optimize and develop molecules based on these leads. One thing that is clear at this stage is that the pharmacological activities of aminochalcones have not been utilized in the industry as they can be. There is still a molecule to hit the clinical trials. At the same time, there is room to focus on the structure optimization for certain cellular targets. While there have been some reports of the activity of these compounds on microtubules, AChE, BChE, tyrosinase, etc., there is a need to have dedicated drug-discovery programs to optimize the structures, especially considering that covalent inhibitors are an emerging area of drug discovery. We hope that this review will serve as a beacon to provide information about aminochalcones in one place for the scientific community to ponder the reported activities, to design subsequent structure optimizations, and to stimulate further advances in the field.

## Figures and Tables

**Table 1:** Inhibitory effect of compound **1** against the human Molt 4/C8; CEM T-lymphocytes; murine P388; L1210; human cervix carcinoma HeLa; murine mammary carcinoma FM3A; estrogen-receptor-positive breast cancer cells MCF-7; triple-negative breast cancer (TNBC) cells MDA-MB-231; human ovarian cancer cells Caov-3; human lung cancer cells A549; human liver cancer cells HepG2; human nasopharyngeal cancer cells CNE-1; human T-lymphoblastoid leukemia cells CEM-SS; breast cancer cells T47D; leukemia cell lines K562 and HL-60; human colon cancer HT-29, LS180, LoVo, and LoVo/DX cell lines; and normal green monkey fibroblast line COS7.

Cell Line	IC_50_	Reference
Molt 4/C8	8.57 ± 1.25 µM	[36]
	7.9 ± 0.8 µM	[37]
CEM	8.29 ± 0.45 µM	[36]
	8.5 ± 1.5 µM	[37]
P388	5.25 ± 0.2 µM	[36]
L1210	9.14 ± 0.74 µM	[36]
	14 ± 3 µM	[37]
HeLa	7.7 ± 0.6 µM	[37]
	>100 µM	[38]
FM3A	34 ± 1 µM	[37]
MCF-7	>100 µM	[39]
	19.60 ± 3.45 µM	[38]
MDA-MB-231	60.3 ± 2.9 µM	[39]
	42.84 ± 3.20 µM	[38]
Caov-3	>100 µM	[38]
A549	69.16 ± 2.64 µM	[38]
HepG2	72.97 ± 3.63 µM	[38]
CNE-1	78.10 ± 6.50 µM	[38]
CEM-SS	28.71 ± 7.97 µM	[38]
T47D	5.28 µg/mL (23.6 µM) *	[40]
HL-60	9.63 ± 2.32 µg/mL (43.1 ± 10.4 µM) *	[41]
K562	5.87 ± 0.15 µg/mL (26.3 ± 0.7 µM) *	[41]
	42.54 ± 1.58 µM	[38]
HT-29	1.98 ± 0.18 µg/mL (8.9 ± 0.8 µM) *	[42]
	>100 µM	[38]
LS180	4.68 ± 0.09 µg/mL (21.0 ± 0.4 µM) *	[42]
LoVo	2.84 ± 0.09 µg/mL (12.7 ± 0.4 µM) *	[42]
LoVo/DX	2.26 ± 0.05 µg/mL (10.1 ± 0.2 µM) *	[42]
COS7	3.85 ± 0.28 µg/mL (17.2 ± 1.3 µM) *	[42]

* Values (in µM) were calculated by us for uniformity using the µg/mL values provided in the respective papers.

**Table 2:** Inhibitory effect of **1** against two strains of bacteria *Escherichia coli* ATCC10536 *Staphylococcus aureus* DSM 799, the yeast of strain *Candida albicans* DSM1386, and three strains of fungi: *Alternaria alternata* CBS1526, *Fusarium linii* KB-F1, and *Aspergillus niger* DSM1957.

Strain	Minimal Inhibitory Concentrations (mg/mL)
*E. coli* ATCC10536	0.5
*S. aureus* DSM799	0.25
*C. albicans* DSM1386	0.5
*A. alternata* CBS1526	0.5
*F. linii* KB-F1	0.25
*A. niger* DSM1957	0.5

**Table 3:** Inhibitory effect of compound **2** against human Molt 4/C8, CEM T-lymphocytes, murine P388, and L1210.

Cell Line	IC_50_	Reference
Molt 4/C8	8.38 ± 1.26 µM	[36]
CEM	8.48 ± 0.65 µM	[36]
P388	6.25 ± 0.9 µM	[36]
L1210	10.04 ± 1.6 µM	[36]

**Table 4:** IC_50_ values of methoxy-aminochalcones (**3a**–**j**) against murine leukemia (L1210), murine mammary carcinoma (FM3A), human T-leukemia (Molt/4 and CEM), human cervix carcinoma (HeLa) cells, breast cancer cells T47D, and leukemia cell lines K562 and HL-60.

Compound	Molt 4/C8 [37] (µM)	CEM [37] (µM)	L1210 [37] (µM)	HeLa [37] (µM)	FM3A [37] (µM)	T47D [40] (µg/mL)	HL-60 [41] (µg/mL)	K562 [41] (µg/mL)
**3a**	8.6 ± 0.4	14 ± 3	18 ± 5	8.7 ± 0.3	35 ± 6	11.66	6.92 ± 1.18	48.45 ± 5.37
**3b**	8.3 ± 0.9	6.3 ± 1	12 ± 1	7.8 ± 0.3	25 ± 5	15.95	1.76 ± 0.20	10.93 ± 0.67
**3c**	21 ± 11	23 ± 15	22 ± 15	19 ± 14	45 ± 10	11.96	7.81 ± 1.12	21.46 ± 0.30
**3d**	8.5 ± 0.3	7.6 ± 1.0	13 ± 8	6.8 ± 0.0	29 ± 2	8.93	1.57 ± 0.40	8.65 ± 0.93
**3e**	20 ± 11	18 ± 7	34 ± 12	22 ± 6	43 ± 0	35.40	2.49 ± 0.84	52.56 ± 1.79
**3f**	-	-	-	-	-	8.11	1.84 ± 0.41	11.84 ± 1.11
**3g**	7.5 ± 0.1	5.7 ± 0.5	6.0 ± 4.2	7.9 ± 0.7	12 ± 1	-	-	-
**3h**	8.6 ± 0.2	9.3 ± 0.2	25 ± 17	7.8 ± 0.1	35 ± 3	-	-	-
**3i**	5.6 ± 1.2	2.3 ± 0.3	7.8 ± 0.1	6.9 ± 0.8	8.8 ± 0	-	-	-
**3j**	10 ± 1	9.7 ± 0.6	25 ± 16	8.2 ± 0.9	29 ± 3	-	-	-
**1**	7.9 ± 0.8	8.5 ± 1.5	14 ± 3	7.7 ± 0.6	34 ± 1	5.28	9.63 ± 2.32	5.87 ± 0.15

**Table 5:** Inhibitory effect of compounds **5a**–**c** and **5e** against the human Molt 4/C8, CEM T-lymphocytes, murine P388, L1210, estrogen-receptor-positive breast cancer cells MCF-7, and the triple-negative breast cancer (TNBC) cells MDA-MB-231.

Cell Line	5a	5b	5c	5d	5e
Molt 4/C8 [34] (µM)	7.70 ± 0.32	-	-	-	8.69 ± 1.75
CEM [34] (µM)	7.44 ± 0.76	-	-	-	6.41 ± 0.12
P388 [34] (µM)	3.44 ± 0.2	-	-	-	3.94 ± 0.1
L1210 [34] (µM)	8.62 ± 0.54	-	-	-	8.22 ± 0.20
MCF-7 [39](µM) MCF-7 [38] (µM)	>100 17.46 ± 4.30	nd	34.2 ± 6.4	>100	-
MDA-MB-231 [39] (µM)	69.0 ± 8.5	nd	>100		-
HeLa [38] (µM)	23.51 ± 1.29	nd	nd	>100	nd
Caov-3 [38] (µM)	19.26 ± 0.99	nd	nd	93.24 ± 3.03	nd
A549 [38] (µM)	28.52 ± 4.60	nd	nd	94.08 ± 3.71	nd
HepG2 [38] (µM)	18.46 ± 3.88	nd	nd	>100	nd
HT-29 [38] (µM)	19.13 ± 4.67	nd	nd	>100	nd
CNE-1 [38] (µM)	38.56 ± 1.59	nd	nd	>100	nd
K562 [38] (µM)	10.16 ± 0.36	nd	nd	>100	nd
CEM-SS [38] (µM)	7.91 ± 0.83	nd	nd	29.82 ± 3.82	nd

nd: not determined.

**Table 6:** IC_50_ values of chloro-4-aminochalcones **5a**, **5b**, and **5d**, expressed in µg/mL.

Compound	Superoxide Radical	Hydroxyl Radical	ABTS Cation Radical	Lipid Peroxidation
**5a**	78.84 ± 1.99	170.18 ± 1.43	55.87 ± 0.63	547.57 ± 3.50
**5b**	88.25 ± 1.33	185.14 ± 1.62	78.86 ± 1.56	612.12 ± 2.31
**5d**	82.54 ± 1.57	207.21 ± 1.33	73.45 ± 1.69	650.32 ± 1.54

**Table 7:** Inhibitory effect of compound **8** against the human Molt 4/C8, CEM T-lymphocytes, murine P388, L1210, human colon cancer HT-29, LS180, LoVo, and LoVo/DX cell lines.

Cell Line	IC_50_		Reference
	8	1	
Molt 4/C8	7.28 ± 0.74 µM	8.57 ± 1.25 µM	[36]
CEM	8.44 ± 0.59 µM	8.29 ± 0.45 µM	[36]
P388	3.70 ± 0.3 µM	5.25 ± 0.2 µM	[36]
L1210	6.09 ± 3.02 µM	9.14 ± 0.74 µM	[36]
HT-29	3.42 ± 0.54 µg/mL	1.98 ± 0.18 µg/mL	[42]
LS180	4.64 ± 0.54 µg/mL	4.68 ± 0.09 µg/mL	[42]
LoVo	1.80 ± 0.10 µg/mL	2.84 ± 0.09 µg/mL	[42]
LoVo/DX	2.00 ± 0.12 µg/mL	2.26 ± 0.05 µg/mL	[42]

**Table 8:** IC_50_ values of compounds **10**–**13** against the human colon cancer HT-29, LS180, LoVo, and LoVo/DX cell lines in µg/mL.

Compound	HT-29	LS180	LoVo	LoVo/DX
**10**	7.83 ± 0.14	8.23 ± 0.49	7.64 ± 0.53	6.66 ± 0.48
**11**	5.01 ± 0.49	4.59 ± 0.29	3.54 ± 0.30	nd
**12**	3.61 ± 0.21	5.20 ± 0.30	4.26 ± 0.19	3.68 0.30
**13**	32.42 ± 0.99	18.37 ± 0.19	73.54 ± 0.98	66.18 1.32

nd: not detected in the tested concentration.

**Table 9:** IC_50_ values of chloro-4-aminochalcones **17a**, **17b**, and **17c**, expressed in µg/mL.

Compound	Superoxide Radical	Hydroxyl Radical	ABTS Cation Radical	Lipid Peroxidation
**17a**	88.31 ± 1.34	185.21 ± 1.35	77.01 ± 2.06	624.46 ± 1.92
**17b**	88.87 ± 1.53	189.63 ± 2.18	77.22 ± 1.38	617.74 ± 2.32
**17c**	89.65 ± 2.01	191.35 ± 2.03	75.36 ± 2.31	604.47 ± 1.29

**Table 10:** Inhibitory effect of compound **26** against the human colon cancer HT-29, LS180, LoVo, and LoVo/DX cell lines; estrogen-receptor-positive breast cancer cells MCF-7; triple-negative breast cancer (TNBC) cells MDA-MB-231; human cervical cancer cells HeLa; human ovarian cancer cells Caov-3; human lung cancer cells A549; human liver cancer cells HepG2; human nasopharyngeal cancer cells CNE-1; human T-lymphoblastoid leukemia cells CEM-SS; human erythromyeloblastoid leukemia cells K562; and normal human embryonic kidney HEK-293 cells.

Cell Lines	IC_50_ Values
HT-29	1.43 ± 0.16 µg/mL (6.4 ± 0.7 µM) * 4.39 ± 0.25 µM
LS180	2.06 ± 0.18 µg/mL (9.2 ± 0.8 µM) *
LoVo	1.56 ± 0.04 µg/mL (7.0 ± 0.2 µM) *
LoVo/DX	1.43 ± 0.02 µg/mL (6.4 ± 0.1 µM) *
COS7	2.64 ± 0.47 µg/mL (11.8 ± 2.1 µM) *
MCF7	5.42 ± 0.17 µM
MDA-MB-231	>100 µM
HeLa	>100 µM
Caov-3	44.35 ± 2.34 µM
A549	5.58 ± 0.12 µM
HepG2	11.43 ± 0.45 µM
CNE-1	63.03 ± 5.83 µM
K562	4.99 ± 0.11 µM
CEM-SS	10.29 ± 3.30 µM
HEK-293	>100 µM

* Values (in µM) were calculated by us for uniformity using the µg/mL values provided in the respective papers.

**Table 11:** Inhibitory effect of compound **29** against the estrogen-receptor-positive breast cancer cells MCF-7, triple-negative breast cancer (TNBC) cells MDA-MB-231, human cervical cancer cells HeLa, human ovarian cancer cells Caov-3, human lung cancer cells A549, human liver cancer cells HepG2, human colorectal cancer cells HT-29, human nasopharyngeal cancer cells CNE-1, human T-lymphoblastoid leukemia cells CEM-SS, human erythromyeloblastoid leukemia cells K562, and normal human embryonic kidney HEK-293 cells.

Cell Line	IC_50_ Values (µM)
MCF7	14.44 ± 4.65
MDA-MB-231	>100
HeLa	>100
Caov-3	26.45 ± 6.41
A549	>100
HepG2	72.11 ± 2.67
HT-29	5.93 ± 1.56
CNE-1	95.27 ± 3.66
K562	36.29 ± 2.92
CEM-SS	36.62 ± 6.86
HEK-293	>100

**Table 12:** Anticancer activity of 2-aminochalcones **32b**, **33**, **35**, and **36** (IC_50_ values).

Cell Line	32b	33	35	36	References
HT-29 (µg/mL) (µM)	3.24 ± 0.27 5.36 ± 0.17	3.98 ± 0.03	4.85 ± 0.08	4.26 ± 0.13	[9,41]
LS180 (µg/mL)	2.00 ± 0.35	5.10 ± 0.65	6.64 ± 0.29	7.86 ± 0.35	[9]
LoVo (µg/mL)	1.11 ± 0.07	4.77 ± 0.37	5.77 ± 0.38	7.68 ± 0.34	[9]
LoVo/DX (µg/mL)	0.96 ± 0.12	4.78 ± 0.19	4.59 ± 0.21	11.08 ± 0.44	[9]
COS7 (µg/mL)	1.79 ± 0.14	9.57 ± 0.52	9.02 ± 0.17	7.78 ± 0.75	[9]
MCF7 (µM)	8.15 ± 1.03	-	-	-	[41]
MDA-MB-231 (µM)	8.27 ± 4.69	-	-	-	[41]
HeLa (µM)	45.90 ± 3.17	-	-	-	[41]
Caov-3 (µM)	15.58 ± 0.88	-	-	-	[41]
A549 (µM)	10.97 ± 1.89	-	-	-	[41]
HepG2 (µM)	13.08 ± 3.22	-	-	-	[41]
CNE-1 (µM)	46.15 ± 3.69	-	-	-	[41]
K562 (µM)	8.84 ± 0.38	-	-	-	[41]
CEM-SS (µM)	15.11 ± 3.12	-	-	-	[41]
HEK-293 (µM)	36.31 ± 3.02	-	-	-	[41]

**Table 13:** Inhibitory activities of **26**, **27**, **29**, **29a**, **30c**–**d**, **31b**, **32b**, and **36a**–**d** against AChE and BACE-1.

Compound	AChE (IC_50_, µM)	BACE-1 (IC_50_, µM)
**26**	84.11	43.22
**27**	2.92	nd
**29**	19.02	8.34
**29a**	14.09	6.39
**30c**	0.08	2.71
**30d**	96.07	nd
**31b**	28.31	23.94
**32b**	95.11	22.18
**36a**	1.22	nd
**36b**	4.16	nd
**36c**	0.54	19.63
**36d**	28.06	nd
**Tacrine**	0.06	-
**Quercetine**	-	1.91

nd: not determined.

**Table 14:** Cytotoxic activities (ED_50_ values µg/mL) of compounds **41**–**44b** against the epidermoid carcinoma of the nasopharynx KB, P-gp-expressing epidermoid carcinoma of the masopharynx KB-VIN, osteosarcoma Hos, melanoma SKMEL-2, ileocecal carcinoma HCT-8, breast cancer MCF-7, lung carcinoma A-549, glioblastoma U87-MG, and ovarian cancer 1A9 cell lines.

Compound	KB	KB-VIN	HOS	SKMEL-2	HCT-8	MCF-7	A-549	U87-MG	1A9
**41**	1.35	1.25	4.00	5.10	9.80	6.10	3.50	>10 (34)	1.20
**42**	0.65	0.30	2.00	3.50	4.50	1.30	1.20	5.10	0.80
**43**	0.52	0.30	2.40	2.30	1.50	0.48	0.45	8.00	0.35
**44a**	1.50	0.95	4.00	6.50	3.00	2.60	2.00	7.00	1.50
**44b**	3.10	2.20	6.00	9.00	6.50	5.10	4.20	>10 (49)	3.50

**Table 15:** Inhibitory effect of compounds **48**–**53** against the human colon cancer HT-29, LS180, LoVo, and LoVo/DX cell lines and the COS7 normal cell line (IC_50_ values (µg/mL)).

Compound	HT-29	LS180	LoVo	LoVo/DX	COS7
**48**	1.60 ± 0.01	2.13 ± 0.18	1.88 ± 0.12	1.97 ± 0.30	3.25 ± 0.66
**49**	3.43 ± 0.05	4.16 ± 0.12	1.79 ± 0.10	2.35 ± 0.08	4.42 ± 0.27
**50**	12.32 ± 0.32	9.22 ± 0.72	31.50 ± 0.13	27.44 ± 0.11	15.93 ± 1.04
**51**	2.77 ± 0.49	2.89 ± 0.53	1.34 ± 0.10	1.36 ± 0.06	2.30 ± 0.45
**52**	7.54 ± 0.18	6.51 ± 0.62	-	5.19 ± 0.41	7.61 ± 0.43
**53**	2.90 ± 0.27	3.76 ± 0.38	1.71 ± 0.13	1.77 ± 0.09	3.45 ± 0.22

**Table 16:** Minimal inhibitory concentration (MIC) values (mg/mL) of **48**–**53** against two bacterial strains, *Escherichia coli* ATCC10536 *Staphylococcus aureus* DSM 799; a yeast strain, *Candida albicans* DSM1386; and three fungal strains, *Alternaria alternata* CBS1526, *Fusarium linii* KB-F1, and *Aspergillus niger* DSM1957.

Strain	48	49	50	51	52	53
*E. coli* ATCC10536	0.25	1.0	0.125	1.0	0.0625	0.125
*S. aureus* DSM799	0.25	-	0.125	0.25	-	-
*C. albicans* DSM1386	0.5	-	-	0.25	-	-
*A. alternata* CBS1526	0.5	-	-	-	-	1.0
*F. linii* KB-F1	-	-	-	0.5	-	-
*A. niger* DSM1957	-	-	-	1.0	-	1.0

**Table 17:** Cytotoxic effect of compounds **55**–**58** against murine melanoma B16 cells (IC_50_ values in µM)**.**

Compound	IC_50_ Values (µM)
**55**	>100
**56a**	43
**56b**	33
**57a**	8.8
**57b**	18
**57c**	5.6
**58**	67

**Table 18:** Cytotoxic effect of compounds **60**, **61**, **62a**–**c**, **63**, **64a**, **64b**, and **65**–**69** against murine leukemia L1210, murine mammary carcinoma FM3A, human T-leukemia Molt/4 and CEM, and human cervix carcinoma HeLa cancer cells (IC_50_ values in µM)**.**

Compound	L1210	FM3A	Molt4	CEM	HeLa
**60**	88 ± 12	146 ± 96	10 ± 2	34 ± 1	36 ± 1
**61**	49 ± 12	107 ± 25	12 ± 7	31 ± 3	40 ± 1
**62a**	160 ± 15	282 ± 44	19 ± 3	37 ± 8	98 ± 64
**62b**	38 ± 15	43 ± 0	20 ± 15	26 ± 24	33 ± 6
**62c**	14 ± 3	36 ± 6	1.5 ± 0.3	2.5 ± 0.2	1.9 ± 0.2
**63**	21 ± 4	55 ± 0	11 ± 1	15 ± 0	41 ± 4
**64a**	111 ± 15	178 ± 30	7.8 ± 1.9	21 ± 17	43 ± 1
**64b**	26 ± 1	33 ± 0	13 ± 0	15 ± 0	31 ± 9
**65**	123 ± 92	185 ± 3	12 ± 2	39 ± 0	40 ± 4
**66**	90 ± 12	120 ± 3	10 ± 0	14 ± 1	49 ± 2
**67**	50 ± 4	110 ± 9	7.5 ± 0.8	16 ± 1	58 ± 11
**68**	32 ± 9	41 ± 4	12 ± 2	22 ± 14	18 ± 4
**69**	87 ± 7	123 ± 20	13 ± 2	27 ± 19	15 ± 3

**Table 19:** Comparison of the IC_50_ values of **1** and **60** against the murine leukemia L1210, murine mammary carcinoma FM3A, human T-leukemia Molt/4 and CEM, and human cervix carcinoma HeLa cancer cells (IC_50_ values in µM).

Cell Line	1	60
L1210	14 ± 3	88 ± 12
FM3A	34 ± 1	146 ± 96
Molt4	7.9 ± 0.8	10 ± 2
CEM	8.5 ± 1.5	34 ± 1
HeLa	7.7 ± 0.6	36 ± 1

**Table 20:** IC_50_ (in µM) values of **81** against human colon carcinoma cells HCT116, human hepatocellular liver carcinoma cells HepG2, prostate DU145, skin B16-F10, breast MDA-MB-231, and A549 and the cervical Hela cancer cell lines, along with the normal LO2 cell line and the drug-resistant HCT-8/T**.**

Cell Line	IC_50_
HCT116	0.28 ± 0.06
HepG2	0.19 ± 0.04
DU145	0.018 ± 0.001
B16-F10	1.79 ± 0.23
MDA-MB-231	0.53 ± 0.08
A549	1.45 ± 0.17
Hela	0.086 ± 0.003
LO2	6.39 ± 0.81
HCT-8/T	5.33 ± 0.86

**Table 21:** Antimicrobial activity of **83**–**88** tested against *Staphylococcus aureus* 209 p, *Escherichia coli* ESS 2231, *Proteus vulgaris*, *Klebsiella pneumoniae*, *Aspergillus fumigatus*, *Candida albicans*, *Candida krusei* GO3, and *Candida glabrata* HO3 expressed as MIC values in µg/mL.

Compound	*S. aureus*	*E. coli*	*Proteus vulgaris*	*Klebsiella pneumoniae*	*Aspergillus fumigatus*	*Candida albicans*	*C. albicans*	*Candida glabrata*
**83**	100	3.58	3.95	12.75	6.85	4.56	5.38	7.57
**84a**	3.13	5.62	3.85	18.64	5.56	6.89	5.69	9.94
**84b**	70	50	45	40	18.35	8.75	7.02	7.66
**85a**	5.25	10.28	45	55	35	9.12	50	16.25
**85b**	70	80	45	65	75	65	75	60
**86**	75	75	70	50	50	55	60	60
**87**	4.75	2.75	60	50	7.17	6.75	18.56	16.8
**88**	100	35	200	70	50	90	60	75

**Table 22:** Anti-plasmodial IC_50_ values for compounds **91** and **93**–**105**.

Compound	IC_50_ (µg/mL)
**91**	5.15
**93**	2.5
**94**	7.76
**95**	6.01
**96**	9.1
**97**	8.26
**98**	1.52
**99**	2.93
**100**	12.33
**101**	6.8
**102**	7.16
**103**	6.0
**104**	4.6
**105**	8.03

**Table 23:** MIC values for compounds **129**–**137** against the *S. aureus* ATCC33591 (resistant to methicillin), *E. faecium* 17501 (vancomycin-resistant clinical isolate), *E. faecium* ATCC29212, and *E. coli* ATCC25922 bacterial strains, expressed in µM.

Compound	*S. aureus* ATCC33591	*E. faecium* 17501	*E. faecium* ATCC29212	*E. coli* ATCC25922
**129**	5	10	10	20
**130**	5	5	5	10
**131**	10	10	10	20
**132**	2	5	5	5
**133**	20	20	40	20
**134**	20	40	40	40
**135**	20	40	40	75
**136**	75	75	75	-
**137**	75	75	150	-

**Table 24:** Inhibitory activity of compounds **141**–**145** against the production of NO and PGE_2_ in RAW 264.7 macrophages.

Compound	Nitrites		PGE_2_	
	%I	IC_50_ (µM)	%I	IC_50_ (µM)
**141**	49.9 ± 2.3	-	28.7 ± 8.6	-
**142**	32.2 ± 1.8	-	27.9 ± 6.3	-
**143**	49.1 ± 1.0	-	35.8 ± 4.1	-
**144a**	17.1 ± 2.4	-	34.6 ± 5.3	-
**144b**	73.2 ± 4.1	0.7	3.5 ± 3.5	-
**144c**	63.4 ± 5.0	0.6	73.3 ± 3.1	0.9
**145a**	25.4 ± 3.3	-	36.8 ± 1.7	-
**145b**	28.2 ± 2.6	-	44.9 ± 1.6	-
**145c**	39.9 ± 2.2	-	44.0 ± 3.3	-

**Table 25:** Inhibitory activity of compounds **144a**, **144c**, **143**, and **146**–**162** against the HeLa cells.

Compound	ED (µg/mL)	Compound	ED (µg/mL)
**143**	0.015	154a	0.0038
**144a**	6.25	154b	1.5
**144c**	0.0075	154c	0.019
**146**	0.15	155	3.1
**147a**	12.5	156	0.15
**147b**	0.015	157	>100
**148**	3.1	158a	0.0075
**149**	0.0075	158b	0.15
**150**	3.1	159a	0.0075
**151**	3.1	159b	25
**152a**	0.0075	160	0.15
**152b**	0.15	161	6.25
**152c**	0.0038	162	6.25
**153a**	0.015		
**153b**	0.015		
**153c**	3.1		

**Table 26:** AChE inhibition activity and oxygen radical absorption capacity (ORAC) of compounds **164**–**181**, expressed in Trolox equivalents.

Compound	IC_50_ (µM)	ORAC
	*Ee*AChE	
**164**	78. 21 ± 2.04	2.00 ± 0.03
**165**	32.62 ± 1.78	2.62 ± 0.05
**166**	17.21 ± 1.21	2.20 ± 0.02
**167**	4.91 ± 0.15	2.83 ± 0.05
**168**	6.90 ± 0.53	2.51 ± 0.05
**169**	8.01 ± 0.71	2.04 ± 0.04
**170**	14.33 ± 0.80	3.14 ± 0.05
**171**	11.94 ± 0.79	2.08 ± 0.04
**172**	8.62 ± 0.81	2.54 ± 0.02
**173**	21.43 ± 1.63	3.51 ± 0.09
**174**	>100	2.16 ± 0.04
**175**	30.02 ± 2.29	2.45 ± 0.05
**176**	32.44 ± 2.02	2.41 ± 0.03
**177**	58.01 ± 2.94	1.85 ± 0.04
**178**	11.74 ± 0.92	1.02 ± 0.02
**179**	14.06 ± 1.10	0.35 ± 0.01
**180**	10.62 ± 1.33	1.20 ± 0.02
**181**	63.50 ± 1.93	0.51 ± 0.01
**Rivastigmine**	9.94 ± 0.83	not tested

**Table 27:** Antimicrobial activity of **182**–**188** against Escherichia coli, Staphylococcus aureus, Salmonella typhi, Bacillus subtillis, Aspergillus niger, Aspergillus flavus, Penicillium chrysogenum, and Candida albicans strains, expressed as the zone of inhibition in mm.

Compound	Antibacterial Activity				Antifungal Activity			
	*E. Coli*	*S. Aureus*	*S. Typhi*	*B. Subtillis*	*A. Niger*	*A. Flavus*	*P. Chrysogenum*	*C. Albicans*
**182**	-	12	12	15	-	12	17	15
**183**	15	13	15	19	15	12	18	14
**184**	16	14	13	19	18	19	17	13
**185**	-	10	-	12	-	-	12	13
**186a**	14	12	15	18	-	13	16	14
**186b**	14	12	12	15	17	15	-	13
**187**	20	19	18	13	15	14	18	14
**188**	15	14	16	12	18	15	13	11
**Penicillin**	22	22	24	24	NA	NA	NA	NA
**Nystatin**	NA	NA	NA	NA	20	22	24	24

“-“ denotes “no activity”.

**Table 28:** Antiproliferative activity of **193**–**197** against human colon carcinoma cells HCT116 and human hepatocellular liver carcinoma cells HepG2.

Compound	IC_50_ (µM)	
	HCT116	HepG2
**193**	5.76 ± 0.38	5.25 ± 0.27
**194**	6.81 ± 0.25	7.12 ± 0.29
**195**	>10	>10
**196**	4.54 ± 0.23	5.09 ± 0.35
**197**	>10	>10

**Table 29:** Antibacterial activity of **198**–**203** against *S. aureus* ATCC33591 (resistant to methicillin), *E. faecium* 17501 (vancomycin-resistant clinical isolate), and *E. faecium* ATCC29212: activity is expressed as MIC values in µM.

Compound	*S. aureus* ATCC33591	*E. faecium* 17501	*E. faecium* ATCC29212
**198**	10	10	20
**199**	10	10	20
**200**	-	-	-
**201a**	5	10	20
**201b**	5	5	10
**201c**	-	-	-
**202**	75	75	75
**203**	-	-	-

**Table 30:** Inhibitory effect of compounds **204**–**207** and **209** against the human Molt 4/C8, CEM T-lymphocytes, and murine P388 and L1210 leukemic cells: activity is expressed as IC_50_ values in µM.

Compound	Molt 4/C8	CEM	P388	L1210
**204**	8.84 ± 0.02	8.30 ± 0.61	3.40 ± 0.1	9.05 ± 0.14
**205**	304 ± 105	223 ± 30	19.0 ± 0.6	175 ± 44
**206**	>500	343 ± 31	13.3 ± 0.2	320 ± 64
**207**	41.4 ± 1.1	39.4 ± 6.4	15.6 ± 1.2	35.9 ± 2.7
**209**	21.4 ± 1.1	21.1 ± 12.5	8.04 ± 1.1	19.6 ± 3.1

**Table 31:** IC_50_ values (µM) of compounds **211**–**217** for human Molt 4/C8, CEM T-lymphocytes, murine P388, and L1210 cells.

Compound	Human Molt 4/C8	CEM	P388	L1210
**211**	202 ± 8	186 ± 5	30.9 ± 1.2	160 ± 5
**212**	92.2 ± 61.9	108 ± 93	16.4 ± 0.5	68.4 ± 32.9
**213**	44.5 ± 1.8	43.5 ± 9.7	7.94 ± 1.6	25.0 ± 4.0
**214**	89.1 ± 59.2	126 ± 109	18.1 ± 1.3	72.0 ± 36.8
**215**	33.1 ± 5.8	34.2 ± 10.9	0.7 ± 0.03	26.6 ± 24.3
**216**	205 ± 20	211 ± 11	57.5 ± 0.1	165 ± 20
**217**	210 ± 3	239 ± 41	23.4 ± 2.7	205 ± 10

**Table 32:** In vitro inhibitory effects of compounds **219**–**223** on the proliferation of murine leukemia (L1210), murine mammary carcinoma (FM3A), human T-leukemia (Molt/4 and CEM), and human cervix carcinoma (HeLa) cells (IC_50_ values in µM).

Compound	L1210	FM3A	Molt4	CEM	HeLa
**219**	0.88 ± 0.59	0.69 ± 0.68	0.5 ± 0.17	1.5 ± 0.0	0.96 ± 0.34
**220a**	0.90 ± 0.63	1.0 ± 0.1	0.61 ± 0.55	1.1 ± 0.8	0.31 ± 0.00
**220b**	1.1 ± 0.6	1.8 ± 0.5	1.1 ± 0.1	1.8 ± 0.2	0.50 ± 0.00
**220c**	1.7 ± 0.3	0.37 ± 0.33	0.24 ± 0.20	0.58 ± 0.53	1.3 ± 0.2
**221a**	1.1 ± 0.9	0.55 ± 0.31	0.49 ± 0.07	0.71 ± 0.53	0.38 ± 0.03
**221b**	1.3 ± 0.9	1.2 ± 1.1	1.8 ± 0.2	1.6 ± 0.2	0.77 ± 0.36
**221c**	0.52 ± 0.05	1.2 ± 0.9	1.5 ± 0.8	1.1 ± 0.3	1.1 ± 1.0
**221d**	2.7 ± 0.7	2.0 ± 0.1	1.5 ± 0.0	2.2 ± 0.0	1.7 ± 0.3
**222a**	1.5 ± 0.9	1.8 ± 1.9	0.28 ± 0.08	0.33 ± 0.10	0.42 ± 0.00
**222b**	0.63 ± 0.12	0.53 ± 0.28	0.68 ± 0.26	0.84 ± 0.20	0.51 ± 0.00
**223**	1.9 ± 1.6	2.8 ± 1.5	1.3 ± 0.3	1.5 ± 0.2	0.61 ± 0.07

**Table 33:** IC_50_ values in µM of compounds **234**, **237**, **239**, **240**, **242**, **253**, **255**, and **256** against recombinant falcipain-2.

Compound	IC_50_ Values (µM)
**234**	3.8
**237**	2.6
**239**	2.6
**240**	2.5
**242**	1.8
**243**	2.5
**255**	3.4
**256**	12.5

**Table 34:** Inhibitory effects of compounds **260**–**269b** against *Staphylococcus aureus* ATCC 33591 (methicillin-resistant), *Staphylococcus aureus* ATCC 49951, *Staphylococcus aureus* ATCC29213, *Enterococcus faecalis* ATCC29212 (vancomycin sensitive), *Enterococcus faecalis* NCTC 12201 (vancomycin-resistant), and *Enterococcus faecium* ATCC 12202 (vancomycin-resistant) (MIC values in µg/mL).

Compound	*S. aureus* ATCC 33591	*S. aureus* ATCC 49951	*S. aureus* ATCC29213	*E. faecalis* ATCC29212	*E. faecalis* NCTC 12201	*E. faecium* ATCC 12202
**260**	>32	>32	>32	>32	>32	>32
**261**	>32	>32	>32	>32	>32	>32
**262**	>32	>32	>32	>32	>32	>32
**263**	>32	>32	>32	>32	>32	>32
**264**	>32	>32	>32	>32	>32	>32
**265**	32	32	32	32	32	32
**266**	32	32	32	64	64	64
**267a**	4	4	8	16	16	16
**267b**	0.25	1	0.5	2	1	2
**268a**	32	16	16	32	32	32
**268b**	8	8	8	16	16	16
**269a**	16	16	16	16	16	16
**269b**	32	32	32	32	32	32

**Table 35:** The concentration of compounds **287**–**296** giving the same mitotic index as colchicine (0.05 µg/mL), i.e., equivalent dose (ED) against HeLa cells exposed to the compounds for 6 h.

Compound	ED/6h (µg/mL)
**287**	>100
**288**	>100
**289**	25
**290**	0.25
**291**	3.1
**292**	6.25
**293a**	3.1
**293b**	3.1
**293c**	3.1
**294**	25
**295**	3.1
**296**	3.1
**62c**	0.15

**Table 36:** In vitro inhibitory effects of compounds **297**–**308** on the proliferation of murine leukemia (L1210), murine mammary carcinoma (FM3A), human T-leukemia (Molt/4 and CEM), and human cervix carcinoma (HeLa) cells (IC_50_ values expressed in μM).

Compound	L1210	FM3A	Molt4	CEM	HeLa
**297**	5.0 ± 1.7	3.9 ± 1.5	3.1 ± 1.4	4.0 ± 2.5	3.9 ± 1.5
**298**	1.6 ± 0.9	1.2 ± 0.3	0.66 ± 0.02	1.1 ± 0.9	1.4 ± 0.1
**299**	1.1 ± 0.2	1.3 ± 0.0	0.81 ± 0.33	0.95 ± 0.62	1.6 ± 0.2
**300**	2.1 ± 0.1	3.2 ± 1.1	1.8 ± 0.2	1.7 ± 0.1	3.2 ± 1.4
**301**	0.62 ± 0.20	1.1 ± 0.8	0.68 ± 0.24	0.99 ± 0.31	1.0 ± 0.0
**302**	3.4 ± 0.6	8.3 ± 2.6	2.8 ± 0.7	4.0 ± 2.4	7.7 ± 3.4
**303**	1.9 ± 0.4	2.1 ± 0.1	1.7 ± 0.1	1.8 ± 0.2	2.1 ± 0.7
**304a**	0.98 ± 0.96	1.2 ± 0.5	1.3 ± 1.2	1.4 ± 1.2	1.4 ± 0.2
**304b**	2.1 ± 1.5	2.7 ± 1.2	0.85 ± 0.06	2.3 ± 1.7	0.70 ± 0.10
**305**	0.91 ± 0.68	0.95 ± 0.77	0.85 ± 0.30	1.1 ± 0.8	1.1 ± 0.6
**306**	0.24 ± 0.05	0.68 ± 0.20	0.61 ± 0.17	0.75 ± 0.20	0.75 ± 0.05
**307**	1.1 ± 0.1	2.4 ± 0.8	2.1 ± 0.6	3.1 ± 1.3	2.0 ± 0.5
**308**	0.25 ± 0.11	0.52 ± 0.06	0.55 ± 0.18	0.73 ± 0.25	0.34 ± 0.12

**Table 37:** In vitro cytotoxic effects of compounds **316**–**321** against human colon carcinoma cells HCT116 and human hepatocellular liver carcinoma cells HepG2 (IC_50_ values expressed in µM).

Compound	HCT116	HepG2
**316**	1.47 ± 0.17	1.32 ± 0.11
**317**	1.54 ± 0.12	1.49 ± 0.08
**318**	1.48 ± 0.14	1.26 ± 0.07
**319**	>10	7.46 ± 0.51
**320**	>10	>10
**321**	>10	>10
**193**	5.76 ± 0.38	5.25 ± 0.27

**Table 38:** IC_50_ values in µM of compounds **322**–**327** against human liver cancer cell line HEPG2-2.

Compound	IC_50_ Values (µM)
**322**	-
**323**	49.8
**324**	39.3
**325**	43.3
**326**	26.0
**327**	30.3

**Table 39:** Inhibitory effect (IC_50_ values in µM) of compounds **322** and **328a**–**331** against β-secretase (BACE1) activity.

Compound	IC_50_ Values (µM)
**322**	168.7 ± 2.4
**328a**	6.28 ± 0.7
**328b**	1.44 ± 0.2
**328c**	2.88 ± 0.5
**328d**	79.3 ± 2.3
**328e**	5.58 ± 1.4
**328f**	107.4 ± 2.7
**328g**	119.6 ± 3.2
**329a**	4.59 ± 1.5
**329b**	0.21 ± 0.02
**329c**	0.62 ± 0.03
**329d**	0.69 ± 0.04
**329e**	101.3 ± 2.4
**329f**	8.95 ± 1.0
**330**	3.60 ± 0.3
**331**	16.87 ± 0.8

**Table 40:** Tyrosinase inhibitory activity (IC_50_ and Ki values in µM) and melanin formation (IC_50_ values in µM) of compounds **328a**–**c** and **328e**–**g**.

Compound	Tyrosinase Inhibition (Enzyme)		Melanin Formation (Cell)
	IC_50_ in µM	K_i_ in µM	IC_50_ in µM
**328a**	22.5	13.3	24.2
**328b**	23.3	12.6	10.4
**328c**	58.8	25.2	32.1
**328e**	30.7	16.7	21.2
**328f**	15.2	11.4	-
**328g**	29.1	14.9	19.8

**Table 41:** Effects (IC_50_ values in µM) of compounds **333**–**343** on inhibition of β-hematin formation and activity against cultured *Plasmodium falciparum* parasites.

Compound	Inhibition of β-Hematin Formation	Inhibition of *P. Falciparum* Parasites
	IC_50_ Values (µM)	IC_50_ Values (µM)
**333**	-	>10
**334**	-	1
**335**	-	>10
**336**	-	>10
**337**	-	>10
**338**	14.48	>10
**339**	8.65	>10
**340**	0.67	>10
**341**	-	>10
**342**	0.48	>10
**343**	0.50	>10
